# Circulating miR-29a and miR-150 correlate with delivered dose during thoracic radiation therapy for non-small cell lung cancer

**DOI:** 10.1186/s13014-016-0636-4

**Published:** 2016-04-27

**Authors:** Tru-Khang T. Dinh, Wojciech Fendler, Justyna Chałubińska-Fendler, Sanket S. Acharya, Colin O’Leary, Peter V. Deraska, Alan D. D’Andrea, Dipanjan Chowdhury, David Kozono

**Affiliations:** Harvard Medical School, 25 Shattuck St, Boston, MA 02115 USA; Department of Radiation Oncology, Dana-Farber Cancer Institute, 450 Brookline Ave, Boston, MA 02215 USA; Department of Biostatistics and Translational Medicine, Medical University of Łódź, Al. Kościuszki 4, 90-419 Łódź, Poland; Department of Radiation Oncology, Medical University of Łódź, Al. Kościuszki 4, 90-419 Łódź, Poland; Center for DNA Damage and Repair, Dana-Farber Cancer Institute, 450 Brookline Ave, Boston, MA 02215 USA; Department of Radiation Oncology, Brigham and Women’s Hospital, 75 Francis St, Boston, MA 02115 USA

**Keywords:** Non-small cell lung cancer, microRNAs, Radiotherapy, Thoracic

## Abstract

**Background:**

Risk of normal tissue toxicity limits the amount of thoracic radiation therapy (RT) that can be routinely prescribed to treat non-small cell lung cancer (NSCLC). An early biomarker of response to thoracic RT may provide a way to predict eventual toxicities—such as radiation pneumonitis—during treatment, thereby enabling dose adjustment before the symptomatic onset of late effects. MicroRNAs (miRNAs) were studied as potential serological biomarkers for thoracic RT. As a first step, we sought to identify miRNAs that correlate with delivered dose and standard dosimetric factors.

**Methods:**

We performed miRNA profiling of plasma samples obtained from five patients with Stage IIIA NSCLC at five dose-points each during radical thoracic RT. Candidate miRNAs were then assessed in samples from a separate cohort of 21 NSCLC patients receiving radical thoracic RT. To identify a cellular source of circulating miRNAs, we quantified in vitro miRNA expression intracellularly and within secreted exosomes in five NSCLC and stromal cell lines.

**Results:**

miRNA profiling of the discovery cohort identified ten circulating miRNAs that correlated with delivered RT dose as well as other dosimetric parameters such as lung V20. In the validation cohort, miR-29a-3p and miR-150-5p were reproducibly shown to decrease with increasing radiation dose. Expression of miR-29a-3p and miR-150-5p in secreted exosomes decreased with radiation. This was concomitant with an increase in intracellular levels, suggesting that exosomal export of these miRNAs may be downregulated in both NSCLC and stromal cells in response to radiation.

**Conclusions:**

miR-29a-3p and miR-150-5p were identified as circulating biomarkers that correlated with delivered RT dose. miR-150 has been reported to decrease in the circulation of mammals exposed to radiation while miR-29a has been associated with fibrosis in the human heart, lungs, and kidneys. One may therefore hypothesize that outlier levels of circulating miR-29a-3p and miR-150-5p may eventually help predict unexpected responses to radiation therapy, such as toxicity.

**Electronic supplementary material:**

The online version of this article (doi:10.1186/s13014-016-0636-4) contains supplementary material, which is available to authorized users.

## Background

Platinum-based chemotherapy with concurrent radiotherapy (RT) comprise first-line treatment for locally advanced NSCLC [1]. However, despite optimal chemoradiotherapy, approximately 40 % of patients suffer treatment failure within the irradiated area [2], which contributes to a dismal 15-25 % survival rate at 5 years [3, 4]. RTOG 0617 randomized patients to receive 60 vs. 74 Gy to determine whether dose escalation improves outcomes [5]. Unfortunately outcomes were worse in the higher dose arm due to increased toxicities and likely compromised tumor coverage to meet normal tissue constraints.

Factors that likely contribute to the high rate of locoregional failure include our inability to predict which patients may require dose escalation for local control or those who can tolerate higher doses to organs-at-risk. Despite complex variations in anatomic and biological characteristics between patients and their tumors, RT regimens have remained relatively uniform. RTOG 0617 established the standard radiation dose of 60 Gy given in 2 Gy daily fractions. In practice, prescriptions range between 60–70 Gy in 1.8 to 2.0 Gy fractions, depending on provider preferences and dosimetric constraints related to radiosensitive structures including the lungs, spinal cord, esophagus and heart [6]. Despite these constraints, 10–20 % of patients suffer moderate to severe radiation pneumonitis (RP) following standard treatment [7]. RP is an inflammatory response of the lungs to radiation resulting in symptoms of variable severity ranging from coughing, dyspnea and fever to life-threatening pulmonary failure. The 10–20 % figure is likely to be underestimated since the clinical manifestations of RP are non-specific. In severe cases, the RP-attributed mortality rate can be as high as 50 % [8].

Recently, microRNAs (miRNAs) have garnered interest as potential biomarkers for a range of biological and physiological states [9]. miRNAs are small (22–24 nucleotide), non-coding RNAs that decrease the expression levels of as many as 60 % of all coding sequences in the human genome [10, 11]. Circulating miRNAs are attractive as biomarkers of tumor radiation response and normal-tissue toxicity. They constitute the major fraction of small nucleic acids found in circulation, and despite high concentrations of RNA-degrading enzymes, circulating miRNA expression levels have been reproducibly measured to be stable [9]. They are also readily accessible through non-invasive blood testing. miRNA expression has been shown to specifically change in murine serum following whole body radiation [12]. In humans, miRNA expression in peripheral blood cells has been used to accurately distinguish pre- and post-radiation states [13]. While the mechanism underlying these changes remains unclear, miRNAs are involved in regulating the DNA damage response, making them especially relevant in the context of RT for NSCLC [14].

Despite being widely reported as potential biomarkers, the origins of circulating miRNAs are not fully understood. The majority of circulating miRNAs in plasma are found within exosomes, which help protect miRNAs from degradation [15]. Exosomes are small (50-80 nm) membrane vesicles of endocytic origin that are formed by the involution of endosomes into multivesicular bodies (MVB) [16]. Mature MVBs subsequently fuse with the plasma membrane to release exosomes from the cell in an orchestrated manner distinct from exocytosis. Exosomes containing miRNAs have been shown to mediate intercellular communication in processes such as antigen presentation and malignant transformation in EBV-associated lymphomas [17, 18].

In this study, we hypothesized that miRNA expression in the peripheral circulation may serve as biomarkers of radiation exposure during thoracic RT for NSCLC patients. We also investigated the potential cellular source of circulating miRNAs by measuring miRNA expression in exosomes isolated from conditioned media of cultured cells. These miRNAs may correspond to normal tissue and/or tumor response, allowing prediction of acute and delayed injury to these organs. With validation, such biomarkers have the potential to guide adaptive RT for NSCLC patients based on their biologic responses to radiation.

## Methods

### miRNA profiling and validation

Five plasma samples from each of five patients with Stage IIIA NSCLC undergoing radical chemoradiotherapy (profiling cohort) were collected before—and at approximately two-week intervals during—RT (Table 1). All patients received six MV photons and either 3D conformal or intensity modulated RT. 10 mL of blood were collected in spray coated potassium EDTA Vacutainer tubes (Fisher Scientific). Whole blood was transferred to 15 mL conical tubes (Falcon) and immediately spun at 2000 × g for 15 min at 4 °C. The resulting platelet-depleted plasma was aspirated and aliquoted into 500 μl volumes and stored in 1.5 mL cryovials (Corning) at -80 °C. Samples were comprehensively profiled for known human miRNAs (~1900) using Exiqon miRCURY LNA Universal RT microRNA PCR Human Panel I + II arrays (Exiqon, Copenhagen). Array data are included in the (Additional file 1: Table S1). Candidate miRNAs whose levels significantly correlated with RT dose, lung V5 and V20 (percent of lung volume receiving doses of at least 5 and 20 Gy, respectively), mean lung dose and mean esophagus dose were identified. miRNA candidates identified in the screen were validated using samples from an additional 21 NSCLC patients (validation cohort), collected before—and at 20 Gy intervals during—RT. Patients in the validation cohort had similar disease characteristics and treatment regimens (Table 2). One sample each from the 0 Gy and 40 Gy groups as well as two from the 20 Gy group had undetectable levels of miRNAs. Since we had no way of verifying whether this was due to technical error, sample storage or actual decrease of miRNA below the detection threshold we did not perform missing data imputation.

**Table 1:** Patient characteristics of profiling cohort

Patient	Age/Gender	Diagnosis	Indication/Type of treatment	Chemotherapy	RT dose
2468	76/F	IIIA SCC	definitive	KT	60.0 Gy
2510	61/F	IIIA ADENO	definitive	EP	66.0 Gy
2526	60/M	IIIA ADENO	neoadjuvant	EP	54.0 Gy
2534	60/F	IIIA SCC	definitive	EP	66.0 Gy
2561	76/M	IIIA ADENO	definitive	KT	66.0 Gy

*Abbreviations*: *SCC* squamous cell carcinoma, *ADENO* adenocarcinoma, *KT* carboplatin/paclitaxel, EP, etoposide/cisplatin

**Table 2:** Patient characteristics of validation cohort

Patient	Age/Gender	Diagnosis	Indication/Type of treatment	Chemotherapy	RT dose
AE1	58/F	IIA SCC	recurrence/adjuvant	PN	70.0 Gy
BE1	68/M	IIIA SCC	definitive	KG	66.0 Gy
CG1	55/M	IIA SCC	R2/adjuvant	PN	68.0 Gy
DW1	59/F	IIB SCC	R1/adjuvant	PN	68.0 Gy
FL1	76/M	IIIA SCC	definitive	PG	66.0 Gy
GH1	62/F	IIIB SCC	definitive	PN	66.0 Gy
GK1	67/M	IIIB SCC	definitive	KN	66.0 Gy
GW1	61/M	IIIA SCC	definitive	PG	66.0 Gy
KA1	64/F	IIIB ADENO	definitive	PN	58.0 Gy
KA2	64/M	IIB SCC	R1/adjuvant	PN	60.0 Gy
KI1	68/F	IIIA SCC	R1/adjuvant	KN	60.0 Gy
KM1	67/M	IIIA SCC	R1/adjuvant	PN	60.0 Gy
LJ1	77 M	IIIA SCC	R1/adjuvant	No chemo	56.0 Gy
MS1	70/M	IIIA SCC	R1/adjuvant	KN	66.0 Gy
PB1	60/M	IIIA NOS	definitive	PG	64.0 Gy
PI1	56/F	IIIB ADENO	definitive	PN	66.0 Gy
SB1	55/M	IIIA SCC	R1/adjuvant	PN	69.3 Gy
SJ1	55/M	IIB NOS	R1/adjuvant	PN	66.0 Gy
SW1	62/M	IIIB ADENO	definitive	PN	66.0 Gy
WJ1	73/M	IIIA ADENO	definitive	PG	60.0 Gy
ZA1	78/M	IIIA ADENO	definitive	KG	66.0 Gy

*Abbreviations*: *SCC* squamous cell carcinoma, *ADENO* adenocarcinoma, *NOS* not otherwise specified, *R1* positive microscopic margins, *R2* positive gross margins, *PN* cisplatin/vinorelbine, *KG* carboplatin/gemcitabine, *PG* cisplatin/gemcitabine, *KN* carboplatin/vinorelbine

Total RNA was isolated from 200 μL plasma samples using Exiqon miRCURY Biofluids Total RNA Isolation kits. RNA samples measurements quality control data are included in the (Additional file 1: Table S2). cDNA synthesis was performed using Exiqon Universal cDNA first-strand synthesis kits. Quantitative real-time PCR (Q-PCR) was performed using the Exiqon SYBR Green system in a 96-well format. All LNA primers were ordered from Exiqon and added at 250 nM final concentration. Q-PCR dCp data for the validation cohort are included in the (Additional file 1: Table S3).

For the profiling cohort samples, UniSp2, UniSp4, and UniSp5 were used as RNA isolation spike-in controls. UniSp6 was used as cDNA synthesis spike-in controls. Additionally, the DNA spike-in UniSp3 was added. In the validation cohort samples, UniSp3 and UniSp6 were used as spike-in controls. In all samples, spike-in controls were assayed at steady levels. The difference in miR-451 and miR-23a expression in plasma samples was used to assess hemolysis, with a cutoff of value of greater than eight [19].

### Measurement of radiation-responsive plasma miRNAs in cell lines and conditioned media

Human NSCLC cell lines NCI-H460 (large cell lung carcinoma), A549 (type II alveolar cell carcinoma) and NCI-H1299 (lung adenocarcinoma) were gifts from Dr. Matthew Meyerson. These lines were authenticated by Short Tandem Repeat (STR) profiling (ATCC). MRC5 (embryonic lung fibroblasts) and IMR90 (embryonic lung fibroblasts) were obtained directly from ATCC for this work and used in early passage [20–24]. Cells were grown in 10 cm tissue culture plates. NSCLC cells were cultured in Roswell Park Memorial Institute (RPMI) 1640 (Life Technologies, Carlsbad, USA) media. MRC5 and IMR90 cells were cultured in Eagle’s Modified Essential Media (ATCC, Manassas, USA). Media were supplemented with 10 % fetal bovine serum (FBS, Sigma). The same batch of FBS was used for all in-vitro experiments, which was profiled for miRNAs of interest and background normalization (Additional file 1: Figure S1).

For measurement of intracellular miRNA expression, cells were transferred to 6-well tissue-culture plates (Thermo Fisher, Waltham, USA) at a density of 10^5^ cells per well and allowed to reach log-phase growth in 48 h. Samples (*n* = 6 per group) were then irradiated with 2 Gy per day for 1, 2 or 3 days at a dose rate of 83 cGy per minute (2, 4 or 6 Gy total dose) using a Gammacell 40 Exactor (Best Theratronics, Ottawa, Canada). Cells were trypsinized and harvested 2 h after irradiation. Total RNA was immediately isolated from cell pellets using Exiqon miRCURY Plant and Cells Total RNA Isolation kits. RNA samples measurements quality control data are included in the (Additional file 1: Table S2).

For measurement of extracellular miRNA expression in conditioned media, cells were transferred to 15 cm tissue-culture plates (Thermofisher) at a density of 10^6^ cells per plate and allowed to reach log-phase growth in 48 h. Samples (*n* = 3 per group) were then irradiated at 2 Gy per day for 1 or 3 days (2 or 6 Gy total dose). Fresh media was added one hour prior to radiation on day 1 and media was not changed subsequently. After days 1 and 3, 10 mL conditioned media was collected from each plate 2 h after irradiation. Exosomes were isolated and purified using a solvent-exchange exosome isolation kit (Exiqon) before total RNA was isolated with Plant and Cells Total RNA Isolation kits. Isolation of exosomes was confirmed by Western blotting for Tsg101, a protein involved in multivesicular biogenesis [15, 25] (Additional file 1: Figure S2).

cDNA synthesis was performed using Exiqon universal cDNA first‐strand synthesis kits by adding 100 ng total RNA for intracellular samples. As established by Blondal et al., 6.5 μL total RNA was added for cDNA synthesis for exosome samples [19]. Quantitative RT‐PCR was performed using Exiqon SYBR Green master mix. All LNA primers except miR‐150‐5p were ordered from Exiqon and added at 250 nM final concentration. Q‐PCR was performed using an AB‐7500 thermocycler with a detection cutoff of 40 cycles. For miR‐150‐5p, the Qiagen miSCRIPT SYBR Green primer assay was used, reverse transcription (RT‐PCR) was performed using the miSCRIPT RT Kit (Qiagen) and Q‐PCR was performed using the miSCRIPT SYBR Green Kit (Qiagen), following manufacturer’s protocols.

For normalization of intracellular miRNA expression, the snRNA sequence U6 and miR-103a have been established as normalizers for quantification of intracellular miRNAs and were measured in addition to the two most stably expressed miRNAs from the initial screen, miR-let-7d and miR-16-2-3p [26]. miR-let-7a-5p has been shown to perform well as a normalizer for miRNA quantification of exosomes derived from cell culture and was measured to potentially supplement miR-let-7d and miR-16-2-3p [27]. NormFinder was used to find the most stably expressed pair, relative to miR-29a and miR-150, for normalization. Stability values are included in the (Additional file 1: Table S4).

Cell viability was determined using the trypan blue dye exclusion method, using a standard protocol [28]. Cell proliferation was assessed by manually counting adherent cells, averaged over five random high-powered fields (40×).

### Statistical methods

In the profiling experiment, miRNA expression data were normalized toward the average expression of miRNAs detectable in all samples. Using those miRNAs for normalization, we calculated dCp values by using the formula “dCp = Average Cp – miRNA of interest Cp.” Thus, higher scores represent higher expression levels. For in vitro experiments, fold changes of relative expression were calculated using the ΔΔC_t_ method [29].

In the profiling cohort, we used analysis of variance (ANOVA) to identify miRNAs whose expression differed significantly depending on radiation dose. The Benjamini-Hochberg step-up method was used to adjust significance values for multiple comparisons testing [30]. miRNAs that showed significance in ANOVA were selected for the validation experiments. miR-let-7d, miR-324, miR-16-2-3p and miR-126 were used as normalizers based upon their universal expression in all samples and high stability in the profiling dataset, as assessed by Normfinder [31].

Analysis in the validation cohort was performed using 63 samples from 21 patients treated for locally-advanced NSCLC to verify whether the observed differences replicate in that cohort as well. Given the larger number of patients and repeated measures at prespecified timepoints we were able to use a mixed model approach that evaluated the effect of the timepoint (fixed effect) and intraindividual differences in radiation response patterns (random effect). We performed pairwise comparisons between the baseline, 20 Gy and 40 Gy timepoints using the Newman-Keuls test.

We subsequently evaluated correlations between miRNA and dosimetry parameters to determine the pattern of changes of miRNAs differentially expressed in the profiling experiment. Pearson’s correlation was used to evaluate the direction and strength of such correlations.

For in-vitro data, we used Student’s *t*-test to compare miRNA expression levels between groups. In all cases, we assumed p levels < 0.05 to be statistically significant.

## Results

### Profiling of patient blood samples identifies two circulating miRNAs that inversely correlate with RT dose

**Fig. 1:**
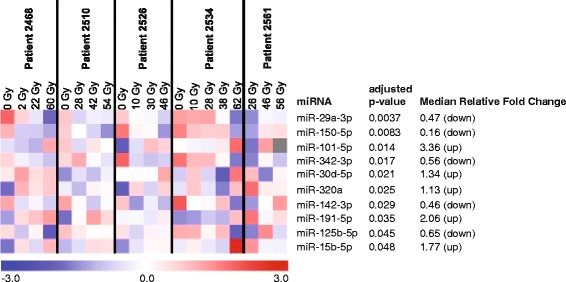
Heatmap of statistically significant miRNAs from microarray profiling of plasma samples obtained during RT. Of 124 universally expressed miRNAs, 10 were significantly correlated with RT dose. Five miRNAs decreased in expression in circulation with increasing radiation dose while five increased in expression. The median relative fold changes in miRNA expression among all five patients are shown. ANOVA adjusted p-values were calculated with the Benjamini-Hochberg procedure and represent false discovery rates

**Fig. 2:**
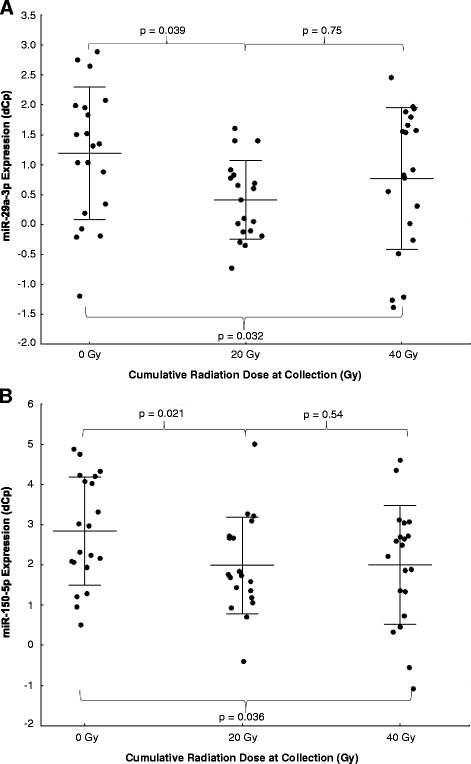
Validation of miR-29a-3p and miR-150-5p. (**a**) miR-29a-3p and (**b**) miR-150-5p expression significantly decreased in the circulation during the course of thoracic RT in the validation cohort (overall *p* = 0.032 and 0.036, respectively). *P*-values displayed are post-hoc comparisons between 0 and 20 Gy, 20 and 40 Gy, and 0 and 40 Gy. Higher dCp scores represent higher expression levels. Error bars represent standard deviation

Of 752 miRNAs measured in at least one sample in the profiling cohort, 124 were universally detected in all 25 samples. Four samples were excluded at pre-processing due to hemolysis. Ten miRNAs differed significantly (adjusted *p* < 0.05) depending on the received RT dose (Fig. 1). Of these ten miRNAs, miR-150-5p (*p* = 0.036) and miR-29a-3p (*p* = 0.032) (hereafter miR-150 and miR-29a) were shown to differ significantly depending on the received RT dose in the validation cohort (Fig. 2). The remaining eight candidate miRNAs identified in the profiling cohort did not reach statistical significance in the validation samples (Additional file 1: Table S5). As in the profiling cohort, circulating levels of miR-29a and miR-150 decreased with increasing RT dose. Despite a high variability of miRNA expression levels, the patterns remained convergent at intraindividual levels, confirming their specific reaction to RT. miR-29a and miR-150 also significantly correlated with clinically relevant dosimetric parameters: lung V5 and V20, mean lung dose and mean esophagus dose (Table 3).

**Table 3:**
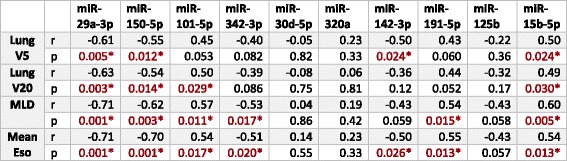
Correlation of miRNA expression and dosimetric parameters

Of 10 miRNAs significantly correlating with total dose, seven also significantly correlated with lung V5 (percent of lung volume receiving 5 Gy or greater), lung V20 (percent of lung volume receiving 20 Gy or higher), mean lung dose (MLD) and/or mean esophagus dose. Pearson correlation coefficient (r) and p-values are reported. Significant values are indicated in red with asterisks

### Intracellular levels of miR-29a-3p and miR-150-5p increase after irradiation in NSCLC cells and in lung fibroblasts

**Fig. 3:**
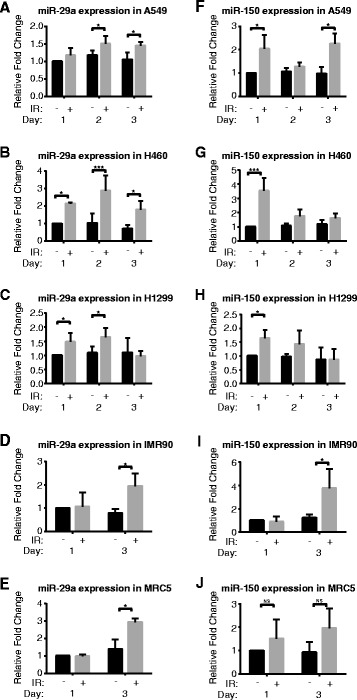
Intracellular expression of miR-29a and miR-150p. (**a-e**) miR-29a and (**f-j**) miR-150 levels increased with radiation in both NSCLC and stromal cell lines after 1, 2 or 3 days of 2 Gy fractions. Error bars represent standard deviation for *n* = 4 samples (*p < 0.05, ***p < 0.001)

In all three tested NSCLC cell lines, NCI-H460, A549 and NCI-H1299, radiation increased the intracellular expression of miR-29a and miR-150 as early as 2 h after irradiation (Fig. 3). This difference persisted with each fraction of radiation for up to 3 days of treatment. The same pattern of increase was also observed in two non-cancer cell lines, MRC5 and IMR90. For miR-29a, the relative increase in intracellular expression peaked after the second fraction of radiation, e.g. in H1299 cells where expression returned to baseline after the third fraction. For miR-150, the relative increase in intracellular expression did not express a clear temporal trend, although expression was typically highest after the first fraction of radiation. miR-let-7d, miR-16-2-3p and miR-103 demonstrated the highest Normfinder stability and were used for normalization. In concordance with recent studies, U6 routinely demonstrated low stability across groups, and was not used as a normalizer [26].

### miR-29a-3p and miR-150-5p levels are lower in exosomes secreted into the conditioned media of cells

**Fig. 4:**
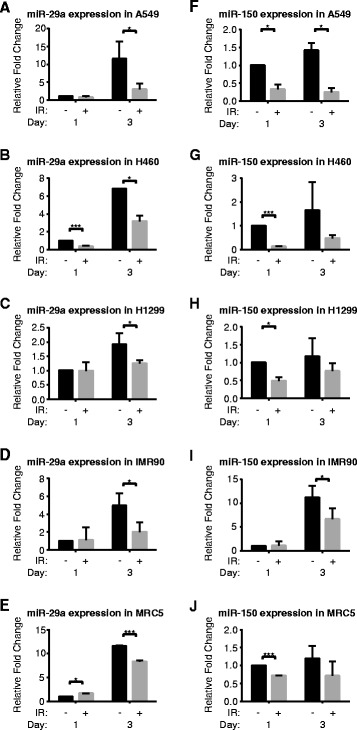
Exosomal expression of (**a-e**) miR-29a and (**f-j**) miR-150. Both miRNAs decreased in both NSCLC and stromal cell lines after 1 or 3 days of 2 Gy fractions. Error bars represent standard deviation for *n* = 3 samples (*p < 0.05, ***p < 0.001)

miR-29a and miR-150 levels were lower in exosomes purified from conditioned media of irradiated NCI-H460, A549 and NCI- H1299 cells compared to unirradiated control (Fig. 4). This difference persisted with each fraction of radiation for up to 3 days of treatment. Exosomal miR-29a and miR-150 were also lower for irradiated MRC5 and IMR90 cells (Fig. 4). At a 2 Gy fractional dose, radiation treatment abrogated proliferation in all cell lines, but did not significantly induce cell death (Additional file 1: Figure S3). miR-let-7a, miR-let-7d and miR-16-2-3p demonstrated high Normfinder stability and were used for normalization.

## Discussion

The Quantitative Analyses of Normal Tissue Effects in the Clinic (QUANTEC) study delineated dosimetric parameters, such as mean lung dose (MLD) and lung V20 that predict RP and other normal-tissue toxicities [6]. Unfortunately, the meta-analysis failed to identify any reliable threshold for RP. Indeed, rates of RP varied widely at any particular MLD or lung Vx. Thus, contemporary management is informed by institutional and protocol guidelines for these dosimetric parameters, based on what is felt to be acceptable risk to the average patient. In principle however, even if a dosimetric threshold could be reliably established, it would only provide a population-based estimator of RP risk. What would be more clinically useful would be a constitutive biomarker that dynamically estimates risk of RP over the course of radiation therapy. Whereas dosimetry is a proxy, we wish to interrogate the biology itself.

Recent efforts have focused on potential biochemical markers in the lung [32–35]. Anscher and colleagues attempted prospective dose-escalation in lung cancer patients whose serum TGF-β1 levels had renormalized at the end of standard-dose RT [36]. Unfortunately, many patients in the TGF-β1 -guided dose-escalation arm still experienced RP despite having TGF-β1 levels that predicted low risk of RP. Chen and colleagues found that circulating levels of IL-6 were higher after RT in a small group of patients who developed mild RP [37]. However, the sensitivity and specificity of IL-6 to retrospectively predict RP was limited to 78 % and 40 %, respectively [38]. Stenmark et al. attempted to combine dosimetric parameters with inflammatory cytokines in order to improve the prediction of RP [39]. They were able to slightly improve RP sensitivity (80 %), but still reported low specificity (60 %). These inflammatory factors may be limited as serological biomarkers due to the short half-lives of proteins in serum [40]. Circulating miRNAs are remarkably stable in serum, and may therefore offer better performance as biomarkers.

In this study, we identified two miRNAs that decreased in circulation with thoracic RT in patients with locally-advanced NSCLC. This two-miRNA signal remained significant despite inter-patient variability in our relatively small sample size. This may imply that these miRNAs are robust biomarkers, but does not preclude others that may become significant with increased statistical power. Due to institutional differences in treatment protocols, 10 of 21 patients in the validation cohort received radiation in the adjuvant setting while the remaining underwent definitive radical chemoradiotherapy. However, all patients in the profiling cohort underwent definitive radical chemoradiotherapy. This heterogeneity in the validation cohort may have altered the rate of change in lung or tumor-specific miRNAs, as we would expect irradiated volumes to be different in the adjuvant as compared to neoadjuvant setting. This may explain why only the two most significant signals identified in the profiling cohort remained significant in the validation cohort.

To determine the potential source of this circulating miRNA signal, we measured the expression of significant miRNAs intracellularly and within exosomes recovered from NSCLC and stromal cell culture. In harmony with both our screening and validation data of miRNAs found in circulation, radiation significantly diminished miR-29a and miR-150 accumulation in exosomes secreted into conditioned media. Interestingly, miRNA expression increased intracellularly with radiation. These patterns-while experimentally separate-suggest that the decrease in miR-29a and miR-150 levels in circulation may be a regulated process, rather than simply a reflection of decreasing intracellular miRNA expression. Both tumor and non-tumor cells may be downregulating the export of miRNAs, via decreased exosome secretion or decreased miRNA loading into secreted exosomes, leading to an intracellular buildup of miRNAs. Either mechanism would be an interesting effect of radiation worth further investigation. The decrease in relative exosomal export of miR-29a and miR-150 appears to hold for both NSCLC cells and lung stroma cells, although the miRNA expression patterns of other cell types found in the lung (e.g. lymphocytes and endothelial cells) were not measured. A limitation of these in vitro data is that intracellular and exosomal miRNA expression was not measured in the same experiment. Thus, the relationship between intracellular miRNA expression and exosomal miRNA expression is indirect. However, with the exception of larger culture plates to allow for the collection of sufficient conditioned media for exosome isolation, all other experimental conditions were identical.

Whether miRNAs circulate freely, are associated with ribonucleoprotein complexes or are encapsulated within exosomes remains unclear [15, 41, 42]. Notably, Arroyo et al. showed that miR-let-7a, miR-92a, and miR-142-3p circulate predominantly complexed to Argonaute-2 protein (a member of the RNA-induced silencing complex). Gallo et al. however subsequently found that these same miRNAs, as well as all miRNAs they tested in human serum, were much more enriched within purified exosomes. To our knowledge, no prior study specifically showed that circulating miR-29a or miR-150 is exosome associated. However we observed that these miRNAs had Ct values less than 35 cycles in exosome isolates but were undetectable in exosome-depleted samples (>40 cycles).

Notably, we observed that a 2 Gy fraction size was sub-lethal, whereas a lysis model of miRNA release would require significant cell death (Additional file 1: Figure S3). A conceivable alternate interpretation of the exosome expression pattern may be that radiation simply decreases cell proliferation, thereby leading to an apparent decrease in exosomal miRNA. This is unlikely because the significant relative decrease happens as early as 2 h after radiation, when there is a negligible difference in cell proliferation between irradiated and control samples.

Our results concur with previous studies by Templin and colleagues showing that miR-150 decreases in the blood of mice exposed to whole body radiation [12]. Our laboratory has also found that miR-150 decreases in the circulation of mice exposed specifically to thoracic radiation (unpublished data). miR-150 has been identified as an important promotor of inflammation via its regulation of MYB, an evolutionarily conserved regulator of hematopoiesis [43, 44]. Adams and colleagues demonstrated that over-expression of miR-150 impaired bone marrow reconstitution after hematopoietic ablation with 5-flourouracil [45]. In the context of circulating miRNAs, miR-150 may be a general marker of a leukocyte-driven inflammation in mammals exposed to radiation. To our knowledge, there has been no prior study linking miR-29a to radiation exposure. It is well described as an inhibitor of extracellular matrix (ECM) remodeling via its binding of 21 downstream proteins in the TGF-β/Smad3 pathway [46–48]. Reduction in miR-29a levels has been associated with bleomycin-induced lung fibrosis [47], ischemia-related myocardial fibrosis [46], and hypertension-associated renal fibrosis [48]. The decrease in circulating levels of miR-29a after thoracic radiation may indicate a pro-fibrotic state. Alternatively, the concomitant increase in intracellular miR-29a may be an adaptive response to radiation exposure. This has direct implications for miR-29a being a specific biomarker for predicting radiation pneumonitis. Indeed, miR-29a had higher statistical correlation with radiation dose compared to miR-150 in patients undergoing thoracic RT. Data from the Cancer Genome Atlas also reveals that miR-29a is expressed at much higher levels in both NSCLC tumors and normal lung tissue compared to miR-150 (Additional file 1: Figure S4).

## Conclusions

We show from an unbiased screen that miR-29a and miR-150 decrease in the circulation of NSCLC patients undergoing thoracic RT. Furthermore, this miRNA signal may originate—at least in part—from intracellular accumulation and concomitant reduction in exosome export from NSCLC and stromal cells. While miR-150 is likely a general biomarker of any tissue exposed to radiation, the reduction in circulating miR-29a may reflect a pro-fibrotic or adaptive state in the lung specifically. Patients whose levels of miR-29a decrease significantly more (in a pro-fibrosis model), or less (in an adaptive model), compared to average may be at higher risk for RP. Likewise, these differential expression patterns may reflect biological tumor response (or resistance) to radiation. Future studies will collect serum samples from a large number of NSCLC patients receiving RT in order to establish whether outlier trends in miR-29a and/or miR-150 corresponds to RP risk or tumor response. Establishing these miRNAs of interest as biomarkers for toxicity and/or tumor response may greatly facilitate patient-specific tailoring of RT.

### Ethics approval and consent to participate

All patients in the screening cohort gave informed consent per Dana-Farber/Harvard Cancer Center Protocol 02-180. All procedures were performed in accordance with local ethical standards and the Helsinki Declaration as revised in 2000.

### Consent for publication

Not applicable.

### Availability of data and materials

Raw miRNA profiling data are available for download as Supplementary Material.

## Additional file

Additional file 1: Table S1. Profiling cohort data. **Table S2.** RNA samples measurements. **Table S3.** Validation cohort Q‐PCR data. **Table S4.** NormFinder results. **Table S5.** Validation of miRNAs candidates. **Figure S1.** Background expression of miRNAs. **Figure S2.** Exosome isolation. **Figure S3.** Viability vs. IR status. **Figure S4.** miRNA expression in NSCLC and lung samples. (PDF 470 kb)

## Abbreviations

ANOVA: analysis of variance
FBS: fetal bovine serum
miRNA: microRNA
MLD: mean lung dose
MVB: multivesicular body
NSCLC: non-small cell lung cancer
RT: radiotherapy
