# Supplementary material for: Circulating miR-29a and miR-150 correlate with delivered dose during thoracic radiation therapy for non-small cell lung cancer
Source: Radiat Oncol. 2016 Apr 27;11:61. doi: 10.1186/s13014-016-0636-4 (PMC4847218; doi:10.1186/s13014-016-0636-4)
Supplement: Additional file 1: Table S1. — Profiling cohort data. Table S2. RNA samples measurements. Table S3. Validation cohort Q‐PCR data. Table S4. NormFinder results. Table S5. Validation of miRNAs candidates. Figure S1. Background expression of miRNAs. Figure S2. Exosome isolation. Figure S3. Viability vs. IR status. Figure S4. miRNA expression in NSCLC and lung samples. (PDF 470 kb) [file 13014_2016_636_MOESM1_ESM.pdf]

Table S1. Profiling cohort data. Raw Cp values are provided. ND = not detected.

**Patient ID**	**2468**				
**Dose**	**0 Gy**	**2 Gy**	**22 Gy**	**42 Gy**	**60 Gy**
**Date**	**2/12/2014**	**2/21/2014**	**3/7/2014**	**3/21/2014**	**4/30/2014**
cel-miR-39-3p CP	ND	ND	ND	36.8190824	35.2705236
hsa-let-7a-2-3p	ND	ND	ND	36.9393552	ND
hsa-let-7a-3p	37.7117161	35.1014265	37.377227	32.8416808	36.2466613
hsa-let-7a-5p	30.0077466	29.1916525	29.934848	26.3316618	28.1346745
hsa-let-7b-3p	33.1071292	33.4956809	34.848441	30.6300801	34.5058049
hsa-let-7b-5p	28.4908038	26.7101706	28.612645	22.6275801	25.4716187
hsa-let-7c	33.5914486	32.5558282	34.639615	29.9497764	32.0726795
hsa-let-7d-3p	29.6866671	29.4506739	30.443631	26.5380778	28.1790382
hsa-let-7d-5p	33.2886937	31.6028262	32.648896	27.9933073	30.5867194
hsa-let-7e-3p	ND	ND	ND	35.030379	ND
hsa-let-7e-5p	34.6550753	33.44794	37.027889	31.964025	33.9779891
hsa-let-7f-1-3p	ND	36.7911709	36.729562	33.1440456	35.4322006
hsa-let-7f-2-3p	ND	37.2308695	ND	34.762479	ND
hsa-let-7f-5p	33.914557	33.0400602	34.092609	30.2055809	32.322063
hsa-let-7g-3p	ND	35.1554524	ND	33.0379174	35.0491132
hsa-let-7g-5p	29.6673984	27.6332029	29.145524	23.4499961	26.5679159
hsa-let-7i-3p	36.1886932	37.8573098	36.531504	34.2953265	36.8955968
hsa-let-7i-5p	28.8597888	27.5647818	28.977151	23.6758875	26.1353507
hsa-miR-1	36.6842281	37.5118814	ND	37.1668814	37.3428885
hsa-miR-100-5p	32.9553178	33.1894702	33.818834	32.8315051	34.2524529
hsa-miR-101-3p	28.3338939	27.080823	29.32991	23.3926295	25.9669786
hsa-miR-101-5p	37.231045	36.5937624	38.420833	32.1046593	33.9396797
hsa-miR-103a-3p	29.7314821	28.1051975	29.228138	23.7858661	26.7185256
hsa-miR-105-3p	ND	ND	ND	ND	ND
hsa-miR-106a-3p	ND	ND	ND	ND	ND
hsa-miR-106a-5p	27.6843257	25.8878366	27.540641	21.7960398	24.0457886
hsa-miR-106b-3p	35.2347491	36.2159064	36.68104	32.1867492	35.2854059
hsa-miR-106b-5p	29.1136048	27.4444395	29.190846	23.0478671	26.3347562
hsa-miR-107	30.3326742	28.7708628	29.991467	24.7533727	27.4960362
hsa-miR-10a-5p	ND	37.7040085	38.726542	37.7789983	ND
hsa-miR-10b-5p	31.3326297	31.5641118	32.91026	31.7982353	32.6882066
hsa-miR-1181	ND	ND	ND	ND	37.4199569
hsa-miR-1183	ND	ND	ND	ND	ND
hsa-miR-1185-5p	ND	ND	ND	ND	ND
hsa-miR-1205	ND	ND	ND	ND	39.4411695
hsa-miR-1207-5p	ND	ND	ND	38.4416319	ND
hsa-miR-122-3p	ND	ND	ND	ND	ND
hsa-miR-1224-3p	ND	ND	ND	ND	ND
hsa-miR-122-5p	28.6877922	28.7371918	29.912407	29.0389004	28.6397954

hsa-miR-1227-3p	ND	ND	ND	36.2460946	ND
hsa-miR-1237-3p	37.0799595	ND	ND	ND	ND
hsa-miR-1238-3p	ND	ND	ND	36.8776052	ND
hsa-miR-1243	ND	ND	ND	ND	ND
hsa-miR-124-3p	ND	ND	ND	ND	ND
hsa-miR-1245a	ND	ND	ND	37.9425345	39.1936683
hsa-miR-1247-5p	ND	36.6072364	ND	38.3457722	ND
hsa-miR-1248	ND	ND	ND	ND	ND
hsa-miR-1249	ND	36.2718355	ND	37.4978624	ND
hsa-miR-1254	ND	ND	ND	ND	ND
hsa-miR-1255b-5p	38.1057073	ND	36.703817	33.4658774	35.8431288
hsa-miR-1256	ND	ND	ND	ND	ND
hsa-miR-125a-3p	ND	ND	ND	ND	ND
hsa-miR-125a-5p	33.271697	33.007828	33.385014	33.1899976	32.66548
hsa-miR-125b-2-3p	ND	35.8167806	ND	ND	36.0307559
hsa-miR-125b-5p	31.9638293	32.2520634	32.97963	30.7847029	32.2734877
hsa-miR-1260a	34.7732558	33.8077155	34.865694	33.6339551	33.4664982
hsa-miR-126-3p	28.0740475	27.5928416	28.848257	24.4573411	26.8293174
hsa-miR-1270	ND	ND	ND	34.9758486	35.8578578
hsa-miR-1271-5p	ND	ND	ND	35.5571737	ND
hsa-miR-127-3p	37.3464526	35.6866574	38.155734	ND	ND
hsa-miR-127-5p	ND	ND	ND	ND	ND
hsa-miR-128	32.0803085	30.607571	32.557916	26.9295383	ND
hsa-miR-129-5p	39.1081419	ND	ND	ND	ND
hsa-miR-1296	ND	ND	ND	ND	ND
hsa-miR-130a-3p	31.7784231	30.4753287	32.177839	27.1671801	29.5497836
hsa-miR-130b-3p	34.2400304	32.9408157	34.714708	29.8598474	31.9193005
hsa-miR-130b-5p	37.3195417	ND	ND	36.0594456	37.0796903
hsa-miR-132-3p	32.0692042	31.6349679	33.060155	29.8808249	31.9407336
hsa-miR-132-5p	ND	ND	ND	ND	ND
hsa-miR-133a	36.1459546	36.710467	35.147329	34.7867221	34.8655914
hsa-miR-133b	35.9247158	34.4736046	36.572908	37.6477993	35.7424584
hsa-miR-134	36.0596	37.2308422	37.692711	36.8991309	36.6327643
hsa-miR-135a-3p	ND	ND	ND	ND	ND
hsa-miR-135a-5p	ND	ND	37.722849	33.5911308	ND
hsa-miR-135b-5p	ND	ND	ND	ND	ND
hsa-miR-136-3p	37.7775728	ND	ND	ND	ND
hsa-miR-136-5p	36.0096726	36.2468831	ND	ND	36.4425486
hsa-miR-139-3p	36.6536123	ND	ND	37.1416883	ND
hsa-miR-139-5p	33.5717537	34.9773334	35.316583	34.182436	33.6482066
hsa-miR-140-3p	28.3153456	26.9685455	28.68265	23.1444636	25.5326636
hsa-miR-140-5p	31.8753058	31.7914727	33.142402	27.998198	29.9118308
hsa-miR-141-3p	36.0123998	35.9910635	36.506038	32.8704401	36.2495276
hsa-miR-141-5p	39.5637834	36.9374333	ND	ND	ND

hsa-miR-142-3p	29.5454846	29.8593415	31.199379	26.5717414	29.057098
hsa-miR-142-5p	32.2339926	31.9166315	33.854376	28.4545942	31.3569087
hsa-miR-143-3p	33.3402722	32.9175423	33.672724	32.2688685	32.7275249
hsa-miR-143-5p	ND	ND	ND	37.8674176	ND
hsa-miR-144-3p	27.0133599	25.8977115	27.934167	21.5078705	24.6978652
hsa-miR-144-5p	32.1900439	31.559469	32.311053	29.1609	29.6628569
hsa-miR-145-3p	ND	36.5804566	35.82256	35.5296096	37.4457759
hsa-miR-145-5p	31.9636658	30.8606614	32.854578	31.6094901	31.0549678
hsa-miR-1468	ND	35.697877	ND	34.9235488	ND
hsa-miR-146a-5p	29.7119997	30.0254914	31.024311	29.4799978	29.9883791
hsa-miR-146b-3p	ND	36.5137027	ND	36.3270358	ND
hsa-miR-146b-5p	35.5550216	33.8880738	35.333096	30.5963944	33.7341338
hsa-miR-1471	ND	ND	ND	ND	ND
hsa-miR-147b	ND	ND	ND	ND	ND
hsa-miR-148a-3p	30.7145445	29.983156	32.11192	26.7827905	29.2136079
hsa-miR-148b-3p	30.4355762	29.530946	31.518718	25.7745122	28.0247723
hsa-miR-148b-5p	ND	ND	ND	36.6617531	38.1797841
hsa-miR-149-5p	ND	ND	ND	ND	ND
hsa-miR-150-5p	30.0065527	31.3649894	32.946029	31.2058246	30.9093501
hsa-miR-151a-3p	31.9285295	31.2407253	33.075331	27.9916121	29.9542356
hsa-miR-151a-5p	31.7559105	30.8559494	32.537227	27.1358888	29.5876565
hsa-miR-152	32.7781212	31.490788	33.726967	28.6587314	30.8154303
hsa-miR-153	ND	ND	ND	36.5021098	ND
hsa-miR-1537	36.8845561	35.1626086	37.4011	33.2978845	35.2249508
hsa-miR-1538	ND	37.9770062	ND	ND	ND
hsa-miR-154-5p	ND	ND	ND	36.595405	ND
hsa-miR-155-5p	37.2869484	ND	ND	33.731201	36.0312815
hsa-miR-15a-3p	ND	36.4371381	ND	32.9941561	35.8420264
hsa-miR-15a-5p	25.7719303	24.0489459	26.098265	20.5291665	23.0742635
hsa-miR-15b-3p	31.3297069	30.0063986	31.933102	26.0826002	28.5984255
hsa-miR-15b-5p	30.0234176	28.0519018	30.204882	24.2059371	26.8629241
hsa-miR-16-1-3p	33.0682467	34.1874641	34.020745	29.9286839	32.3368817
hsa-miR-16-2-3p	31.4602801	30.1166992	32.144871	26.4952549	28.6394005
hsa-miR-16-5p	21.4330213	19.9924818	21.706667	16.3119275	18.5061121
hsa-miR-17-3p	36.8431299	34.4547716	35.624018	30.5875218	33.7839378
hsa-miR-17-5p	33.9211708	32.0470595	34.459921	27.7663421	30.204463
hsa-miR-181a-2-3p	ND	37.0363563	ND	36.6055662	ND
hsa-miR-181a-3p	ND	ND	ND	ND	ND
hsa-miR-181a-5p	32.9316469	31.8414522	33.591612	28.35584	31.3054227
hsa-miR-181b-5p	36.7810208	36.2221047	38.512581	33.8895107	35.3474845
hsa-miR-181c-3p	ND	35.5976079	ND	ND	ND
hsa-miR-181c-5p	ND	ND	ND	33.743119	36.3563467
hsa-miR-181d	ND	ND	ND	ND	ND
hsa-miR-182-3p	ND	ND	ND	34.8401458	ND

hsa-miR-182-5p	ND	37.291816	ND	32.2501038	34.8001051
hsa-miR-183-3p	38.9655982	ND	ND	31.9203173	35.2316006
hsa-miR-183-5p	37.9551275	34.2814638	35.279764	28.8212003	31.9901231
hsa-miR-184	ND	ND	ND	ND	ND
hsa-miR-185-3p	ND	37.889772	ND	34.5484213	35.1310022
hsa-miR-185-5p	28.7965762	26.6928434	28.689187	22.6546337	25.2659835
hsa-miR-186-5p	29.6009151	28.6323198	30.672062	24.7057554	27.0016808
hsa-miR-187-3p	ND	ND	35.698473	ND	ND
hsa-miR-187-5p	ND	ND	ND	ND	ND
hsa-miR-188-3p	ND	36.4104754	ND	35.3317773	ND
hsa-miR-188-5p	35.5074843	35.2812242	37.463176	32.6635774	33.9413904
hsa-miR-18a-3p	34.1448876	33.2951604	36.662364	28.584025	31.4336629
hsa-miR-18a-5p	32.3186282	30.1691468	31.534914	25.2250174	27.6942538
hsa-miR-18b-3p	ND	ND	ND	ND	ND
hsa-miR-18b-5p	31.1376046	29.5275689	31.007904	24.8871284	27.5679581
hsa-miR-1908	ND	ND	ND	34.8101543	36.8923277
hsa-miR-1909-3p	ND	ND	ND	ND	ND
hsa-miR-190a	ND	36.9250889	37.584521	34.1840131	37.0162194
hsa-miR-190b	36.8193537	39.2513281	ND	34.9421845	ND
hsa-miR-1912	ND	ND	ND	ND	ND
hsa-miR-1913	ND	ND	ND	37.0920996	ND
hsa-miR-191-3p	ND	37.4190086	ND	35.5300817	ND
hsa-miR-1914-5p	ND	ND	ND	ND	37.6372608
hsa-miR-191-5p	31.2636906	29.5447986	30.977653	25.7316761	28.2496877
hsa-miR-192-3p	ND	ND	ND	36.0027811	ND
hsa-miR-192-5p	30.6982443	29.3111808	31.514148	25.5354095	27.9315966
hsa-miR-193a-3p	ND	ND	ND	36.2316983	ND
hsa-miR-193a-5p	33.1452719	33.6673071	34.791808	32.7556017	33.7442658
hsa-miR-193b-3p	33.7610307	34.3209399	34.353632	36.1399886	36.9421291
hsa-miR-193b-5p	36.8739951	ND	37.880338	36.9161909	ND
hsa-miR-194-3p	ND	ND	ND	ND	ND
hsa-miR-194-5p	31.2530605	30.6206742	32.675697	27.1438515	29.7372125
hsa-miR-195-5p	35.0035003	34.296222	35.137543	31.8574788	33.3006668
hsa-miR-196a-5p	ND	ND	ND	34.5558481	37.8350044
hsa-miR-196b-3p	ND	ND	ND	33.9396722	37.966371
hsa-miR-196b-5p	ND	36.1532183	ND	31.5671273	34.7567673
hsa-miR-1972	34.3230849	35.1031407	35.905022	35.4787073	ND
hsa-miR-197-3p	31.8365478	32.2502558	33.587918	31.8979864	32.1424091
hsa-miR-199a-3p	31.2833381	31.4478413	32.178667	31.1461341	31.0069643
hsa-miR-199a-5p	35.5149408	ND	36.008984	35.7311418	34.43633
hsa-miR-199b-5p	ND	ND	ND	38.0590618	35.105095
hsa-miR-19a-3p	25.7766161	24.4225701	26.616806	20.5702572	23.1601042
hsa-miR-19a-5p	ND	ND	ND	36.4449112	ND
hsa-miR-19b-1-5p	ND	ND	36.241751	35.1759489	ND

hsa-miR-19b-3p	24.9267879	23.6710085	25.603936	19.6332958	22.2613998
hsa-miR-200a-3p	36.091074	36.043864	35.944216	34.1610138	35.9805641
hsa-miR-200b-3p	ND	37.7426469	36.248432	35.9735187	ND
hsa-miR-200b-5p	ND	ND	ND	38.0690446	ND
hsa-miR-200c-3p	ND	ND	ND	32.60459	34.2496718
hsa-miR-200c-5p	ND	ND	ND	ND	ND
hsa-miR-202-3p	ND	ND	ND	ND	ND
hsa-miR-202-5p	ND	ND	ND	35.0281675	36.1547052
hsa-miR-203a	37.0622124	ND	ND	36.4625228	ND
hsa-miR-204-5p	ND	ND	ND	36.553114	35.7111863
hsa-miR-205-5p	30.1895115	30.8560076	32.657822	32.6513929	35.7599318
hsa-miR-206	35.2320002	35.640487	35.282024	35.2484613	33.971704
hsa-miR-208b	ND	ND	ND	ND	ND
hsa-miR-20a-3p	36.5956391	ND	ND	31.6677426	34.7049483
hsa-miR-20a-5p	26.504432	24.8563121	26.557565	20.8606402	23.1618789
hsa-miR-20b-3p	ND	ND	36.945659	35.5098397	35.5107851
hsa-miR-20b-5p	35.9564009	33.038672	35.558235	29.357358	31.617894
hsa-miR-210	31.7345702	30.9531239	32.431479	27.2787957	29.2626868
hsa-miR-2110	36.1189969	33.7871222	33.601839	30.5814959	ND
hsa-miR-211-5p	ND	ND	ND	ND	ND
hsa-miR-212-3p	ND	35.2881573	37.145752	ND	36.3232018
hsa-miR-212-5p	ND	ND	ND	36.2451657	ND
hsa-miR-21-3p	ND	35.8143068	ND	34.5717145	38.533961
hsa-miR-214-3p	35.8507888	35.8129323	37.260885	36.8830014	35.8209583
hsa-miR-214-5p	35.8005249	ND	ND	ND	ND
hsa-miR-215	31.1723896	30.218155	32.199953	26.2596022	28.7833826
hsa-miR-21-5p	24.6513491	24.4726284	26.241036	21.7127763	23.8973817
hsa-miR-216a-5p	38.6658878	ND	ND	ND	36.9547526
hsa-miR-217	ND	36.9355581	ND	ND	36.7599091
hsa-miR-218-2-3p	ND	ND	ND	ND	ND
hsa-miR-218-5p	37.4394236	36.3287011	ND	39.6146688	ND
hsa-miR-219-1-3p	ND	ND	ND	ND	ND
hsa-miR-219-5p	ND	ND	ND	35.1034083	37.8865864
hsa-miR-221-3p	30.2959803	30.0018474	31.677422	29.5298177	29.8905595
hsa-miR-221-5p	37.037068	ND	ND	37.6453103	38.0613976
hsa-miR-222-3p	28.4639585	27.8283801	29.191162	24.9190429	26.8962004
hsa-miR-223-3p	25.7830491	25.3675155	27.029963	24.6447192	24.3476369
hsa-miR-223-5p	34.5839944	34.1525032	34.845472	34.446851	33.8769571
hsa-miR-22-3p	28.2512621	27.6775692	29.3566	24.5288761	26.5610791
hsa-miR-224-3p	ND	ND	ND	ND	ND
hsa-miR-224-5p	37.1554306	37.6595642	ND	ND	38.0273449
hsa-miR-22-5p	31.6573621	31.0529075	34.099338	28.6236604	31.1465687
hsa-miR-23a-3p	27.1339338	26.9155441	28.16081	26.626821	26.8975801
hsa-miR-23a-5p	ND	ND	ND	37.3008543	36.4373205

hsa-miR-23b-3p	30.201755	30.0536439	31.249343	29.4819659	30.1462661
hsa-miR-23b-5p	ND	ND	ND	ND	35.0985649
hsa-miR-24-1-5p	ND	ND	ND	ND	ND
hsa-miR-24-2-5p	ND	36.876595	35.889816	35.6907695	ND
hsa-miR-24-3p	28.1711637	28.1927817	28.983334	25.7048673	27.0995297
hsa-miR-25-3p	27.1323849	25.8008836	27.409749	21.8040694	24.023459
hsa-miR-25-5p	ND	ND	ND	33.8535137	ND
hsa-miR-26a-1-3p	ND	ND	36.832501	36.3313763	ND
hsa-miR-26a-2-3p	ND	ND	ND	ND	ND
hsa-miR-26a-5p	31.2616909	29.8136281	31.893176	26.1311389	28.8587049
hsa-miR-26b-3p	36.8851007	36.2060513	36.909401	33.658576	36.4587437
hsa-miR-26b-5p	31.4546767	29.7361818	31.512561	25.5889893	28.8456435
hsa-miR-27a-3p	31.3452706	30.9697221	32.349684	28.9036629	31.2126919
hsa-miR-27a-5p	37.1045317	36.8764656	38.000725	37.3689207	36.424535
hsa-miR-27b-3p	29.9218449	29.5797993	31.474576	28.5168485	29.9420072
hsa-miR-27b-5p	ND	ND	36.799949	ND	ND
hsa-miR-28-3p	34.8978219	36.4057264	35.497204	32.4520242	33.9254005
hsa-miR-28-5p	36.2449734	35.2555081	36.893941	33.260456	36.5170915
hsa-miR-296-3p	ND	ND	ND	ND	ND
hsa-miR-296-5p	34.5749786	34.0153697	36.227115	30.4907765	31.822476
hsa-miR-299-3p	ND	ND	ND	ND	ND
hsa-miR-299-5p	ND	ND	ND	ND	ND
hsa-miR-29a-3p	30.1423099	30.1186625	32.477079	29.5785172	30.9672331
hsa-miR-29a-5p	ND	36.4005832	ND	35.451811	37.6831496
hsa-miR-29b-2-5p	33.9432287	33.7193474	36.014653	31.0916736	32.7709977
hsa-miR-29b-3p	31.8030998	31.0606638	33.33388	26.3042412	29.7717193
hsa-miR-29c-3p	29.1444968	28.339319	30.546844	25.011093	27.7877234
hsa-miR-29c-5p	ND	37.4853889	36.444507	32.8696348	36.1060791
hsa-miR-300	ND	ND	ND	ND	ND
hsa-miR-301a-3p	33.5594975	32.35446	34.192699	27.5054821	30.4972846
hsa-miR-301b	ND	37.5918331	38.364589	33.7027794	35.2183481
hsa-miR-302c-5p	ND	ND	37.085182	ND	ND
hsa-miR-302d-5p	ND	ND	ND	ND	ND
hsa-miR-30a-3p	39.2924582	ND	37.31936	35.5990502	35.7994085
hsa-miR-30a-5p	33.4723029	32.2875176	32.814134	32.4312004	33.0590085
hsa-miR-30b-5p	31.9925256	31.2111009	32.576618	26.842786	29.8238285
hsa-miR-30c-2-3p	ND	ND	ND	ND	ND
hsa-miR-30c-5p	32.0132473	31.1736606	32.929089	28.0584046	30.1559746
hsa-miR-30d-3p	ND	36.7615138	ND	ND	ND
hsa-miR-30d-5p	33.1397458	31.8423237	33.747017	28.7846639	31.0996095
hsa-miR-30e-3p	35.9638333	34.8625556	ND	32.7095989	ND
hsa-miR-30e-5p	28.9801919	27.5041498	29.270136	23.5755118	25.9713313
hsa-miR-31-3p	36.5279882	ND	ND	ND	ND
hsa-miR-31-5p	ND	ND	ND	ND	ND

hsa-miR-320a	28.6943522	26.6162346	28.430998	23.6759573	25.7640367
hsa-miR-320b	30.2928581	28.4929083	30.765705	25.8550792	27.3276123
hsa-miR-320c	31.6248438	ND	ND	26.8901363	ND
hsa-miR-320d	30.3206688	28.5996474	30.305644	25.9714742	28.1545193
hsa-miR-323a-3p	ND	ND	ND	36.8897492	ND
hsa-miR-32-3p	ND	ND	ND	35.3073848	36.4943749
hsa-miR-324-3p	31.1785356	30.244551	31.803126	26.338841	28.7710449
hsa-miR-324-5p	34.7499545	33.0618951	34.442781	28.2042748	30.7181395
hsa-miR-32-5p	31.889032	30.2946195	33.530386	26.6969118	29.8266583
hsa-miR-326	ND	36.5291537	36.450636	35.8265125	36.20358
hsa-miR-328	34.54793	33.6830998	34.993324	31.1609315	32.2805785
hsa-miR-329	36.5690895	35.8099814	36.494362	ND	36.5181641
hsa-miR-330-3p	37.2928763	35.9291376	ND	34.7552908	ND
hsa-miR-330-5p	ND	ND	ND	ND	ND
hsa-miR-331-3p	38.1367059	35.6393157	36.832083	31.0425924	34.5934721
hsa-miR-331-5p	ND	ND	ND	38.3783763	ND
hsa-miR-335-3p	ND	ND	ND	34.5247152	35.5588149
hsa-miR-335-5p	33.9546491	32.5120476	34.222637	30.162954	32.2708551
hsa-miR-337-3p	ND	ND	ND	ND	ND
hsa-miR-337-5p	35.9605601	ND	ND	ND	ND
hsa-miR-338-3p	34.468612	33.8636738	37.224675	34.033242	33.6057355
hsa-miR-338-5p	38.9966436	37.7890143	ND	ND	37.8655173
hsa-miR-339-3p	34.717366	34.3033675	ND	31.064556	32.7202668
hsa-miR-339-5p	ND	37.3454062	ND	34.6624612	ND
hsa-miR-33a-3p	ND	ND	ND	35.697289	ND
hsa-miR-33a-5p	36.2992278	39.5283895	ND	35.3037458	35.5581862
hsa-miR-33b-3p	ND	ND	ND	36.6305641	ND
hsa-miR-33b-5p	35.9068976	35.3557929	ND	31.7605184	34.2181056
hsa-miR-340-3p	ND	37.7217875	ND	36.0726808	ND
hsa-miR-340-5p	ND	ND	ND	ND	38.3141088
hsa-miR-342-3p	31.6378469	31.2537034	32.963614	29.4244707	30.6445743
hsa-miR-342-5p	ND	35.195829	ND	32.378907	35.6775899
hsa-miR-345-5p	ND	ND	ND	34.8220318	37.8690247
hsa-miR-346	ND	36.9008207	ND	ND	35.774279
hsa-miR-34a-3p	39.2199555	37.0316697	ND	34.6209551	39.3056389
hsa-miR-34a-5p	32.3629417	32.1541601	32.154649	29.142798	31.0256381
hsa-miR-34b-3p	ND	ND	ND	ND	ND
hsa-miR-34c-5p	ND	ND	ND	ND	ND
hsa-miR-361-3p	36.0678988	35.975043	35.917833	31.5431074	33.8028425
hsa-miR-361-5p	31.4930069	31.6297444	32.244696	29.1846744	30.5692579
hsa-miR-362-3p	33.783111	33.0209498	36.495055	29.1080854	31.9927065
hsa-miR-362-5p	38.4872272	36.9516125	38.159898	34.8791901	36.0974959
hsa-miR-363-3p	30.0625506	28.6461498	30.64569	24.9027676	27.1130814
hsa-miR-363-5p	ND	ND	ND	ND	ND

hsa-miR-365a-3p	33.9980758	33.8754336	34.509763	32.1385818	33.1218902
hsa-miR-369-3p	ND	ND	ND	ND	ND
hsa-miR-369-5p	ND	ND	ND	ND	ND
hsa-miR-370	ND	ND	ND	ND	ND
hsa-miR-373-3p	ND	ND	ND	ND	ND
hsa-miR-373-5p	ND	38.906992	ND	36.905351	39.603984
hsa-miR-374a-5p	34.095241	33.5902246	34.980083	28.3373103	32.6794471
hsa-miR-374b-3p	ND	ND	ND	34.8073941	36.8918247
hsa-miR-374b-5p	35.5723693	34.5544802	36.062597	30.0740127	34.3211429
hsa-miR-375	32.9719159	33.4852503	32.789746	34.182674	33.2872843
hsa-miR-376a-3p	ND	ND	ND	39.1285517	37.8002285
hsa-miR-376a-5p	ND	ND	ND	ND	ND
hsa-miR-376b-3p	ND	ND	ND	ND	ND
hsa-miR-376c-3p	35.8503674	37.3759911	37.380771	36.4845243	35.7105738
hsa-miR-377-3p	36.250955	ND	ND	ND	ND
hsa-miR-377-5p	ND	ND	ND	ND	ND
hsa-miR-378a-3p	30.8738985	30.2554646	31.538598	26.6173985	28.6627996
hsa-miR-378a-5p	35.0187186	34.890998	36.068657	31.9647875	34.136226
hsa-miR-379-3p	ND	ND	ND	ND	ND
hsa-miR-379-5p	ND	ND	ND	ND	ND
hsa-miR-380-3p	ND	ND	ND	ND	ND
hsa-miR-381-3p	ND	ND	ND	ND	ND
hsa-miR-382-3p	ND	ND	ND	ND	ND
hsa-miR-382-5p	ND	36.121766	35.598667	36.545038	ND
hsa-miR-409-3p	ND	37.0304405	36.26075	ND	ND
hsa-miR-409-5p	ND	ND	ND	36.4883584	ND
hsa-miR-410	ND	ND	ND	ND	36.8965783
hsa-miR-411-5p	37.0347146	ND	ND	ND	ND
hsa-miR-412	ND	ND	ND	ND	ND
hsa-miR-421	34.5084171	36.0763769	35.986094	30.7953087	33.2894901
hsa-miR-423-3p	31.9266136	32.1854623	32.772481	26.6192705	28.777504
hsa-miR-423-5p	29.9151459	28.7016076	30.52773	25.5168184	27.136487
hsa-miR-424-3p	39.0283923	35.8827455	37.842715	32.045109	33.7145487
hsa-miR-424-5p	31.0016768	30.0387129	32.325122	26.3062285	29.4763925
hsa-miR-425-3p	33.6634157	33.1219741	ND	28.8050219	31.6818715
hsa-miR-425-5p	28.9481217	27.831772	29.72033	24.2230956	26.8567523
hsa-miR-429	ND	ND	36.981868	ND	ND
hsa-miR-431-3p	ND	ND	ND	ND	ND
hsa-miR-431-5p	ND	ND	ND	ND	37.9329863
hsa-miR-432-3p	ND	ND	ND	ND	ND
hsa-miR-432-5p	ND	ND	ND	ND	ND
hsa-miR-433	ND	ND	ND	37.7570566	ND
hsa-miR-449a	ND	ND	ND	35.199288	36.5306882
hsa-miR-449b-5p	ND	ND	ND	ND	ND

hsa-miR-450a-5p	ND	ND	ND	38.4350396	ND
hsa-miR-450b-3p	ND	ND	ND	ND	ND
hsa-miR-450b-5p	ND	34.9332964	35.201639	34.636247	36.6960292
hsa-miR-451a	20.6626676	19.5127929	21.310076	15.3071815	18.452505
hsa-miR-452-5p	ND	ND	36.853223	ND	ND
hsa-miR-454-3p	36.4358579	33.2991241	33.692443	27.8277497	30.9207503
hsa-miR-454-5p	36.8005068	37.351564	ND	36.0104072	ND
hsa-miR-455-3p	ND	ND	36.218035	35.9502986	35.600209
hsa-miR-455-5p	ND	ND	ND	ND	ND
hsa-miR-483-3p	ND	ND	35.98606	38.4123933	35.4587282
hsa-miR-483-5p	35.0752527	33.8112839	36.050023	36.8222531	35.3419276
hsa-miR-484	29.9797775	28.5245189	30.431957	24.8513848	26.8010638
hsa-miR-486-3p	34.8916652	32.8268915	34.553051	28.661511	30.8371619
hsa-miR-486-5p	26.3383821	24.5694472	26.539804	20.8277528	23.0147581
hsa-miR-487a	ND	ND	ND	ND	ND
hsa-miR-487b	ND	ND	ND	37.7736444	ND
hsa-miR-489	ND	ND	ND	ND	ND
hsa-miR-490-3p	33.7320451	32.7315973	33.530973	33.2632208	32.8397487
hsa-miR-490-5p	ND	ND	ND	ND	ND
hsa-miR-491-5p	36.18465	ND	ND	ND	36.2951119
hsa-miR-493-3p	ND	ND	ND	ND	ND
hsa-miR-493-5p	ND	ND	ND	ND	ND
hsa-miR-494	ND	ND	ND	ND	ND
hsa-miR-495-3p	34.9182898	36.4288309	35.16954	35.5085139	35.8179354
hsa-miR-496	ND	ND	ND	ND	ND
hsa-miR-497-5p	32.8534463	33.4642409	33.2168	32.6545474	33.3138858
hsa-miR-499a-5p	ND	ND	ND	36.1986871	36.4324743
hsa-miR-500a-5p	ND	36.241399	37.800273	31.1554959	34.1751048
hsa-miR-501-3p	34.289911	33.8039035	34.662706	30.9851763	32.7576902
hsa-miR-501-5p	34.4516298	36.1633474	36.77322	30.1900758	32.9407574
hsa-miR-502-3p	32.8887368	32.6073942	33.321289	28.8651683	31.0996024
hsa-miR-502-5p	ND	39.1326643	ND	31.9381443	34.8631042
hsa-miR-503-5p	ND	ND	ND	35.8138093	36.5609512
hsa-miR-505-3p	33.2872588	33.2626195	34.868049	30.0244218	32.2942614
hsa-miR-505-5p	39.3209427	35.5615772	38.110005	31.7471996	34.5378984
hsa-miR-507	ND	ND	ND	ND	ND
hsa-miR-508-3p	ND	ND	ND	ND	ND
hsa-miR-509-3-5p	ND	ND	ND	ND	ND
hsa-miR-509-3p	33.69007	ND	ND	ND	ND
hsa-miR-511	35.5454619	36.893073	ND	35.9968298	36.0984484
hsa-miR-513a-5p	ND	ND	ND	ND	ND
hsa-miR-514a-3p	ND	ND	ND	ND	ND
hsa-miR-515-3p	ND	ND	ND	ND	ND
hsa-miR-517c-3p	ND	ND	ND	ND	ND

hsa-miR-518d-3p	ND	38.0657143	ND	ND	38.4385677
hsa-miR-518f-3p	ND	36.6785435	ND	ND	ND
hsa-miR-518f-5p	ND	ND	ND	ND	ND
hsa-miR-519b-3p	ND	ND	ND	ND	ND
hsa-miR-520a-5p	ND	ND	ND	ND	ND
hsa-miR-520c-3p	ND	ND	ND	ND	ND
hsa-miR-520g	ND	ND	ND	ND	ND
hsa-miR-520h	36.9603158	ND	39.519022	ND	ND
hsa-miR-524-3p	ND	ND	ND	ND	ND
hsa-miR-525-3p	ND	ND	ND	ND	ND
hsa-miR-525-5p	ND	ND	ND	ND	35.8829117
hsa-miR-532-3p	33.0958163	32.176651	33.968208	28.8914634	31.2147629
hsa-miR-532-5p	32.129625	30.6207593	31.970337	26.8372538	29.082432
hsa-miR-539-5p	ND	ND	ND	ND	ND
hsa-miR-542-5p	ND	ND	ND	34.1274771	35.7444315
hsa-miR-543	ND	ND	ND	ND	ND
hsa-miR-544a	ND	ND	ND	ND	ND
hsa-miR-545-3p	ND	38.730018	ND	32.9254528	36.7740414
hsa-miR-548a-3p	ND	ND	32.061916	37.6264117	ND
hsa-miR-548a-5p	ND	ND	ND	ND	ND
hsa-miR-548b-3p	ND	ND	ND	ND	ND
hsa-miR-548c-5p	ND	ND	ND	38.4850263	35.9488096
hsa-miR-548d-3p	ND	37.6956373	ND	37.1055288	38.783162
hsa-miR-548d-5p	ND	ND	ND	36.8153778	36.6674898
hsa-miR-548e	ND	ND	ND	ND	ND
hsa-miR-548j	ND	35.2656152	ND	34.2967155	35.6492635
hsa-miR-548k	ND	ND	ND	36.431051	36.4127111
hsa-miR-548l	38.1787503	ND	ND	37.0187078	ND
hsa-miR-548n	ND	36.7300572	ND	35.2635394	ND
hsa-miR-549a	ND	ND	ND	ND	ND
hsa-miR-550a-3p	34.1069553	32.966188	35.454252	29.2422255	32.0607714
hsa-miR-550a-5p	ND	36.2055902	ND	33.0032386	34.8223636
hsa-miR-551a	ND	ND	35.812476	35.7210453	ND
hsa-miR-551b-3p	ND	ND	ND	36.4810857	36.1714266
hsa-miR-551b-5p	ND	38.3106335	ND	38.2430055	ND
hsa-miR-556-3p	ND	ND	ND	ND	ND
hsa-miR-564	ND	ND	ND	ND	ND
hsa-miR-570-3p	35.9431633	37.1236949	ND	32.656525	35.5352776
hsa-miR-571	ND	ND	ND	ND	37.9084304
hsa-miR-573	ND	ND	ND	ND	ND
hsa-miR-574-3p	33.4367386	32.5092262	34.038407	30.1709424	31.3136484
hsa-miR-576-3p	ND	36.1173462	37.397177	33.6382504	38.4695294
hsa-miR-576-5p	ND	ND	ND	32.6296527	ND
hsa-miR-579	36.4150383	36.3114206	ND	33.0836425	35.6822795

hsa-miR-580	ND	ND	ND	ND	ND
hsa-miR-581	ND	ND	ND	ND	ND
hsa-miR-582-5p	ND	ND	ND	36.8742131	35.7660545
hsa-miR-584-5p	34.7398372	34.4676856	35.154719	30.9524806	32.4531392
hsa-miR-589-3p	ND	ND	ND	37.783233	ND
hsa-miR-589-5p	ND	ND	ND	35.473957	37.3999763
hsa-miR-590-3p	35.6844285	38.6855082	ND	32.113342	35.1194438
hsa-miR-590-5p	32.3199642	30.9044118	33.518835	27.6787378	30.1268172
hsa-miR-596	ND	ND	ND	ND	37.7576539
hsa-miR-597	ND	ND	ND	39.3253634	ND
hsa-miR-598	35.9023062	35.1527599	37.695951	30.7561456	33.2368288
hsa-miR-601	ND	ND	ND	ND	ND
hsa-miR-604	ND	ND	ND	ND	ND
hsa-miR-605	ND	ND	ND	ND	ND
hsa-miR-610	ND	36.2958379	ND	34.6891019	38.3611413
hsa-miR-612	ND	ND	ND	ND	ND
hsa-miR-615-3p	ND	ND	ND	ND	ND
hsa-miR-616-5p	37.1804032	35.9313147	ND	33.523081	35.5215091
hsa-miR-618	ND	ND	ND	ND	ND
hsa-miR-621	ND	ND	ND	ND	ND
hsa-miR-624-5p	32.9991157	31.7279556	32.698125	29.54888	31.1296961
hsa-miR-625-3p	36.6372661	36.6856098	ND	32.6522126	35.0911089
hsa-miR-626	ND	ND	ND	ND	ND
hsa-miR-627	ND	36.2988274	ND	33.795759	ND
hsa-miR-628-3p	38.2213389	35.6667828	ND	32.5591896	35.1808882
hsa-miR-628-5p	ND	ND	37.187903	37.3145816	ND
hsa-miR-629-3p	ND	ND	36.213066	34.4874447	37.7665849
hsa-miR-629-5p	32.7786433	31.6377794	34.088655	28.056411	29.9819887
hsa-miR-636	ND	ND	ND	31.5510919	ND
hsa-miR-641	ND	ND	ND	ND	ND
hsa-miR-642a-5p	ND	37.8020036	ND	ND	ND
hsa-miR-643	ND	36.1124916	ND	33.7562644	38.7197987
hsa-miR-650	ND	ND	ND	ND	ND
hsa-miR-651	ND	34.6919086	37.424206	32.7329728	33.9063836
hsa-miR-652-3p	31.8908892	30.175481	31.758729	26.1467749	28.6242649
hsa-miR-654-3p	ND	ND	ND	ND	ND
hsa-miR-654-5p	ND	ND	ND	ND	ND
hsa-miR-655	ND	36.792701	36.55039	36.5224463	35.6686416
hsa-miR-659-3p	ND	ND	ND	ND	ND
hsa-miR-660-5p	29.9686757	29.3284841	31.210821	25.7144722	28.0395012
hsa-miR-662	ND	ND	ND	ND	37.818075
hsa-miR-663a	37.8547462	35.5794268	39.438535	34.9815976	35.2554285
hsa-miR-664a-3p	36.7351068	36.6058618	38.210015	34.4852876	38.5583691
hsa-miR-665	ND	ND	36.837301	ND	ND

hsa-miR-668	ND	ND	ND	ND	ND
hsa-miR-671-3p	ND	ND	ND	ND	36.0721624
hsa-miR-671-5p	ND	ND	ND	ND	ND
hsa-miR-675-3p	ND	ND	ND	ND	ND
hsa-miR-675-5p	ND	ND	ND	ND	ND
hsa-miR-708-3p	37.668818	36.8309248	36.158605	38.4800057	38.6951177
hsa-miR-7-1-3p	35.5372299	34.8236467	ND	30.5961066	32.4763687
hsa-miR-744-3p	39.1198159	ND	ND	37.8949894	37.40537
hsa-miR-744-5p	ND	37.0404101	ND	33.0694007	34.8841881
hsa-miR-758-3p	ND	ND	ND	ND	ND
hsa-miR-7-5p	33.7704203	31.5783283	33.787722	27.1824497	29.5206831
hsa-miR-760	ND	ND	ND	ND	ND
hsa-miR-765	ND	ND	ND	ND	ND
hsa-miR-766-3p	36.7807332	38.2479297	37.668426	36.5439699	35.0308048
hsa-miR-769-3p	ND	ND	ND	38.0841504	ND
hsa-miR-769-5p	35.0011897	36.2202073	ND	33.7745252	34.3080131
hsa-miR-770-5p	ND	36.6933683	ND	ND	38.1639262
hsa-miR-873-5p	ND	ND	ND	ND	ND
hsa-miR-874	34.5246962	35.0531524	34.822246	31.7705588	34.2868404
hsa-miR-876-3p	ND	ND	ND	ND	ND
hsa-miR-877-3p	ND	ND	ND	37.0328518	ND
hsa-miR-877-5p	34.6625588	34.6864732	34.742011	30.9891129	32.9278462
hsa-miR-885-5p	33.9410531	33.2398605	36.547928	34.1029636	33.8024355
hsa-miR-887	ND	ND	ND	ND	ND
hsa-miR-888-5p	ND	ND	ND	37.823989	ND
hsa-miR-889	ND	ND	ND	ND	ND
hsa-miR-92a-1-5p	ND	ND	ND	37.4759502	ND
hsa-miR-92a-3p	25.5139762	23.9083079	25.712719	20.5654083	22.6724467
hsa-miR-92b-3p	35.2738472	35.7506381	36.268607	31.9162091	34.5189396
hsa-miR-92b-5p	ND	ND	ND	35.6784181	ND
hsa-miR-93-3p	33.8123194	32.1421144	35.850776	27.7098733	29.3162969
hsa-miR-934	ND	36.0617349	37.410247	37.9307158	38.0537069
hsa-miR-93-5p	27.3228773	25.7214535	27.233599	21.685974	23.923654
hsa-miR-9-3p	36.9352193	ND	ND	ND	38.0944719
hsa-miR-940	ND	ND	ND	ND	ND
hsa-miR-941	ND	ND	ND	33.2212765	34.2045446
hsa-miR-942	37.2236031	36.8156994	36.623983	31.1193266	32.5316803
hsa-miR-95	ND	35.8247661	ND	35.7307826	37.9371932
hsa-miR-9-5p	ND	39.3532296	ND	36.8525636	ND
hsa-miR-96-5p	36.8462859	34.1231219	36.109102	29.3153149	31.6035823
hsa-miR-98-5p	35.7217697	34.5350884	35.936355	30.9134378	32.9558337
hsa-miR-99a-3p	ND	ND	ND	ND	ND
hsa-miR-99a-5p	32.9002579	32.3209466	34.021667	32.2427332	32.6229525
hsa-miR-99b-3p	ND	ND	ND	ND	37.1579556

hsa-miR-99b-5p	34.3655451	33.6385571	34.824065	33.6962387	34.2344655
SNORD38B	ND	ND	ND	ND	ND
SNORD49A	ND	ND	ND	ND	ND
UniSp2 CP	19.1883419	19.497562	20.547943	19.3381382	19.7800552
UniSp3 IPC	20.5740842	20.5853679	20.804992	20.7529959	20.7071197
UniSp3 IPC	19.9420745	19.946052	20.037583	20.067051	19.9409196
UniSp4 CP	25.651007	25.6520331	26.640484	25.631997	25.8886143
UniSp5 CP	30.9479659	30.9269047	32.145037	30.9413467	31.1727306
UniSp6 CP	19.8951432	19.0950461	19.569214	19.5281627	19.7100254

Table S1, continued

**Patient ID**	**2510**				
**Dose**	**0 Gy**	**8 Gy**	**28 Gy**	**42 Gy**	**54 Gy**
**Date**	**3/12/2014**	**3/24/2014**	**4/9/2014**	**4/21/2014**	**5/15/2014**
cel-miR-39-3p CP	ND	36.3303236	ND	ND	ND
hsa-let-7a-2-3p	ND	ND	ND	ND	ND
hsa-let-7a-3p	37.2696402	34.1792033	35.949578	36.8760315	34.885609
hsa-let-7a-5p	30.7606375	28.0944843	29.725964	29.1628108	29.0670514
hsa-let-7b-3p	34.9089857	32.0125546	32.818999	32.5952776	32.5630484
hsa-let-7b-5p	28.2323192	24.3471077	27.165379	26.0555577	25.8563993
hsa-let-7c	34.5813885	31.6924228	32.844817	33.0873553	32.78187
hsa-let-7d-3p	30.044377	27.6631717	29.55401	29.0464925	28.7697853
hsa-let-7d-5p	32.679343	30.0733102	31.804197	31.2349483	32.1119533
hsa-let-7e-3p	36.4439755	ND	ND	35.9177009	ND
hsa-let-7e-5p	35.9521676	33.5146459	35.011837	34.8973853	33.959569
hsa-let-7f-1-3p	ND	33.6608737	35.347204	35.328324	36.8099141
hsa-let-7f-2-3p	37.357775	37.1604094	38.389913	37.1672596	37.4352713
hsa-let-7f-5p	34.1112291	31.6734564	33.610039	33.005239	32.6712227
hsa-let-7g-3p	ND	34.0542617	35.015046	36.600612	34.3267112
hsa-let-7g-5p	29.1142338	25.6888537	28.182426	26.9007929	26.9813088
hsa-let-7i-3p	ND	35.4762878	35.69588	37.8377058	35.8261439
hsa-let-7i-5p	28.7770028	25.2330314	28.347112	27.1120783	26.9167161
hsa-miR-1	38.064274	35.1133443	37.511018	37.0741432	36.3179013
hsa-miR-100-5p	33.0628798	32.2937781	33.071466	32.9743268	33.3168804
hsa-miR-101-3p	28.4926851	24.55731	27.548483	26.2361491	25.8084678
hsa-miR-101-5p	36.4053671	34.0382788	36.819234	35.2976017	35.2348696
hsa-miR-103a-3p	29.3196939	26.0122215	28.489871	27.5963385	27.2832342
hsa-miR-105-3p	ND	ND	ND	ND	ND
hsa-miR-106a-3p	ND	ND	ND	ND	ND
hsa-miR-106a-5p	27.4803741	23.1589363	26.208753	25.0139502	24.7632458
hsa-miR-106b-3p	36.236471	34.5607987	35.229094	37.4890649	34.7649566
hsa-miR-106b-5p	28.8574849	24.891986	27.619592	26.5461358	26.0887864
hsa-miR-107	30.1957001	26.5276546	29.11499	28.3344382	28.0396496
hsa-miR-10a-5p	ND	37.9113079	ND	ND	ND
hsa-miR-10b-5p	32.5476814	31.5339533	31.835616	32.0404056	31.6922555
hsa-miR-1181	ND	ND	ND	ND	ND
hsa-miR-1183	ND	ND	ND	ND	ND
hsa-miR-1185-5p	ND	ND	ND	ND	ND
hsa-miR-1205	ND	ND	ND	ND	ND
hsa-miR-1207-5p	ND	ND	ND	ND	ND
hsa-miR-122-3p	ND	ND	36.097967	ND	36.6166827
hsa-miR-1224-3p	ND	ND	ND	39.2568006	ND
hsa-miR-122-5p	29.5530257	29.7779692	28.166384	28.0409187	27.833211

hsa-miR-1227-3p	ND	36.1080651	ND	ND	ND
hsa-miR-1237-3p	ND	ND	ND	37.2394227	ND
hsa-miR-1238-3p	ND	ND	ND	ND	ND
hsa-miR-1243	ND	ND	ND	ND	ND
hsa-miR-124-3p	ND	36.6785612	ND	ND	ND
hsa-miR-1245a	ND	38.4217106	ND	ND	ND
hsa-miR-1247-5p	ND	ND	ND	ND	ND
hsa-miR-1248	ND	ND	ND	ND	ND
hsa-miR-1249	ND	ND	36.182655	36.4462179	36.7617769
hsa-miR-1254	ND	ND	ND	ND	ND
hsa-miR-1255b-5p	ND	34.3119353	ND	36.2686244	ND
hsa-miR-1256	ND	ND	ND	ND	ND
hsa-miR-125a-3p	ND	ND	ND	ND	ND
hsa-miR-125a-5p	34.1902278	32.3611632	33.556006	33.1763249	32.8646804
hsa-miR-125b-2-3p	ND	ND	36.4023	ND	ND
hsa-miR-125b-5p	32.0514364	29.901608	31.675651	31.3031769	30.8316036
hsa-miR-1260a	34.2712375	ND	33.302255	35.5378312	33.7587149
hsa-miR-126-3p	28.6269818	26.0005273	27.885963	27.2954427	27.0905525
hsa-miR-1270	ND	35.0749061	ND	35.6077047	36.6949865
hsa-miR-1271-5p	37.3512788	35.2301205	ND	36.1427514	36.0950113
hsa-miR-127-3p	35.9345736	ND	ND	ND	36.9438323
hsa-miR-127-5p	ND	ND	ND	ND	ND
hsa-miR-128	31.7301535	28.2123486	31.596413	30.7207543	29.8559531
hsa-miR-129-5p	ND	ND	ND	ND	ND
hsa-miR-1296	ND	ND	ND	ND	ND
hsa-miR-130a-3p	32.0470623	28.0395762	30.775822	30.2891154	30.162994
hsa-miR-130b-3p	35.1360451	31.1814646	34.947171	32.7986997	32.752423
hsa-miR-130b-5p	37.205017	34.5670145	39.361647	37.4832183	35.1083155
hsa-miR-132-3p	33.6091454	31.7752405	34.235906	33.483458	32.7633454
hsa-miR-132-5p	ND	36.600755	ND	ND	ND
hsa-miR-133a	ND	34.5542094	36.301665	36.2386472	34.5098486
hsa-miR-133b	37.2426738	35.4753447	35.312173	37.5030554	35.1183777
hsa-miR-134	37.4529076	35.9839118	35.732626	38.0329899	37.4920314
hsa-miR-135a-3p	ND	ND	ND	ND	ND
hsa-miR-135a-5p	ND	37.7671782	ND	36.5924319	ND
hsa-miR-135b-5p	ND	37.4666767	ND	ND	ND
hsa-miR-136-3p	36.2721311	35.7712785	ND	ND	35.9433383
hsa-miR-136-5p	36.0368348	ND	ND	35.5822728	35.5096566
hsa-miR-139-3p	36.771442	33.4985205	ND	ND	ND
hsa-miR-139-5p	35.2973315	34.1392569	33.785625	34.2574857	34.192869
hsa-miR-140-3p	28.2612639	24.4973767	27.36508	26.4988469	26.1472552
hsa-miR-140-5p	32.2187084	28.7605849	32.514518	31.3255397	30.1760725
hsa-miR-141-3p	35.7566901	34.1677381	34.949391	33.9675215	34.2971743
hsa-miR-141-5p	ND	ND	ND	ND	ND

hsa-miR-142-3p	29.8282079	28.1780707	29.803371	29.7982521	28.9061998
hsa-miR-142-5p	32.6023535	29.8660414	32.004834	31.6297399	30.9081667
hsa-miR-143-3p	33.0293241	29.9635524	32.521505	32.5171646	30.4578263
hsa-miR-143-5p	ND	37.5888521	ND	ND	38.4767891
hsa-miR-144-3p	27.3358135	23.3580844	26.23533	25.1579109	24.2847485
hsa-miR-144-5p	32.183754	29.988684	32.054833	31.2711876	31.3045509
hsa-miR-145-3p	38.1781475	34.1112961	34.536361	35.2998451	33.8887218
hsa-miR-145-5p	32.325129	30.0226042	31.776924	31.7414224	30.5748399
hsa-miR-1468	ND	ND	39.231405	35.6873768	ND
hsa-miR-146a-5p	31.3631768	30.0296099	30.926926	30.6993087	30.5802549
hsa-miR-146b-3p	ND	ND	ND	ND	ND
hsa-miR-146b-5p	35.9911416	33.0118668	34.005344	34.6133156	33.9626388
hsa-miR-1471	ND	ND	ND	ND	ND
hsa-miR-147b	ND	ND	ND	ND	ND
hsa-miR-148a-3p	31.0186181	27.7602606	30.301183	29.2480947	28.6712059
hsa-miR-148b-3p	31.0676085	26.8785793	29.926402	28.8800427	28.4351332
hsa-miR-148b-5p	ND	37.3737648	ND	ND	ND
hsa-miR-149-5p	ND	35.8596703	ND	ND	ND
hsa-miR-150-5p	30.7244978	29.6368292	30.821053	31.0019416	31.5166209
hsa-miR-151a-3p	32.6611363	29.5169671	32.083544	31.6506611	30.9952246
hsa-miR-151a-5p	32.6795249	28.8031684	31.201351	30.4653169	30.5469507
hsa-miR-152	33.0566163	30.3381104	32.750126	31.9379422	31.5140607
hsa-miR-153	ND	36.8184428	ND	ND	36.7748241
hsa-miR-1537	36.9957142	33.4690217	36.635688	35.5087838	35.6950692
hsa-miR-1538	ND	ND	ND	37.1595529	ND
hsa-miR-154-5p	35.3021098	ND	35.855099	35.8678181	ND
hsa-miR-155-5p	36.9606669	35.2490224	36.645362	35.9354518	36.4862841
hsa-miR-15a-3p	ND	33.7836144	ND	35.7191011	34.8857147
hsa-miR-15a-5p	25.894546	22.013676	25.05853	23.8585638	23.641877
hsa-miR-15b-3p	31.0581348	27.6822825	30.591741	29.6422631	28.8965943
hsa-miR-15b-5p	30.0329027	26.1885215	28.978594	27.7815771	27.4285967
hsa-miR-16-1-3p	33.5836783	30.6233219	32.66415	32.3053003	31.7871206
hsa-miR-16-2-3p	30.9043509	27.8017536	30.614828	29.7216498	28.9190347
hsa-miR-16-5p	21.5392632	17.7185664	20.687734	19.4443451	19.481358
hsa-miR-17-3p	35.6834155	32.2510754	35.20596	34.8199757	33.6997471
hsa-miR-17-5p	33.042799	29.6730518	31.802804	31.6059297	31.2423978
hsa-miR-181a-2-3p	ND	ND	ND	ND	38.1840566
hsa-miR-181a-3p	ND	36.6528867	ND	ND	ND
hsa-miR-181a-5p	32.5045824	29.9511095	31.779232	31.6422221	31.8720913
hsa-miR-181b-5p	37.214791	34.5583338	35.208783	36.1317904	36.4259632
hsa-miR-181c-3p	ND	ND	ND	ND	35.0875905
hsa-miR-181c-5p	35.6906604	34.4990644	ND	ND	36.66972
hsa-miR-181d	ND	ND	ND	ND	ND
hsa-miR-182-3p	36.4239878	ND	ND	ND	ND

hsa-miR-182-5p	37.3239651	35.0075237	36.408528	36.8978985	36.163182
hsa-miR-183-3p	ND	35.0225227	36.892118	36.9552741	36.7719891
hsa-miR-183-5p	ND	31.8515764	34.139431	33.8446048	33.497685
hsa-miR-184	ND	37.2055144	37.582756	ND	ND
hsa-miR-185-3p	ND	35.6957281	ND	ND	ND
hsa-miR-185-5p	28.1279905	23.6158378	27.039654	26.272812	25.3460532
hsa-miR-186-5p	29.7425552	25.6684326	28.868842	27.7493756	27.2063638
hsa-miR-187-3p	ND	ND	ND	ND	ND
hsa-miR-187-5p	ND	ND	ND	37.1174753	ND
hsa-miR-188-3p	38.4390024	35.9792271	ND	37.9906517	ND
hsa-miR-188-5p	36.6291564	32.5686262	37.160716	35.0180172	34.8587527
hsa-miR-18a-3p	35.2608962	29.9181492	34.729125	31.7873747	31.7983905
hsa-miR-18a-5p	31.5071964	26.8468067	29.620099	28.6946202	28.2862518
hsa-miR-18b-3p	ND	ND	ND	ND	ND
hsa-miR-18b-5p	30.6642162	26.5199375	29.330393	28.2887569	27.8428916
hsa-miR-1908	ND	35.9249203	36.77249	38.0910299	35.7997707
hsa-miR-1909-3p	37.2147042	ND	ND	ND	ND
hsa-miR-190a	37.3734128	37.727119	ND	38.552306	37.446891
hsa-miR-190b	39.2351077	35.4452405	ND	ND	37.8411047
hsa-miR-1912	ND	ND	ND	ND	ND
hsa-miR-1913	ND	ND	ND	ND	38.2082099
hsa-miR-191-3p	37.1592537	35.8189851	ND	ND	38.2896578
hsa-miR-1914-5p	ND	ND	ND	ND	ND
hsa-miR-191-5p	31.7808029	27.750605	30.164621	28.9686572	28.8766092
hsa-miR-192-3p	ND	ND	ND	37.2829676	35.1431574
hsa-miR-192-5p	30.9617783	27.0777789	29.499486	29.1208001	28.7366801
hsa-miR-193a-3p	ND	38.7162774	ND	ND	38.1746589
hsa-miR-193a-5p	37.0373063	33.2169035	34.274517	33.8855269	34.5895176
hsa-miR-193b-3p	33.6191831	34.1272689	35.194817	34.8967239	34.0442839
hsa-miR-193b-5p	ND	ND	ND	ND	36.1773194
hsa-miR-194-3p	ND	ND	ND	ND	ND
hsa-miR-194-5p	32.2868676	28.7266224	31.381981	30.5327907	30.537861
hsa-miR-195-5p	33.9779326	34.0417553	34.324127	33.9115802	33.2158932
hsa-miR-196a-5p	ND	ND	ND	ND	ND
hsa-miR-196b-3p	ND	34.3953576	ND	ND	35.9963743
hsa-miR-196b-5p	38.046694	33.6672363	36.481173	35.0869434	35.4405713
hsa-miR-1972	35.4844076	36.5066861	36.156823	38.4455734	36.8303029
hsa-miR-197-3p	32.7822344	32.6253389	32.657981	32.0493946	32.1812594
hsa-miR-199a-3p	32.5039185	30.4617825	31.591311	31.8292809	30.8356246
hsa-miR-199a-5p	34.8930439	32.9823741	35.710442	35.6375416	32.7452904
hsa-miR-199b-5p	36.7557213	35.8752068	ND	35.9638233	34.7338922
hsa-miR-19a-3p	25.8343833	21.9318273	25.143668	23.9275611	23.173199
hsa-miR-19a-5p	38.4545597	36.038163	ND	38.0395458	37.5720567
hsa-miR-19b-1-5p	38.5715951	38.8215502	ND	38.533711	38.328562

hsa-miR-19b-3p	25.1102121	21.1327962	24.329571	23.1063454	22.6304257
hsa-miR-200a-3p	37.1720242	35.2841092	34.987678	35.2326005	35.5250361
hsa-miR-200b-3p	37.8843501	ND	ND	ND	ND
hsa-miR-200b-5p	ND	ND	ND	ND	ND
hsa-miR-200c-3p	35.270308	34.2995756	34.6579	34.7465662	36.0892124
hsa-miR-200c-5p	ND	ND	ND	ND	ND
hsa-miR-202-3p	ND	ND	ND	ND	ND
hsa-miR-202-5p	36.5089504	ND	ND	ND	ND
hsa-miR-203a	35.8422215	36.1612791	ND	ND	ND
hsa-miR-204-5p	37.3670282	34.6824187	37.000765	35.5726826	ND
hsa-miR-205-5p	34.1846213	33.6470577	33.25447	32.7900375	32.7515297
hsa-miR-206	36.5768719	34.0380494	34.850297	36.9580713	35.2982372
hsa-miR-208b	ND	ND	ND	ND	ND
hsa-miR-20a-3p	ND	33.6043754	36.137107	35.6787238	35.123781
hsa-miR-20a-5p	26.1997503	22.3626104	25.295506	24.0603416	23.8422807
hsa-miR-20b-3p	37.9588963	ND	ND	36.5650234	36.9483893
hsa-miR-20b-5p	35.781559	31.037819	33.65552	32.6275543	32.1240328
hsa-miR-210	31.9144217	27.8578041	31.496196	29.881759	29.3654299
hsa-miR-2110	35.9293672	31.0694892	35.215302	33.1289877	32.6516295
hsa-miR-211-5p	ND	35.2703095	ND	ND	ND
hsa-miR-212-3p	ND	ND	ND	ND	ND
hsa-miR-212-5p	ND	ND	ND	ND	ND
hsa-miR-21-3p	37.7835561	35.8182578	38.050053	ND	36.4923957
hsa-miR-214-3p	37.9373557	34.151265	35.810081	36.7297718	35.5118053
hsa-miR-214-5p	ND	ND	ND	35.5224011	34.9729533
hsa-miR-215	31.7108177	28.0225551	30.802278	29.0950176	29.2588489
hsa-miR-21-5p	25.7206471	22.8308916	24.995633	24.1883615	23.9948168
hsa-miR-216a-5p	ND	ND	ND	ND	ND
hsa-miR-217	ND	ND	ND	ND	ND
hsa-miR-218-2-3p	ND	ND	ND	ND	ND
hsa-miR-218-5p	ND	ND	ND	ND	ND
hsa-miR-219-1-3p	ND	ND	ND	36.9214189	ND
hsa-miR-219-5p	ND	35.7539454	36.653389	36.5323365	ND
hsa-miR-221-3p	30.976841	29.8951729	30.842312	30.8657974	29.430968
hsa-miR-221-5p	39.2752915	35.0670058	ND	ND	ND
hsa-miR-222-3p	29.0287299	25.8278604	28.718686	27.8826792	27.8331756
hsa-miR-223-3p	26.0255404	24.2855835	25.730175	25.1318601	25.0206945
hsa-miR-223-5p	34.5951392	34.0916906	36.585682	35.2706851	35.5028193
hsa-miR-22-3p	29.3717155	25.7249627	28.6304	27.6784781	27.3362299
hsa-miR-224-3p	ND	36.5108564	ND	ND	36.6512729
hsa-miR-224-5p	37.8529659	37.2216898	36.805572	35.7402063	37.2742445
hsa-miR-22-5p	32.5251605	29.8913113	32.667334	32.0118599	31.263579
hsa-miR-23a-3p	27.7084075	26.4632379	27.436114	27.4713252	26.8443914
hsa-miR-23a-5p	ND	ND	ND	ND	ND

hsa-miR-23b-3p	30.7356855	29.6574114	30.335329	30.5309011	29.6885384
hsa-miR-23b-5p	ND	ND	ND	ND	ND
hsa-miR-24-1-5p	ND	ND	ND	ND	ND
hsa-miR-24-2-5p	ND	ND	34.667916	37.2450816	ND
hsa-miR-24-3p	28.4918288	26.3391238	27.941821	27.9097365	27.2894906
hsa-miR-25-3p	26.760551	22.9595329	26.335629	24.9541031	24.8478141
hsa-miR-25-5p	ND	33.3185117	ND	35.8858781	34.9351342
hsa-miR-26a-1-3p	ND	ND	35.790428	ND	ND
hsa-miR-26a-2-3p	ND	ND	ND	ND	ND
hsa-miR-26a-5p	31.5170001	28.2603873	29.995059	29.5948403	29.7199706
hsa-miR-26b-3p	ND	35.6928769	36.622237	37.8289523	35.1022131
hsa-miR-26b-5p	31.6668152	27.7492964	29.937598	29.5297808	29.1950239
hsa-miR-27a-3p	31.9073031	ND	30.702466	31.2529	ND
hsa-miR-27a-5p	36.0221872	37.4316368	37.746934	36.0935785	36.2598317
hsa-miR-27b-3p	30.7553617	28.7327629	30.121262	30.1629342	29.0926124
hsa-miR-27b-5p	ND	ND	ND	ND	ND
hsa-miR-28-3p	35.8303554	33.1740606	33.525164	35.8195757	33.5366861
hsa-miR-28-5p	36.7158715	35.8358415	34.92805	35.7079879	35.5165611
hsa-miR-296-3p	ND	ND	ND	ND	ND
hsa-miR-296-5p	35.077545	30.9632177	34.259279	32.1395242	32.6823065
hsa-miR-299-3p	ND	ND	ND	ND	ND
hsa-miR-299-5p	ND	35.9425108	35.927055	ND	ND
hsa-miR-29a-3p	31.2086893	29.643422	31.460911	30.5572399	30.2209328
hsa-miR-29a-5p	36.6790427	39.2901376	ND	ND	ND
hsa-miR-29b-2-5p	36.0409284	32.7330192	35.601878	34.033492	33.7701488
hsa-miR-29b-3p	31.2744008	27.777319	30.837084	30.0478425	29.456451
hsa-miR-29c-3p	28.9891566	25.7607719	28.671955	27.8704584	27.2109857
hsa-miR-29c-5p	37.2633535	34.3745333	ND	34.8416782	35.1723048
hsa-miR-300	ND	ND	38.334121	ND	ND
hsa-miR-301a-3p	33.4907242	29.8050271	31.950842	30.9306673	30.4468407
hsa-miR-301b	ND	37.516102	ND	36.0041603	36.4807706
hsa-miR-302c-5p	ND	ND	ND	36.5256622	ND
hsa-miR-302d-5p	ND	36.762391	ND	ND	ND
hsa-miR-30a-3p	37.2345258	36.0381853	35.902364	39.4071877	35.63475
hsa-miR-30a-5p	33.7695439	32.83205	32.572045	31.929997	32.0862728
hsa-miR-30b-5p	31.9800666	29.0976488	31.126461	31.0865072	30.6528222
hsa-miR-30c-2-3p	ND	ND	ND	ND	ND
hsa-miR-30c-5p	32.5445059	29.4508427	31.868025	31.1857798	30.6436311
hsa-miR-30d-3p	ND	ND	ND	ND	ND
hsa-miR-30d-5p	34.2658985	31.0900184	33.047901	32.5916769	31.8930646
hsa-miR-30e-3p	36.7649197	33.7794109	35.164975	34.8201432	35.7252957
hsa-miR-30e-5p	28.7016467	25.5091818	28.021455	27.1399496	26.9066637
hsa-miR-31-3p	ND	ND	ND	ND	ND
hsa-miR-31-5p	ND	ND	ND	ND	ND

hsa-miR-320a	28.3840441	25.143689	27.884329	26.7096703	26.3782072
hsa-miR-320b	30.1808507	26.8251245	29.77473	28.6763575	28.227417
hsa-miR-320c	30.6057202	27.7896713	30.738366	29.70658	29.5843384
hsa-miR-320d	29.8831962	26.9861691	29.952524	28.7767067	28.6644438
hsa-miR-323a-3p	36.9364501	35.1450689	ND	ND	ND
hsa-miR-32-3p	ND	37.2671855	ND	ND	ND
hsa-miR-324-3p	30.8767068	27.507212	30.768685	29.6606447	29.4731406
hsa-miR-324-5p	34.11543	29.8285724	32.667331	32.2500299	31.1240353
hsa-miR-32-5p	31.8274249	28.2460244	31.230258	30.1619692	29.4803136
hsa-miR-326	ND	36.8456129	35.596719	35.9837417	35.2385
hsa-miR-328	34.7482225	31.3063357	34.455267	33.5083095	32.2140185
hsa-miR-329	ND	38.9536263	35.94687	35.8422192	36.7654536
hsa-miR-330-3p	ND	ND	ND	36.1579684	ND
hsa-miR-330-5p	ND	ND	ND	ND	ND
hsa-miR-331-3p	36.4458423	33.5895653	35.285603	34.2295284	34.3279909
hsa-miR-331-5p	ND	ND	ND	ND	ND
hsa-miR-335-3p	35.8179706	35.0972678	ND	ND	34.728758
hsa-miR-335-5p	34.3694189	30.9221164	32.823335	32.5184276	32.9852179
hsa-miR-337-3p	35.8699825	37.0591985	ND	ND	35.8034061
hsa-miR-337-5p	ND	37.6677053	37.427696	37.4441497	ND
hsa-miR-338-3p	37.6177962	34.2168907	35.024792	34.2043939	34.1887538
hsa-miR-338-5p	ND	37.0780929	ND	ND	ND
hsa-miR-339-3p	35.3350965	32.066034	33.690199	33.8633492	32.8854892
hsa-miR-339-5p	ND	37.6056026	ND	ND	35.6236622
hsa-miR-33a-3p	ND	ND	ND	ND	39.0614187
hsa-miR-33a-5p	36.0850107	37.2409366	36.627659	38.4648531	34.8414991
hsa-miR-33b-3p	ND	ND	ND	ND	ND
hsa-miR-33b-5p	36.1887415	32.3505613	35.503662	34.0814475	33.2646725
hsa-miR-340-3p	ND	38.8136909	ND	ND	ND
hsa-miR-340-5p	ND	ND	ND	ND	38.2590716
hsa-miR-342-3p	31.9665357	29.6050208	30.882904	30.885769	30.7251835
hsa-miR-342-5p	37.1671454	33.849995	36.596485	35.5643085	34.9008438
hsa-miR-345-5p	37.9588523	ND	ND	38.3295448	37.1101857
hsa-miR-346	ND	ND	ND	37.076831	ND
hsa-miR-34a-3p	36.4344177	36.9668887	38.217574	37.1004151	34.9231278
hsa-miR-34a-5p	33.4844267	32.8645513	31.923225	31.723688	31.3764419
hsa-miR-34b-3p	ND	ND	ND	ND	ND
hsa-miR-34c-5p	ND	ND	ND	ND	37.6769244
hsa-miR-361-3p	ND	32.4115409	35.04961	35.6998162	33.6587683
hsa-miR-361-5p	31.9498634	29.7967829	31.435052	31.4872899	30.8171511
hsa-miR-362-3p	33.1623425	30.585081	32.9016	32.6281014	31.6432847
hsa-miR-362-5p	37.7196608	37.0169253	38.13761	ND	36.8198885
hsa-miR-363-3p	29.8227345	26.0410674	29.314937	28.0299799	27.784859
hsa-miR-363-5p	ND	38.2624847	ND	ND	ND

hsa-miR-365a-3p	34.1324493	32.0256267	33.254956	34.5603964	32.9457936
hsa-miR-369-3p	ND	38.2574012	ND	ND	ND
hsa-miR-369-5p	ND	38.4545232	ND	ND	ND
hsa-miR-370	ND	ND	ND	ND	ND
hsa-miR-373-3p	37.1743188	ND	ND	ND	ND
hsa-miR-373-5p	ND	38.2697372	38.17975	38.3662885	39.177761
hsa-miR-374a-5p	35.4778622	31.9348142	33.766043	32.7321498	32.9614991
hsa-miR-374b-3p	39.1528023	37.1852538	35.740356	ND	37.1229476
hsa-miR-374b-5p	37.3570175	32.8673078	34.242189	33.9822051	33.5856702
hsa-miR-375	32.8485286	32.1754302	33.08853	33.1135794	33.0776529
hsa-miR-376a-3p	36.9093446	35.8348899	ND	37.6723651	37.2429529
hsa-miR-376a-5p	ND	ND	ND	ND	ND
hsa-miR-376b-3p	ND	ND	35.966374	ND	ND
hsa-miR-376c-3p	35.9851575	34.0908763	36.150719	36.2751394	34.6666812
hsa-miR-377-3p	ND	39.258021	ND	38.5657075	37.720832
hsa-miR-377-5p	ND	ND	ND	ND	ND
hsa-miR-378a-3p	30.8587862	27.8314385	30.037653	29.633074	28.948758
hsa-miR-378a-5p	37.2165695	32.9074993	34.551445	34.2910042	35.5325039
hsa-miR-379-3p	ND	ND	ND	ND	ND
hsa-miR-379-5p	ND	ND	ND	ND	ND
hsa-miR-380-3p	ND	ND	37.860282	ND	ND
hsa-miR-381-3p	ND	36.976015	ND	ND	ND
hsa-miR-382-3p	ND	ND	ND	ND	ND
hsa-miR-382-5p	36.6756277	34.5782988	ND	36.5310193	ND
hsa-miR-409-3p	35.1028544	36.2134846	33.960855	35.715909	ND
hsa-miR-409-5p	ND	ND	ND	ND	ND
hsa-miR-410	ND	ND	36.753008	ND	36.4588298
hsa-miR-411-5p	ND	ND	ND	ND	ND
hsa-miR-412	ND	ND	ND	36.5940862	ND
hsa-miR-421	35.6481445	32.8175023	36.834311	34.2144578	33.7101808
hsa-miR-423-3p	32.2554352	27.9761971	31.128262	30.1970046	29.4822457
hsa-miR-423-5p	29.8366222	26.4657448	29.818616	28.2185896	28.0042381
hsa-miR-424-3p	35.9248201	32.908585	36.314714	34.7946323	34.7672431
hsa-miR-424-5p	31.6892037	28.6137263	30.697858	30.4421776	29.320847
hsa-miR-425-3p	34.4673144	30.624918	33.089311	32.4744621	32.471821
hsa-miR-425-5p	28.8782449	25.5662253	28.664919	27.5847014	27.2106961
hsa-miR-429	ND	ND	ND	35.6136961	ND
hsa-miR-431-3p	ND	38.3622919	ND	ND	ND
hsa-miR-431-5p	ND	37.34267	ND	ND	ND
hsa-miR-432-3p	ND	ND	ND	ND	ND
hsa-miR-432-5p	ND	ND	ND	ND	ND
hsa-miR-433	ND	ND	ND	ND	ND
hsa-miR-449a	ND	34.7976105	38.334466	36.4748756	35.3680263
hsa-miR-449b-5p	ND	ND	ND	ND	ND

hsa-miR-450a-5p	39.5110076	37.2078237	ND	ND	ND
hsa-miR-450b-3p	ND	ND	ND	ND	ND
hsa-miR-450b-5p	36.4505709	35.7509202	ND	36.5960129	ND
hsa-miR-451a	20.4789394	16.9436961	20.04685	18.8672928	18.7349214
hsa-miR-452-5p	36.815484	ND	ND	36.9957827	36.8384752
hsa-miR-454-3p	32.8386857	30.1602316	32.011416	30.9512996	31.6803337
hsa-miR-454-5p	ND	37.7757592	ND	ND	ND
hsa-miR-455-3p	ND	ND	ND	36.1825983	ND
hsa-miR-455-5p	ND	35.8543576	36.876407	ND	ND
hsa-miR-483-3p	ND	37.7021558	39.558596	ND	37.4716285
hsa-miR-483-5p	38.5288594	36.123381	36.029221	35.6646019	36.6230365
hsa-miR-484	29.8913737	25.9454966	28.981723	27.9497706	27.6139549
hsa-miR-486-3p	34.2827031	30.0125628	33.08871	32.1326475	31.7332143
hsa-miR-486-5p	25.9844275	22.0021834	25.017999	23.9080822	23.5925217
hsa-miR-487a	ND	ND	ND	ND	ND
hsa-miR-487b	ND	ND	37.001305	ND	37.0978202
hsa-miR-489	ND	ND	ND	ND	ND
hsa-miR-490-3p	34.1584316	33.3395023	32.445371	33.3635464	33.6856572
hsa-miR-490-5p	ND	ND	ND	ND	38.3634898
hsa-miR-491-5p	ND	34.8142664	ND	33.9535094	36.0566098
hsa-miR-493-3p	ND	ND	ND	ND	ND
hsa-miR-493-5p	ND	ND	ND	ND	ND
hsa-miR-494	ND	ND	ND	ND	ND
hsa-miR-495-3p	36.6752832	35.3390821	36.284214	35.0271802	35.8665034
hsa-miR-496	ND	ND	ND	ND	ND
hsa-miR-497-5p	33.2125501	32.4632758	33.99018	34.1195802	32.80255
hsa-miR-499a-5p	ND	34.6069129	ND	ND	ND
hsa-miR-500a-5p	35.7896945	33.6162735	35.974363	35.9657113	34.9434487
hsa-miR-501-3p	34.5472023	32.5694927	34.161179	34.6661253	32.9564426
hsa-miR-501-5p	34.3396069	32.3013709	34.853296	35.8830565	33.0280603
hsa-miR-502-3p	33.8115351	29.9621707	33.024535	31.704331	31.5386465
hsa-miR-502-5p	36.182391	33.0861992	35.659108	ND	34.8100478
hsa-miR-503-5p	ND	36.7679139	ND	ND	ND
hsa-miR-505-3p	33.8171148	32.3550877	34.053026	32.9593473	32.0598604
hsa-miR-505-5p	36.1442795	33.1296539	37.980173	35.0225961	37.0918541
hsa-miR-507	ND	ND	ND	ND	ND
hsa-miR-508-3p	ND	ND	ND	38.3425948	ND
hsa-miR-509-3-5p	ND	ND	ND	ND	ND
hsa-miR-509-3p	ND	ND	ND	35.0657368	ND
hsa-miR-511	37.0613734	36.7634995	ND	35.7950966	ND
hsa-miR-513a-5p	ND	ND	ND	ND	ND
hsa-miR-514a-3p	ND	ND	ND	ND	ND
hsa-miR-515-3p	ND	36.9430493	36.990649	ND	ND
hsa-miR-517c-3p	ND	ND	ND	ND	ND

hsa-miR-518d-3p	ND	ND	ND	ND	ND
hsa-miR-518f-3p	ND	ND	ND	ND	ND
hsa-miR-518f-5p	ND	ND	ND	ND	ND
hsa-miR-519b-3p	ND	ND	ND	ND	ND
hsa-miR-520a-5p	ND	ND	ND	ND	ND
hsa-miR-520c-3p	ND	ND	ND	37.7691571	ND
hsa-miR-520g	ND	ND	ND	ND	ND
hsa-miR-520h	36.8692909	37.7446281	37.052645	37.3796738	36.6019311
hsa-miR-524-3p	ND	ND	ND	ND	ND
hsa-miR-525-3p	ND	ND	ND	ND	ND
hsa-miR-525-5p	ND	ND	ND	ND	ND
hsa-miR-532-3p	33.8375809	30.3709005	32.576988	31.9963331	32.0763381
hsa-miR-532-5p	31.8659665	28.3211067	30.806684	30.3395978	29.9822477
hsa-miR-539-5p	ND	ND	ND	ND	35.9943718
hsa-miR-542-5p	ND	35.7539608	ND	36.013889	ND
hsa-miR-543	36.137218	ND	37.000864	ND	ND
hsa-miR-544a	ND	ND	ND	ND	ND
hsa-miR-545-3p	38.9830327	35.7341288	37.612457	36.5177208	35.4973481
hsa-miR-548a-3p	38.099556	38.7273424	35.324821	36.9548208	35.8910319
hsa-miR-548a-5p	ND	ND	ND	ND	ND
hsa-miR-548b-3p	ND	ND	ND	ND	ND
hsa-miR-548c-5p	39.4078492	36.7929263	37.595532	38.0190795	39.2066469
hsa-miR-548d-3p	ND	39.3659685	ND	ND	ND
hsa-miR-548d-5p	ND	ND	ND	ND	ND
hsa-miR-548e	ND	ND	ND	ND	ND
hsa-miR-548j	ND	35.021403	35.839911	36.2851728	36.0327752
hsa-miR-548k	ND	ND	ND	36.2589043	36.8778477
hsa-miR-548l	38.2896319	36.1682781	ND	ND	38.1061869
hsa-miR-548n	ND	ND	ND	ND	ND
hsa-miR-549a	ND	ND	ND	ND	ND
hsa-miR-550a-3p	35.194183	30.6666648	33.893055	32.4731702	32.523532
hsa-miR-550a-5p	ND	34.7847428	ND	35.2382442	35.3709885
hsa-miR-551a	35.9377891	34.9271893	37.43756	ND	36.6931594
hsa-miR-551b-3p	ND	ND	ND	36.190598	ND
hsa-miR-551b-5p	ND	ND	ND	ND	ND
hsa-miR-556-3p	ND	ND	ND	ND	ND
hsa-miR-564	ND	ND	ND	ND	ND
hsa-miR-570-3p	36.4325178	33.960965	ND	36.9054515	35.2001845
hsa-miR-571	ND	ND	ND	ND	ND
hsa-miR-573	ND	ND	ND	ND	ND
hsa-miR-574-3p	32.794476	30.9487822	32.29979	32.0154837	31.9536514
hsa-miR-576-3p	38.1537423	35.1649378	ND	38.9007181	36.4896724
hsa-miR-576-5p	39.4465934	ND	ND	ND	ND
hsa-miR-579	36.1949626	33.8070562	36.141921	36.7537703	34.6209091

hsa-miR-580	ND	ND	ND	ND	ND
hsa-miR-581	ND	ND	36.774888	ND	ND
hsa-miR-582-5p	37.1600276	35.2583697	37.170897	34.4666416	35.0404302
hsa-miR-584-5p	36.07603	31.794892	33.926976	33.5412829	32.7235909
hsa-miR-589-3p	ND	ND	ND	37.2370388	ND
hsa-miR-589-5p	38.4321	36.0658982	ND	ND	36.5244104
hsa-miR-590-3p	38.0121642	34.0054955	ND	36.9475422	35.9859548
hsa-miR-590-5p	32.0179421	28.7385938	31.76122	30.6151193	29.9726678
hsa-miR-596	ND	ND	ND	ND	ND
hsa-miR-597	ND	ND	ND	ND	ND
hsa-miR-598	35.3376266	32.3635107	34.224256	35.0692869	33.5819547
hsa-miR-601	ND	ND	ND	37.7432133	ND
hsa-miR-604	ND	ND	ND	ND	ND
hsa-miR-605	ND	ND	ND	ND	ND
hsa-miR-610	ND	35.9768515	36.427602	ND	38.2482247
hsa-miR-612	ND	ND	ND	ND	ND
hsa-miR-615-3p	37.7310733	ND	ND	ND	ND
hsa-miR-616-5p	ND	35.4952401	ND	ND	36.1576251
hsa-miR-618	ND	ND	ND	ND	ND
hsa-miR-621	ND	ND	ND	ND	ND
hsa-miR-624-5p	32.2480674	30.2687605	32.005435	31.5542074	31.8001428
hsa-miR-625-3p	38.0115113	35.0521904	37.222906	35.65283	34.4417681
hsa-miR-626	ND	ND	ND	ND	39.1605547
hsa-miR-627	38.0317019	34.1562405	ND	36.5634149	35.1394744
hsa-miR-628-3p	35.8117218	33.8475368	34.879173	35.8162586	33.9184262
hsa-miR-628-5p	ND	36.1244262	ND	ND	37.8033713
hsa-miR-629-3p	ND	36.8128602	36.676203	36.0824532	ND
hsa-miR-629-5p	34.0616311	29.3038974	32.577693	31.020306	30.8248934
hsa-miR-636	38.046791	34.1951569	ND	35.0669988	34.9209018
hsa-miR-641	ND	ND	ND	ND	ND
hsa-miR-642a-5p	ND	ND	ND	ND	ND
hsa-miR-643	ND	ND	ND	ND	ND
hsa-miR-650	ND	ND	ND	ND	ND
hsa-miR-651	ND	35.2867437	35.827558	36.4993229	35.2068607
hsa-miR-652-3p	31.5102241	27.8790527	30.437104	29.6403812	29.5166883
hsa-miR-654-3p	ND	38.1740611	ND	ND	ND
hsa-miR-654-5p	ND	ND	ND	ND	ND
hsa-miR-655	36.7677777	37.6343585	36.750669	37.2097279	37.7264005
hsa-miR-659-3p	ND	ND	36.593328	ND	ND
hsa-miR-660-5p	30.2325726	26.9999335	30.241749	28.9948345	28.4481071
hsa-miR-662	ND	ND	ND	ND	ND
hsa-miR-663a	ND	37.7142211	34.882006	35.173947	36.2281474
hsa-miR-664a-3p	38.4556108	34.944541	ND	ND	36.9973311
hsa-miR-665	36.0538821	ND	ND	ND	ND

hsa-miR-668	ND	ND	ND	ND	ND
hsa-miR-671-3p	ND	ND	ND	ND	ND
hsa-miR-671-5p	ND	ND	ND	ND	ND
hsa-miR-675-3p	ND	ND	ND	38.9398285	ND
hsa-miR-675-5p	ND	ND	ND	35.6508944	ND
hsa-miR-708-3p	ND	ND	ND	36.078484	ND
hsa-miR-7-1-3p	34.8138479	31.6758644	36.216157	33.018115	32.5414649
hsa-miR-744-3p	37.4028657	ND	36.952724	35.4364983	ND
hsa-miR-744-5p	36.9777875	34.1084036	36.55137	36.4916371	35.5825796
hsa-miR-758-3p	ND	ND	ND	ND	ND
hsa-miR-7-5p	33.8887488	30.1606785	32.607817	30.7663882	31.0867221
hsa-miR-760	ND	36.2168405	ND	ND	ND
hsa-miR-765	ND	ND	ND	ND	ND
hsa-miR-766-3p	38.1884696	34.180554	ND	34.8651409	34.5692363
hsa-miR-769-3p	ND	38.4071281	38.053663	ND	ND
hsa-miR-769-5p	ND	34.7711217	36.412399	ND	36.626804
hsa-miR-770-5p	ND	ND	ND	ND	37.4155157
hsa-miR-873-5p	ND	ND	ND	ND	ND
hsa-miR-874	34.039581	32.2187073	35.922871	33.5944641	33.6153208
hsa-miR-876-3p	ND	39.0920683	ND	ND	ND
hsa-miR-877-3p	ND	ND	ND	ND	39.2909379
hsa-miR-877-5p	ND	31.9136494	34.593695	33.3387336	32.9567513
hsa-miR-885-5p	34.2022246	34.9008385	34.437954	34.2887355	33.1817358
hsa-miR-887	ND	ND	37.844911	37.0947338	39.177322
hsa-miR-888-5p	ND	ND	ND	ND	ND
hsa-miR-889	ND	ND	ND	38.4327615	ND
hsa-miR-92a-1-5p	ND	ND	ND	ND	ND
hsa-miR-92a-3p	25.5578862	21.7866646	24.717512	23.6023423	23.527831
hsa-miR-92b-3p	36.1537444	33.6649623	35.991132	35.8697726	34.8585544
hsa-miR-92b-5p	ND	37.9354325	ND	36.2599236	ND
hsa-miR-93-3p	35.2720068	29.0885359	32.536826	32.2894918	30.5968973
hsa-miR-934	37.9199092	ND	ND	ND	36.7528795
hsa-miR-93-5p	27.1062864	23.1434066	26.137852	24.910784	24.7005851
hsa-miR-9-3p	ND	ND	ND	ND	38.2395146
hsa-miR-940	38.0713508	ND	ND	ND	ND
hsa-miR-941	36.0444383	33.57644	35.795713	35.198213	37.6425198
hsa-miR-942	35.6742844	32.0926614	36.526663	33.9557703	33.8500507
hsa-miR-95	ND	36.5231619	35.835001	ND	37.4086808
hsa-miR-9-5p	ND	ND	ND	ND	ND
hsa-miR-96-5p	35.8278759	31.7088474	33.759524	33.6531408	33.0119172
hsa-miR-98-5p	36.076019	32.6327703	35.64609	33.8637161	34.4873762
hsa-miR-99a-3p	36.1136077	ND	ND	ND	ND
hsa-miR-99a-5p	33.8988227	31.9894288	32.656211	32.6871199	32.3934602
hsa-miR-99b-3p	ND	ND	ND	ND	ND

hsa-miR-99b-5p	35.3375409	32.7237165	33.763465	33.1197474	33.0953857
SNORD38B	39.4870151	36.4440251	34.758752	ND	ND
SNORD49A	ND	ND	35.744337	ND	35.9728025
UniSp2 CP	19.8515677	19.275499	19.173087	19.2091009	19.1684288
UniSp3 IPC	20.9603692	20.7764754	20.543942	20.6594594	20.8123122
UniSp3 IPC	20.2622572	20.0938517	19.826115	19.7517138	20.110087
UniSp4 CP	26.0491721	25.5797581	25.56702	25.5718265	25.6176788
UniSp5 CP	31.4243919	31.4291799	30.844056	30.7781973	31.2538173
UniSp6 CP	20.165938	19.5170676	19.263264	19.6348601	19.4958506

Table S1, continued

**Patient ID**	**2526**				
**Dose**	**0 Gy**	**10 Gy**	**20 Gy**	**30 Gy**	**46 Gy**
**Date**	**4/7/2014**	**4/15/2014**	**4/24/2014**	**5/1/2014**	**5/13/2014**
cel-miR-39-3p CP	36.206445	35.9908096	36.440296	35.12087	ND
hsa-let-7a-2-3p	ND	ND	ND	ND	ND
hsa-let-7a-3p	38.423296	37.2066755	36.2162128	36.862444	37.3005674
hsa-let-7a-5p	29.948526	29.969233	25.9084799	29.728297	30.5323789
hsa-let-7b-3p	33.346986	38.1160205	32.3141144	33.612315	33.7539984
hsa-let-7b-5p	27.723541	26.6540108	27.1479001	26.90301	26.9317548
hsa-let-7c	34.036056	34.1642529	30.3358054	33.726838	33.7339893
hsa-let-7d-3p	30.023675	29.4714637	26.2924582	29.849078	29.8811217
hsa-let-7d-5p	33.051374	31.9083874	28.1988097	31.899202	32.9612646
hsa-let-7e-3p	ND	ND	34.8215998	ND	ND
hsa-let-7e-5p	35.431927	36.273489	30.9414385	34.737586	36.5653613
hsa-let-7f-1-3p	ND	36.0025242	33.0156137	36.729879	36.3034444
hsa-let-7f-2-3p	39.215693	ND	35.546917	37.46572	38.1841781
hsa-let-7f-5p	33.526397	33.7810715	28.1711311	33.866897	34.2585595
hsa-let-7g-3p	ND	ND	34.0826936	ND	ND
hsa-let-7g-5p	28.557175	27.9598851	26.2266892	27.790986	28.2387283
hsa-let-7i-3p	38.472452	36.3146918	35.864817	36.850969	36.4768107
hsa-let-7i-5p	28.351685	27.0289044	26.3721472	27.628174	27.6295839
hsa-miR-1	36.259949	ND	33.2054244	37.472816	35.6300874
hsa-miR-100-5p	33.443773	33.7997814	33.8776948	32.717747	32.7907365
hsa-miR-101-3p	27.976219	26.6946881	26.4911661	27.160453	27.2678479
hsa-miR-101-5p	38.323453	36.5824241	36.1849628	35.423832	35.8086015
hsa-miR-103a-3p	29.001873	28.7274909	24.7477789	28.00745	28.3256549
hsa-miR-105-3p	ND	ND	ND	ND	ND
hsa-miR-106a-3p	ND	ND	37.7966993	ND	ND
hsa-miR-106a-5p	26.6471	25.682826	24.2604376	25.877963	26.0020153
hsa-miR-106b-3p	ND	35.8931336	30.8866013	35.946384	36.5919415
hsa-miR-106b-5p	28.335149	27.6725017	27.1532394	27.239982	27.4895592
hsa-miR-107	29.641393	29.1897193	26.2869828	28.636435	28.8961498
hsa-miR-10a-5p	ND	ND	36.4131125	38.13321	ND
hsa-miR-10b-5p	33.098603	32.6315072	32.7554513	33.285245	31.9962888
hsa-miR-1181	ND	ND	ND	ND	ND
hsa-miR-1183	ND	ND	ND	ND	ND
hsa-miR-1185-5p	ND	ND	36.295241	ND	ND
hsa-miR-1205	ND	ND	ND	ND	ND
hsa-miR-1207-5p	ND	ND	ND	ND	ND
hsa-miR-122-3p	36.259185	36.5574642	ND	ND	ND
hsa-miR-1224-3p	ND	ND	39.2979734	ND	ND
hsa-miR-122-5p	28.671523	29.0079976	30.7850554	30.228576	30.4438326

hsa-miR-1227-3p	ND	36.7115072	37.0728306	ND	39.3906305
hsa-miR-1237-3p	ND	ND	ND	ND	ND
hsa-miR-1238-3p	ND	ND	ND	ND	ND
hsa-miR-1243	ND	ND	ND	ND	ND
hsa-miR-124-3p	36.597374	ND	ND	36.184674	ND
hsa-miR-1245a	39.183269	ND	ND	39.232131	39.5371363
hsa-miR-1247-5p	ND	ND	ND	ND	ND
hsa-miR-1248	35.968213	ND	ND	ND	ND
hsa-miR-1249	36.597976	ND	33.9697348	36.529257	36.6084434
hsa-miR-1254	ND	ND	ND	ND	ND
hsa-miR-1255b-5p	37.120318	ND	36.7352649	36.816906	ND
hsa-miR-1256	ND	ND	36.7892068	ND	ND
hsa-miR-125a-3p	ND	ND	37.1805234	ND	ND
hsa-miR-125a-5p	32.985008	32.8679664	28.6734327	33.223322	33.8788222
hsa-miR-125b-2-3p	36.215751	ND	ND	38.880573	ND
hsa-miR-125b-5p	31.830918	32.31197	30.643566	31.685358	32.1831917
hsa-miR-1260a	33.90792	34.2737144	31.6273814	34.140089	34.8766313
hsa-miR-126-3p	28.161405	27.8877258	23.880532	27.881751	28.1242857
hsa-miR-1270	37.462475	ND	37.1902678	ND	ND
hsa-miR-1271-5p	ND	ND	34.3206351	ND	ND
hsa-miR-127-3p	ND	ND	30.3164425	ND	ND
hsa-miR-127-5p	ND	ND	36.5009256	ND	ND
hsa-miR-128	31.632651	30.9385556	28.8980028	30.840159	31.0065359
hsa-miR-129-5p	ND	ND	ND	ND	ND
hsa-miR-1296	ND	ND	36.9695339	ND	39.3683213
hsa-miR-130a-3p	31.640273	30.6395175	28.2819351	30.890135	30.9458663
hsa-miR-130b-3p	33.819519	32.7027695	30.6301154	33.023947	33.1587254
hsa-miR-130b-5p	ND	37.1183953	32.7964176	ND	ND
hsa-miR-132-3p	32.826996	33.1458561	31.5057677	33.578737	33.1252838
hsa-miR-132-5p	ND	ND	ND	ND	ND
hsa-miR-133a	34.262085	ND	31.9267024	36.700761	35.807903
hsa-miR-133b	35.644138	37.1389752	32.7165906	35.144401	ND
hsa-miR-134	39.296418	37.262834	31.9936455	38.479268	ND
hsa-miR-135a-3p	ND	ND	ND	ND	ND
hsa-miR-135a-5p	ND	39.2007386	35.6072784	36.176623	ND
hsa-miR-135b-5p	37.245162	36.512844	ND	35.664512	36.6026707
hsa-miR-136-3p	ND	ND	31.5824923	ND	35.9678409
hsa-miR-136-5p	ND	ND	30.9578049	ND	36.2780004
hsa-miR-139-3p	ND	ND	34.5656261	ND	ND
hsa-miR-139-5p	34.642086	ND	29.1253657	35.463826	35.9558568
hsa-miR-140-3p	27.887496	26.6020354	27.2934595	26.93861	27.1312221
hsa-miR-140-5p	32.425823	31.7196838	28.8515	31.344933	32.7239079
hsa-miR-141-3p	33.697915	34.3630563	32.6796758	32.825504	34.2802362
hsa-miR-141-5p	ND	ND	ND	ND	ND

hsa-miR-142-3p	29.55144	30.6358492	24.2801777	29.993683	30.6705338
hsa-miR-142-5p	32.007723	32.3016458	27.1912529	32.035086	32.2480307
hsa-miR-143-3p	32.719506	33.0745018	ND	33.280207	33.125658
hsa-miR-143-5p	ND	ND	37.2804415	39.203505	ND
hsa-miR-144-3p	26.71437	25.5968899	26.6520031	25.295792	25.3184888
hsa-miR-144-5p	32.666319	31.6544157	30.8112914	31.847253	33.4432505
hsa-miR-145-3p	36.009553	36.157373	33.7839732	36.855028	36.1869709
hsa-miR-145-5p	32.058201	32.4501108	28.806628	32.501236	32.5653861
hsa-miR-1468	ND	ND	36.4215993	ND	ND
hsa-miR-146a-5p	31.056592	31.4839203	26.028822	31.986821	31.8328523
hsa-miR-146b-3p	ND	ND	35.7494706	ND	ND
hsa-miR-146b-5p	34.317763	36.0579534	31.5994443	34.43708	34.0689801
hsa-miR-1471	ND	ND	ND	38.398608	ND
hsa-miR-147b	ND	36.6434071	ND	ND	ND
hsa-miR-148a-3p	30.458435	29.8666415	28.0100187	29.763658	29.7167318
hsa-miR-148b-3p	30.313179	28.9705549	26.8528843	29.269867	29.439154
hsa-miR-148b-5p	ND	ND	35.9639551	ND	ND
hsa-miR-149-5p	ND	ND	37.8557361	ND	ND
hsa-miR-150-5p	29.004607	30.4352511	30.2090834	30.802643	32.5453455
hsa-miR-151a-3p	33.339422	31.7012789	27.6182794	32.902969	31.6232861
hsa-miR-151a-5p	31.78618	30.8681001	26.3430102	31.255313	30.9939844
hsa-miR-152	32.229936	31.856041	28.3211363	31.538296	31.2599741
hsa-miR-153	ND	ND	36.2826963	39.349431	ND
hsa-miR-1537	36.901839	36.1718146	34.2199513	37.42941	36.1439054
hsa-miR-1538	ND	ND	37.7052856	ND	ND
hsa-miR-154-5p	ND	35.0669003	30.932035	ND	34.6582322
hsa-miR-155-5p	35.512046	ND	32.5379902	ND	36.8774789
hsa-miR-15a-3p	ND	37.0667916	37.9755848	ND	37.7803899
hsa-miR-15a-5p	25.566658	24.5597054	24.594216	24.624412	24.6419057
hsa-miR-15b-3p	31.150536	29.5111129	29.2445107	30.300991	30.2137645
hsa-miR-15b-5p	29.691727	28.7617568	26.5405756	28.562975	28.6950237
hsa-miR-16-1-3p	33.009006	32.2375938	32.9668881	32.729728	32.1804759
hsa-miR-16-2-3p	31.037927	29.9794114	30.1006466	30.236067	30.0447702
hsa-miR-16-5p	20.773806	19.5958412	19.9255728	20.161411	20.3321895
hsa-miR-17-3p	37.157374	35.0011066	34.0176032	35.202479	34.7798175
hsa-miR-17-5p	32.34699	31.6119134	30.782009	32.126411	31.9231802
hsa-miR-181a-2-3p	ND	ND	34.0390161	ND	ND
hsa-miR-181a-3p	ND	ND	35.3497702	37.495747	ND
hsa-miR-181a-5p	32.176097	33.3041758	29.0809695	32.469343	34.4483715
hsa-miR-181b-5p	35.739166	36.9374489	35.0734533	36.47372	38.9505724
hsa-miR-181c-3p	ND	ND	34.2556885	37.196462	ND
hsa-miR-181c-5p	39.065518	ND	35.0531774	ND	36.0314975
hsa-miR-181d	ND	ND	35.6634752	ND	ND
hsa-miR-182-3p	ND	ND	36.2791606	ND	ND

hsa-miR-182-5p	37.261845	36.6053446	ND	ND	ND
hsa-miR-183-3p	35.569738	37.0934577	36.927212	37.835515	ND
hsa-miR-183-5p	34.76224	34.4140007	35.6538943	33.781795	33.7989506
hsa-miR-184	ND	ND	ND	ND	ND
hsa-miR-185-3p	ND	ND	ND	38.617296	ND
hsa-miR-185-5p	28.154038	ND	26.4879213	26.833882	26.7359784
hsa-miR-186-5p	28.93864	27.8219375	27.4039777	28.340321	28.5370676
hsa-miR-187-3p	ND	ND	ND	ND	ND
hsa-miR-187-5p	ND	ND	ND	ND	ND
hsa-miR-188-3p	38.880756	ND	35.1252345	35.210766	37.1068039
hsa-miR-188-5p	35.439571	34.9620225	34.2152312	36.472432	36.0358236
hsa-miR-18a-3p	34.50256	32.8783784	32.8104345	32.348252	32.6903216
hsa-miR-18a-5p	30.298438	29.3479707	27.1805169	29.765721	29.926071
hsa-miR-18b-3p	ND	ND	ND	ND	ND
hsa-miR-18b-5p	29.824487	29.1717235	26.918304	28.919483	29.5695979
hsa-miR-1908	ND	36.0855739	31.7650598	38.036689	ND
hsa-miR-1909-3p	ND	ND	ND	ND	ND
hsa-miR-190a	ND	ND	37.0639978	39.090158	ND
hsa-miR-190b	39.041719	38.4032512	38.9560765	38.992335	ND
hsa-miR-1912	ND	ND	ND	ND	ND
hsa-miR-1913	ND	38.4138689	ND	ND	ND
hsa-miR-191-3p	ND	ND	33.9356563	ND	38.1374153
hsa-miR-1914-5p	ND	ND	ND	ND	ND
hsa-miR-191-5p	30.54201	30.1010791	26.0953654	30.29445	30.1466616
hsa-miR-192-3p	ND	ND	ND	ND	ND
hsa-miR-192-5p	29.905601	29.1614746	29.6229002	29.53744	29.5289849
hsa-miR-193a-3p	ND	ND	ND	ND	ND
hsa-miR-193a-5p	35.500611	34.7037381	34.7332208	35.515946	34.1363448
hsa-miR-193b-3p	35.942089	35.5791732	37.0399001	36.6534	34.9357743
hsa-miR-193b-5p	ND	ND	ND	ND	ND
hsa-miR-194-3p	38.547221	38.5483204	ND	ND	ND
hsa-miR-194-5p	31.391405	30.3078667	31.1798978	30.838205	31.037651
hsa-miR-195-5p	34.544774	34.2269758	31.0805987	34.540119	37.8045887
hsa-miR-196a-5p	38.477493	ND	38.1522286	ND	ND
hsa-miR-196b-3p	35.780432	ND	ND	ND	ND
hsa-miR-196b-5p	35.767995	34.6330612	33.2893048	34.933118	36.2163385
hsa-miR-1972	36.521373	36.1881597	37.8407207	35.198862	37.1719864
hsa-miR-197-3p	32.216797	32.9351261	28.1050518	32.318304	32.7360665
hsa-miR-199a-3p	32.111916	32.0657571	25.8952687	32.08336	32.2657717
hsa-miR-199a-5p	34.075126	36.7626804	27.706381	35.850382	35.9820716
hsa-miR-199b-5p	36.684594	ND	33.6568784	35.580027	36.4378527
hsa-miR-19a-3p	25.433168	24.2822526	23.7593699	24.486796	24.5307708
hsa-miR-19a-5p	ND	ND	35.8782918	39.561525	ND
hsa-miR-19b-1-5p	ND	ND	35.6707599	ND	ND

hsa-miR-19b-3p	24.541435	23.3628653	23.1206387	23.650148	23.8936082
hsa-miR-200a-3p	34.058713	34.2378898	34.2180999	34.8414	34.3463919
hsa-miR-200b-3p	35.194717	35.7448255	35.0215051	35.089967	35.129855
hsa-miR-200b-5p	ND	36.6915644	ND	36.288148	ND
hsa-miR-200c-3p	34.63441	33.5702438	31.504796	32.705163	33.61357
hsa-miR-200c-5p	37.031875	ND	ND	ND	ND
hsa-miR-202-3p	ND	ND	ND	ND	ND
hsa-miR-202-5p	ND	ND	ND	ND	39.2257341
hsa-miR-203a	ND	38.0503218	35.909394	35.597606	ND
hsa-miR-204-5p	37.153404	36.1331745	33.5980103	ND	ND
hsa-miR-205-5p	33.518393	33.6033151	33.1079875	32.727319	33.225596
hsa-miR-206	35.067446	35.5605334	35.9274471	34.942754	36.5268805
hsa-miR-208b	ND	ND	ND	ND	ND
hsa-miR-20a-3p	35.37569	36.5270594	33.2040128	36.535215	35.7084923
hsa-miR-20a-5p	25.56417	24.532178	23.7287592	24.862857	25.0389147
hsa-miR-20b-3p	ND	ND	37.0018241	ND	ND
hsa-miR-20b-5p	34.164146	33.0784344	34.5950475	33.199326	33.5668632
hsa-miR-210	31.88599	31.0763168	30.6210717	31.231478	30.8973095
hsa-miR-2110	34.632839	33.2639238	32.690658	33.875501	35.6247276
hsa-miR-211-5p	ND	39.1636505	ND	ND	ND
hsa-miR-212-3p	ND	ND	ND	ND	ND
hsa-miR-212-5p	ND	ND	ND	ND	ND
hsa-miR-21-3p	ND	37.1859764	33.6303984	ND	37.4808011
hsa-miR-214-3p	35.550118	ND	36.4454599	36.523077	37.3037093
hsa-miR-214-5p	ND	ND	37.6198205	ND	ND
hsa-miR-215	30.780803	30.6014793	30.5789925	30.170062	30.5838065
hsa-miR-21-5p	25.066556	24.3128804	22.2666131	24.688033	24.776091
hsa-miR-216a-5p	ND	38.3900132	ND	ND	36.9791061
hsa-miR-217	ND	36.5049194	ND	ND	ND
hsa-miR-218-2-3p	ND	ND	ND	ND	ND
hsa-miR-218-5p	ND	37.6071202	ND	ND	ND
hsa-miR-219-1-3p	ND	ND	ND	ND	ND
hsa-miR-219-5p	ND	ND	36.181591	ND	ND
hsa-miR-221-3p	30.93142	30.6767582	24.0175155	31.792905	30.6848277
hsa-miR-221-5p	ND	ND	35.3674526	36.298087	ND
hsa-miR-222-3p	28.782058	27.8429007	26.5422728	28.24438	28.3285093
hsa-miR-223-3p	25.007515	25.8678019	20.9258494	26.275099	25.8837691
hsa-miR-223-5p	37.09442	34.639296	32.2066443	36.538819	35.0401264
hsa-miR-22-3p	28.959007	27.8787976	26.2351707	28.213041	28.2812981
hsa-miR-224-3p	ND	ND	34.9238631	ND	ND
hsa-miR-224-5p	ND	ND	36.2290088	ND	ND
hsa-miR-22-5p	32.689901	32.7803918	29.3880673	32.081302	32.8586997
hsa-miR-23a-3p	27.51234	27.9182504	22.8620968	27.889001	27.6661624
hsa-miR-23a-5p	39.324345	ND	38.5489918	ND	ND

hsa-miR-23b-3p	30.447647	30.7136297	25.4519246	30.706241	30.8323791
hsa-miR-23b-5p	ND	ND	35.8753952	ND	36.1196728
hsa-miR-24-1-5p	36.6303	ND	36.6835589	ND	ND
hsa-miR-24-2-5p	ND	ND	31.9026285	38.939548	ND
hsa-miR-24-3p	28.492941	27.7239426	23.1973533	28.622176	28.3093442
hsa-miR-25-3p	26.685168	25.1974488	25.469213	25.826746	25.8260233
hsa-miR-25-5p	ND	35.9336976	35.0768397	ND	36.1937729
hsa-miR-26a-1-3p	ND	ND	34.7323047	ND	ND
hsa-miR-26a-2-3p	ND	ND	38.8529804	ND	37.6742916
hsa-miR-26a-5p	30.365097	31.2019462	25.6363945	30.230104	30.6440312
hsa-miR-26b-3p	37.381989	36.6234866	32.5699345	36.662224	35.6488219
hsa-miR-26b-5p	30.511855	30.3511105	28.5688086	30.140868	30.4986653
hsa-miR-27a-3p	31.56191	ND	ND	31.789698	31.3640623
hsa-miR-27a-5p	35.720292	36.6382793	ND	35.963225	36.8604787
hsa-miR-27b-3p	30.276481	30.6103985	25.1647714	30.338206	30.4717892
hsa-miR-27b-5p	ND	ND	ND	ND	ND
hsa-miR-28-3p	34.259325	33.9101871	29.6569717	34.801709	35.7396231
hsa-miR-28-5p	35.521382	ND	29.5271506	36.709195	39.2481769
hsa-miR-296-3p	ND	ND	ND	ND	ND
hsa-miR-296-5p	35.245681	34.2435389	33.3455269	35.775986	33.8960943
hsa-miR-299-3p	ND	ND	37.7915892	ND	ND
hsa-miR-299-5p	ND	ND	34.1647918	ND	ND
hsa-miR-29a-3p	30.67563	30.7912475	28.9792476	31.023709	31.5991888
hsa-miR-29a-5p	37.433769	ND	32.4564652	37.377604	35.7877051
hsa-miR-29b-2-5p	36.010494	34.5447624	33.937074	35.197543	34.9257262
hsa-miR-29b-3p	31.288068	30.5942266	29.2579846	30.453469	30.6624274
hsa-miR-29c-3p	29.507273	28.6189053	27.9134714	28.759042	28.8531862
hsa-miR-29c-5p	38.279119	36.847559	34.0484535	36.609777	36.9563168
hsa-miR-300	38.185052	ND	ND	ND	ND
hsa-miR-301a-3p	32.213168	32.4659568	28.8261691	31.734884	32.8658515
hsa-miR-301b	ND	36.4733012	32.17987	ND	36.7633328
hsa-miR-302c-5p	ND	ND	ND	ND	ND
hsa-miR-302d-5p	ND	ND	ND	ND	39.9447641
hsa-miR-30a-3p	38.701326	ND	34.4987923	38.046422	ND
hsa-miR-30a-5p	33.273625	33.0837003	31.9196839	33.508099	32.5632586
hsa-miR-30b-5p	31.322238	31.7084196	27.2367461	30.915936	32.204306
hsa-miR-30c-2-3p	ND	ND	39.4717906	ND	ND
hsa-miR-30c-5p	31.978505	32.4623586	27.9441872	31.506138	31.7274396
hsa-miR-30d-3p	ND	ND	34.5708577	ND	38.2948691
hsa-miR-30d-5p	33.035718	32.6122391	30.7888566	32.715999	32.3552295
hsa-miR-30e-3p	36.028059	ND	31.4193889	39.505932	35.3942167
hsa-miR-30e-5p	28.59525	27.5193877	25.9431498	27.69431	27.8931094
hsa-miR-31-3p	38.300373	ND	ND	ND	ND
hsa-miR-31-5p	36.074549	ND	ND	ND	ND

hsa-miR-320a	28.580783	26.9908345	26.3462851	27.663754	27.2260472
hsa-miR-320b	30.015247	28.7929234	28.0922982	29.700251	29.0276406
hsa-miR-320c	31.159199	30.0503665	29.5340546	29.998619	30.1960704
hsa-miR-320d	30.279297	29.2475516	28.6405324	29.840478	29.5604372
hsa-miR-323a-3p	ND	36.5908965	32.978663	ND	37.2163402
hsa-miR-32-3p	ND	ND	35.003315	ND	ND
hsa-miR-324-3p	31.016452	29.9220454	29.3032682	30.001639	30.5997033
hsa-miR-324-5p	33.960778	32.6622242	29.4872527	32.631764	33.543322
hsa-miR-32-5p	31.443213	30.5426931	30.2475533	30.883202	30.6884992
hsa-miR-326	ND	ND	31.2249531	36.794836	ND
hsa-miR-328	33.977689	34.1940451	29.1190128	35.094022	34.2689135
hsa-miR-329	36.14075	36.0325713	33.2369374	38.956755	36.6419722
hsa-miR-330-3p	ND	ND	32.6450472	ND	ND
hsa-miR-330-5p	ND	ND	ND	36.594576	39.0683197
hsa-miR-331-3p	35.187378	35.7717077	31.873592	35.686359	35.3227128
hsa-miR-331-5p	ND	ND	36.0476	ND	ND
hsa-miR-335-3p	39.029629	35.4567685	30.3367227	ND	35.4843729
hsa-miR-335-5p	32.819715	33.7229762	29.3856217	33.597824	33.7727707
hsa-miR-337-3p	36.145642	ND	30.9506907	ND	36.9008057
hsa-miR-337-5p	ND	ND	33.6672138	ND	ND
hsa-miR-338-3p	33.989678	35.1756317	30.2546676	34.969484	34.660114
hsa-miR-338-5p	ND	ND	37.6771229	ND	ND
hsa-miR-339-3p	34.714649	33.1078543	29.8265515	33.817291	34.7522838
hsa-miR-339-5p	37.620888	ND	31.2590858	ND	38.9295081
hsa-miR-33a-3p	37.360762	ND	35.0670282	ND	ND
hsa-miR-33a-5p	35.48231	36.6844205	30.7246691	35.69278	35.7630824
hsa-miR-33b-3p	ND	ND	ND	ND	ND
hsa-miR-33b-5p	36.490032	34.3787451	35.103024	35.094643	37.0788053
hsa-miR-340-3p	38.857542	ND	33.9971276	ND	ND
hsa-miR-340-5p	ND	ND	35.9728592	39.030445	ND
hsa-miR-342-3p	30.749173	31.6675997	29.7280001	31.896695	31.9779351
hsa-miR-342-5p	35.934767	36.887939	36.0338632	37.647357	ND
hsa-miR-345-5p	ND	ND	35.8546383	38.077408	ND
hsa-miR-346	36.931357	ND	ND	37.32331	39.1221651
hsa-miR-34a-3p	ND	37.7115069	36.8479452	36.799709	38.7315812
hsa-miR-34a-5p	33.965054	32.0879832	28.8611004	32.073074	32.1164549
hsa-miR-34b-3p	39.310788	ND	ND	ND	ND
hsa-miR-34c-5p	ND	ND	ND	ND	ND
hsa-miR-361-3p	35.15643	36.7993201	32.6305893	36.18374	35.9274606
hsa-miR-361-5p	31.129657	31.5265209	27.5609919	31.620434	31.9103399
hsa-miR-362-3p	33.537322	32.4775592	31.3093764	32.172361	32.0376314
hsa-miR-362-5p	38.046121	39.4257815	35.8471571	37.615633	37.2627166
hsa-miR-363-3p	29.449351	28.2774243	28.7658724	28.675544	28.6428103
hsa-miR-363-5p	ND	35.9599085	ND	ND	ND

hsa-miR-365a-3p	34.563499	34.9204175	34.1330924	35.000575	34.7967028
hsa-miR-369-3p	ND	ND	36.472691	39.023539	38.259404
hsa-miR-369-5p	ND	ND	35.8647969	ND	ND
hsa-miR-370	ND	ND	33.9294764	ND	ND
hsa-miR-373-3p	ND	ND	ND	ND	ND
hsa-miR-373-5p	ND	ND	ND	ND	ND
hsa-miR-374a-5p	34.523528	34.4476636	30.0521256	33.088922	33.8008182
hsa-miR-374b-3p	ND	ND	36.4884092	ND	ND
hsa-miR-374b-5p	35.28269	35.9741647	29.4951544	35.024618	35.8841891
hsa-miR-375	31.447692	31.3148856	32.1424213	32.887204	31.8373601
hsa-miR-376a-3p	36.246124	35.7914988	ND	ND	37.732023
hsa-miR-376a-5p	ND	ND	39.4355929	ND	ND
hsa-miR-376b-3p	ND	36.8843407	32.7006918	ND	ND
hsa-miR-376c-3p	35.627118	34.7509997	29.0568683	37.347615	37.628621
hsa-miR-377-3p	38.220144	37.8563813	33.6016675	ND	38.0091403
hsa-miR-377-5p	ND	ND	35.2248921	ND	ND
hsa-miR-378a-3p	31.105992	29.6644144	29.5495141	30.149938	30.282354
hsa-miR-378a-5p	35.452226	36.9231602	34.9793117	34.867565	36.5867224
hsa-miR-379-3p	ND	ND	35.7196322	ND	ND
hsa-miR-379-5p	ND	ND	32.9886322	ND	ND
hsa-miR-380-3p	ND	ND	36.1100005	38.316143	ND
hsa-miR-381-3p	ND	ND	35.320463	ND	ND
hsa-miR-382-3p	ND	ND	34.2869765	ND	ND
hsa-miR-382-5p	ND	34.6931253	30.0790951	ND	36.9754614
hsa-miR-409-3p	ND	ND	29.3535138	ND	35.7680282
hsa-miR-409-5p	ND	ND	33.5283079	ND	ND
hsa-miR-410	38.295527	ND	31.6381341	ND	36.7367846
hsa-miR-411-5p	ND	ND	33.4774893	ND	ND
hsa-miR-412	ND	ND	ND	ND	ND
hsa-miR-421	35.43349	33.7910955	31.1777819	34.748947	33.7994038
hsa-miR-423-3p	32.094365	32.0297365	26.9613493	32.665508	32.2711847
hsa-miR-423-5p	29.829221	28.5974674	27.7421255	29.06247	29.0221701
hsa-miR-424-3p	36.004061	35.0384627	34.1575802	35.040493	35.9517163
hsa-miR-424-5p	31.26715	30.7239783	29.9267293	29.802257	30.4953662
hsa-miR-425-3p	ND	31.8303262	29.868497	32.245967	32.5692484
hsa-miR-425-5p	28.860601	27.7651963	26.6634855	28.110828	28.173132
hsa-miR-429	37.039901	36.2941806	36.7027949	36.207311	37.1660851
hsa-miR-431-3p	ND	ND	ND	ND	ND
hsa-miR-431-5p	ND	ND	31.8022466	ND	ND
hsa-miR-432-3p	ND	ND	ND	ND	ND
hsa-miR-432-5p	37.672459	ND	32.7139522	ND	37.232127
hsa-miR-433	ND	37.5939332	34.2735949	ND	ND
hsa-miR-449a	ND	ND	ND	38.243671	ND
hsa-miR-449b-5p	ND	ND	ND	ND	ND

hsa-miR-450a-5p	ND	ND	39.0307426	ND	ND
hsa-miR-450b-3p	ND	ND	38.2323238	ND	ND
hsa-miR-450b-5p	ND	36.8143487	36.2341609	35.906122	39.2009327
hsa-miR-451a	20.264935	19.1653856	19.9374421	19.184984	19.5417082
hsa-miR-452-5p	ND	ND	34.7492409	ND	ND
hsa-miR-454-3p	32.086808	32.459099	30.6599215	32.116798	33.1181722
hsa-miR-454-5p	ND	ND	36.126942	ND	37.9726659
hsa-miR-455-3p	35.559568	ND	ND	ND	35.9851263
hsa-miR-455-5p	ND	ND	ND	ND	ND
hsa-miR-483-3p	ND	ND	ND	39.307038	ND
hsa-miR-483-5p	36.135571	36.4824093	36.9036076	37.895208	36.6249796
hsa-miR-484	29.49554	28.2963143	26.6075259	28.66583	28.6563474
hsa-miR-486-3p	34.874728	32.8444797	33.7169442	33.188738	33.2514686
hsa-miR-486-5p	25.809183	24.239627	25.3011365	25.001398	24.7683141
hsa-miR-487a	ND	38.8914449	35.1924045	ND	ND
hsa-miR-487b	ND	ND	32.8787666	ND	ND
hsa-miR-489	ND	ND	ND	36.875404	ND
hsa-miR-490-3p	32.954084	32.6481848	33.5852416	32.887537	32.7442
hsa-miR-490-5p	ND	ND	36.5466816	ND	ND
hsa-miR-491-5p	ND	35.9814135	32.0336367	36.139524	ND
hsa-miR-493-3p	36.552042	ND	33.362625	ND	ND
hsa-miR-493-5p	ND	ND	33.3190285	ND	ND
hsa-miR-494	ND	ND	36.5705892	ND	ND
hsa-miR-495-3p	34.80718	35.2485205	30.4821985	35.873897	35.5914299
hsa-miR-496	ND	ND	34.1561386	ND	38.7270732
hsa-miR-497-5p	32.689281	34.8934033	31.2425123	33.341345	33.7659127
hsa-miR-499a-5p	ND	ND	ND	ND	ND
hsa-miR-500a-5p	35.770415	35.752949	36.7464158	35.21419	37.3460894
hsa-miR-501-3p	33.947136	33.8628956	32.8857466	33.661102	34.246824
hsa-miR-501-5p	34.205982	33.9660674	36.1015329	34.195131	34.1807975
hsa-miR-502-3p	34.269623	31.7133371	32.3135693	31.676536	32.3335885
hsa-miR-502-5p	36.256621	36.7610262	37.4724223	36.166918	36.9082883
hsa-miR-503-5p	ND	ND	32.1197293	36.39479	ND
hsa-miR-505-3p	33.752052	33.9015629	31.0525045	33.922014	33.8493599
hsa-miR-505-5p	35.986386	34.8868114	35.0986561	35.944882	35.4632359
hsa-miR-507	ND	ND	ND	ND	ND
hsa-miR-508-3p	ND	ND	ND	ND	ND
hsa-miR-509-3-5p	ND	ND	39.543347	ND	ND
hsa-miR-509-3p	ND	ND	ND	ND	ND
hsa-miR-511	ND	ND	ND	ND	36.5417673
hsa-miR-513a-5p	ND	ND	ND	ND	39.0457456
hsa-miR-514a-3p	37.783436	ND	ND	ND	ND
hsa-miR-515-3p	ND	ND	ND	ND	ND
hsa-miR-517c-3p	ND	ND	ND	ND	ND

hsa-miR-518d-3p	ND	ND	ND	ND	ND
hsa-miR-518f-3p	ND	ND	ND	ND	ND
hsa-miR-518f-5p	ND	ND	ND	ND	ND
hsa-miR-519b-3p	ND	ND	ND	ND	ND
hsa-miR-520a-5p	ND	ND	ND	ND	ND
hsa-miR-520c-3p	ND	ND	ND	ND	ND
hsa-miR-520g	ND	ND	ND	ND	ND
hsa-miR-520h	ND	36.9665889	38.130532	37.195365	ND
hsa-miR-524-3p	ND	ND	36.9208082	ND	ND
hsa-miR-525-3p	ND	ND	ND	ND	ND
hsa-miR-525-5p	ND	ND	ND	ND	37.6404709
hsa-miR-532-3p	32.895768	32.5015557	31.8141461	32.737048	33.3029177
hsa-miR-532-5p	31.516965	30.3575316	30.2987444	30.706203	30.738457
hsa-miR-539-5p	ND	ND	33.544958	ND	ND
hsa-miR-542-5p	ND	ND	34.292902	ND	34.9137746
hsa-miR-543	39.022413	38.4807066	31.6382768	ND	ND
hsa-miR-544a	ND	ND	37.429631	ND	ND
hsa-miR-545-3p	ND	37.7810778	ND	39.239192	36.428845
hsa-miR-548a-3p	38.028922	ND	30.5034428	36.124816	38.4576966
hsa-miR-548a-5p	ND	ND	36.7253138	ND	ND
hsa-miR-548b-3p	ND	ND	37.4758451	ND	ND
hsa-miR-548c-5p	37.486547	37.8789531	33.8701941	37.893869	36.6129013
hsa-miR-548d-3p	ND	ND	ND	ND	ND
hsa-miR-548d-5p	ND	ND	37.3626053	36.441264	ND
hsa-miR-548e	ND	ND	39.119468	ND	ND
hsa-miR-548j	ND	35.6237827	33.8552366	36.395815	ND
hsa-miR-548k	ND	ND	35.8378314	ND	35.9412517
hsa-miR-548l	ND	ND	ND	ND	ND
hsa-miR-548n	ND	ND	ND	ND	ND
hsa-miR-549a	ND	36.5592715	ND	ND	ND
hsa-miR-550a-3p	34.101327	32.817493	33.9151635	32.951628	32.9766202
hsa-miR-550a-5p	ND	35.3357622	ND	ND	ND
hsa-miR-551a	35.681989	37.0000697	ND	ND	36.407476
hsa-miR-551b-3p	35.467577	ND	32.486405	ND	ND
hsa-miR-551b-5p	ND	ND	ND	ND	ND
hsa-miR-556-3p	ND	ND	32.6167941	ND	ND
hsa-miR-564	ND	ND	ND	ND	ND
hsa-miR-570-3p	37.441045	39.178655	34.2938808	37.715995	36.2302639
hsa-miR-571	ND	ND	ND	ND	ND
hsa-miR-573	ND	ND	ND	ND	ND
hsa-miR-574-3p	33.43378	33.096802	29.0434276	33.3206	33.4718699
hsa-miR-576-3p	38.174366	ND	35.1002126	37.107164	ND
hsa-miR-576-5p	ND	ND	36.5846226	ND	ND
hsa-miR-579	ND	ND	36.1763047	ND	37.0017546

hsa-miR-580	ND	ND	ND	ND	ND
hsa-miR-581	ND	ND	ND	ND	ND
hsa-miR-582-5p	34.843252	ND	ND	35.861461	35.5626257
hsa-miR-584-5p	34.046512	34.0461969	30.5388511	34.723057	33.968131
hsa-miR-589-3p	ND	ND	38.2324423	ND	ND
hsa-miR-589-5p	ND	ND	37.6277662	ND	37.4507069
hsa-miR-590-3p	34.693705	38.1549989	31.9154597	35.500405	34.9945854
hsa-miR-590-5p	31.771185	30.4803962	28.8799707	31.291014	30.9328901
hsa-miR-596	ND	ND	ND	ND	ND
hsa-miR-597	ND	ND	38.0188036	ND	ND
hsa-miR-598	34.958009	34.2631793	31.7638678	34.622481	34.573668
hsa-miR-601	ND	ND	ND	ND	ND
hsa-miR-604	ND	ND	ND	ND	ND
hsa-miR-605	ND	ND	37.1836315	ND	ND
hsa-miR-610	ND	36.8430854	ND	37.461759	ND
hsa-miR-612	ND	ND	ND	ND	ND
hsa-miR-615-3p	ND	ND	ND	ND	ND
hsa-miR-616-5p	ND	36.9899384	35.6344449	35.338541	36.2384591
hsa-miR-618	ND	ND	ND	ND	ND
hsa-miR-621	ND	ND	ND	ND	ND
hsa-miR-624-5p	29.599872	32.1121372	32.3023115	31.84548	31.9245921
hsa-miR-625-3p	38.419207	ND	32.5869835	36.409948	36.1716985
hsa-miR-626	ND	ND	ND	ND	ND
hsa-miR-627	36.547485	35.6333856	35.1939678	35.53639	35.8856291
hsa-miR-628-3p	35.518023	37.6589616	33.7452473	37.370521	ND
hsa-miR-628-5p	ND	ND	34.1918485	ND	ND
hsa-miR-629-3p	ND	37.612688	34.7548398	36.30302	36.9885698
hsa-miR-629-5p	32.867271	31.7622554	31.7404538	32.01857	32.731022
hsa-miR-636	ND	ND	36.2928745	33.917131	35.9927346
hsa-miR-641	ND	ND	39.2443707	ND	ND
hsa-miR-642a-5p	ND	36.7807008	ND	ND	ND
hsa-miR-643	ND	ND	ND	ND	ND
hsa-miR-650	ND	38.4484834	ND	ND	ND
hsa-miR-651	ND	36.5720548	33.9769337	35.238658	ND
hsa-miR-652-3p	30.841515	29.8411636	26.8167564	29.816224	30.2742453
hsa-miR-654-3p	ND	ND	33.3665175	ND	ND
hsa-miR-654-5p	ND	ND	33.693987	ND	ND
hsa-miR-655	37.355355	37.9552589	34.2567243	36.765733	37.7889434
hsa-miR-659-3p	ND	ND	ND	ND	ND
hsa-miR-660-5p	30.316116	29.0013582	28.9038561	29.290267	29.2619594
hsa-miR-662	39.26894	ND	ND	ND	ND
hsa-miR-663a	35.905903	38.0119397	37.0772566	36.600575	36.1572087
hsa-miR-664a-3p	38.22044	38.1387353	32.9576398	ND	36.8837754
hsa-miR-665	ND	ND	ND	ND	ND

hsa-miR-668	ND	ND	ND	ND	ND
hsa-miR-671-3p	ND	ND	34.5265461	ND	36.1750543
hsa-miR-671-5p	37.291836	ND	35.637142	ND	39.1699847
hsa-miR-675-3p	ND	ND	37.312216	37.599908	ND
hsa-miR-675-5p	ND	ND	ND	ND	ND
hsa-miR-708-3p	37.141433	36.8750581	36.2900472	38.038151	36.0337662
hsa-miR-7-1-3p	33.693552	33.480651	31.6134736	33.904946	33.7165121
hsa-miR-744-3p	37.583555	ND	33.9020371	ND	ND
hsa-miR-744-5p	38.66805	36.1312236	31.5248861	37.904987	36.092904
hsa-miR-758-3p	ND	ND	34.5462914	ND	ND
hsa-miR-7-5p	32.730466	31.7625358	32.5945663	31.762483	31.7399598
hsa-miR-760	ND	ND	ND	ND	ND
hsa-miR-765	ND	ND	ND	ND	ND
hsa-miR-766-3p	ND	ND	29.7627106	36.471701	35.6737069
hsa-miR-769-3p	ND	ND	ND	ND	ND
hsa-miR-769-5p	ND	36.4677437	33.1247561	ND	ND
hsa-miR-770-5p	ND	ND	ND	ND	ND
hsa-miR-873-5p	ND	37.9996103	ND	ND	ND
hsa-miR-874	34.273014	33.6253403	33.0294149	34.708423	ND
hsa-miR-876-3p	ND	ND	ND	ND	ND
hsa-miR-877-3p	ND	ND	35.4597409	ND	ND
hsa-miR-877-5p	34.726813	33.3538942	31.8805205	34.516799	34.6956921
hsa-miR-885-5p	34.922494	35.776739	36.1507683	37.315053	35.9595637
hsa-miR-887	ND	35.8501474	36.9813483	ND	37.0642471
hsa-miR-888-5p	ND	ND	ND	ND	ND
hsa-miR-889	ND	ND	34.8091318	ND	ND
hsa-miR-92a-1-5p	ND	ND	36.474714	ND	ND
hsa-miR-92a-3p	24.996104	23.6991535	23.9678865	24.329872	24.1851125
hsa-miR-92b-3p	37.422858	35.1128435	34.1659282	36.956934	35.2281813
hsa-miR-92b-5p	ND	ND	ND	36.879019	ND
hsa-miR-93-3p	31.866747	32.0638822	30.5535455	32.450084	32.9518229
hsa-miR-934	ND	36.5216672	ND	ND	36.8511058
hsa-miR-93-5p	26.53781	25.4904793	24.9051719	25.660015	25.7294012
hsa-miR-9-3p	ND	ND	35.9176785	ND	ND
hsa-miR-940	ND	ND	34.0711086	ND	ND
hsa-miR-941	ND	35.1823972	32.3051237	34.759641	38.0491998
hsa-miR-942	35.701497	33.7587725	33.6025284	34.967227	35.1008177
hsa-miR-95	34.729979	ND	ND	35.684569	ND
hsa-miR-9-5p	ND	ND	36.7030408	ND	36.9101067
hsa-miR-96-5p	34.729702	34.9642339	33.911067	33.554174	34.4704499
hsa-miR-98-5p	35.67373	34.5567631	30.9997814	34.892803	34.8996594
hsa-miR-99a-3p	ND	ND	36.4159311	ND	ND
hsa-miR-99a-5p	32.927803	34.3352748	32.190443	33.566181	33.6468231
hsa-miR-99b-3p	39.3834	37.4563329	33.8332032	35.201845	ND

hsa-miR-99b-5p	34.172479	34.1211721	30.6591769	36.299506	33.7910991
SNORD38B	ND	ND	ND	ND	ND
SNORD49A	ND	ND	36.1198832	ND	35.6757008
UniSp2 CP	19.147173	19.5626712	19.6582268	19.360009	19.6457285
UniSp3 IPC	20.73406	20.5654029	20.9846834	21.033104	20.7952425
UniSp3 IPC	19.998265	19.8417403	20.1853867	20.024901	19.9611261
UniSp4 CP	25.589494	25.8174972	25.951844	25.718913	26.024815
UniSp5 CP	30.770896	31.1543569	31.0228201	31.478969	31.5120637
UniSp6 CP	19.786808	20.1351337	20.7891533	20.011543	19.7156567

Table S1, continued

**Patient ID**	**2534**				
**Dose**	**0 Gy**	**10 Gy**	**28 Gy**	**38 Gy**	**62 Gy**
**Date**	**4/2/2014**	**4/21/2014**	**5/2/2014**	**5/12/2014**	**6/2/2014**
cel-miR-39-3p CP	36.204545	ND	ND	36.8506314	ND
hsa-let-7a-2-3p	ND	ND	ND	ND	ND
hsa-let-7a-3p	36.458986	37.0333619	36.938526	ND	32.80235
hsa-let-7a-5p	29.735263	30.3254545	30.080347	30.7346841	27.719997
hsa-let-7b-3p	33.870117	33.8937439	39.469019	33.9523197	31.299361
hsa-let-7b-5p	28.047693	27.6064964	26.847493	28.8192273	23.588805
hsa-let-7c	33.64938	33.8792765	33.699467	35.6552754	30.972723
hsa-let-7d-3p	30.154226	30.0614994	29.765726	30.7486162	26.752679
hsa-let-7d-5p	33.998309	32.6392818	32.502827	33.5736496	29.537117
hsa-let-7e-3p	ND	ND	ND	37.2876855	ND
hsa-let-7e-5p	36.524243	35.0857636	35.885907	36.3936934	32.608511
hsa-let-7f-1-3p	36.84133	37.0416064	ND	ND	32.691992
hsa-let-7f-2-3p	38.423751	37.9924534	ND	ND	35.20253
hsa-let-7f-5p	33.940632	34.6662021	33.491491	34.0230774	31.609459
hsa-let-7g-3p	ND	37.0340599	34.629501	35.5128218	33.084053
hsa-let-7g-5p	29.164059	29.3084883	28.30888	29.9066037	25.203043
hsa-let-7i-3p	37.209032	ND	37.332257	37.3812768	34.300192
hsa-let-7i-5p	28.781379	28.7276144	27.980548	29.035237	24.760245
hsa-miR-1	37.052025	37.0022487	ND	ND	35.703166
hsa-miR-100-5p	34.562046	33.7247622	32.590329	33.6643651	33.140503
hsa-miR-101-3p	27.78859	27.9006469	27.010272	28.6673416	23.627926
hsa-miR-101-5p	36.899968	36.9385339	36.536276	37.1613283	32.025884
hsa-miR-103a-3p	29.220538	29.1528425	28.276834	29.9398483	25.499107
hsa-miR-105-3p	ND	ND	38.365446	ND	ND
hsa-miR-106a-3p	ND	ND	ND	ND	ND
hsa-miR-106a-5p	27.260764	27.2073798	26.287655	27.5230311	22.481459
hsa-miR-106b-3p	ND	35.2533347	35.634927	37.2662679	32.146328
hsa-miR-106b-5p	28.338254	28.0887945	27.714604	29.4575449	24.219432
hsa-miR-107	29.824901	29.5590946	29.229107	30.4731016	25.893204
hsa-miR-10a-5p	37.208071	37.5970695	ND	39.5780118	37.874459
hsa-miR-10b-5p	32.700483	32.8506148	32.933344	32.6910485	31.59466
hsa-miR-1181	ND	ND	ND	ND	ND
hsa-miR-1183	ND	ND	ND	ND	39.459274
hsa-miR-1185-5p	ND	ND	ND	ND	ND
hsa-miR-1205	ND	ND	ND	ND	ND
hsa-miR-1207-5p	ND	ND	ND	ND	ND
hsa-miR-122-3p	ND	ND	ND	ND	ND
hsa-miR-1224-3p	ND	ND	ND	ND	ND
hsa-miR-122-5p	29.237433	28.9635203	27.786538	29.4275831	27.828423

hsa-miR-1227-3p	36.92101	ND	ND	ND	35.97926
hsa-miR-1237-3p	ND	ND	ND	ND	ND
hsa-miR-1238-3p	ND	ND	ND	ND	ND
hsa-miR-1243	ND	ND	ND	39.0469566	ND
hsa-miR-124-3p	ND	ND	ND	ND	ND
hsa-miR-1245a	ND	39.4482966	ND	ND	37.467198
hsa-miR-1247-5p	ND	ND	ND	ND	ND
hsa-miR-1248	ND	ND	ND	ND	ND
hsa-miR-1249	ND	37.2102807	36.861824	38.0970862	36.85262
hsa-miR-1254	ND	ND	ND	ND	ND
hsa-miR-1255b-5p	ND	ND	ND	ND	34.133379
hsa-miR-1256	ND	ND	ND	ND	ND
hsa-miR-125a-3p	ND	ND	ND	ND	ND
hsa-miR-125a-5p	32.995571	33.9194533	34.237246	33.2461292	32.026303
hsa-miR-125b-2-3p	ND	36.2859072	37.061004	ND	ND
hsa-miR-125b-5p	31.630414	31.5306142	31.827825	32.3429109	30.831507
hsa-miR-1260a	32.69378	32.634532	35.463284	ND	32.294227
hsa-miR-126-3p	27.784615	27.9628532	27.971268	28.2868591	25.748046
hsa-miR-1270	ND	ND	ND	ND	34.284122
hsa-miR-1271-5p	ND	ND	36.777038	ND	38.206265
hsa-miR-127-3p	35.113542	ND	ND	ND	35.550112
hsa-miR-127-5p	ND	36.8732361	ND	ND	36.99041
hsa-miR-128	31.558321	31.3995017	30.702534	31.783378	27.606854
hsa-miR-129-5p	ND	ND	ND	ND	ND
hsa-miR-1296	ND	ND	ND	ND	ND
hsa-miR-130a-3p	32.022171	31.4724029	31.070968	33.5008542	27.989943
hsa-miR-130b-3p	34.116017	34.0172173	33.318269	35.4912815	30.653818
hsa-miR-130b-5p	ND	ND	ND	ND	35.959556
hsa-miR-132-3p	33.937919	34.0933094	32.542688	35.4854287	31.041911
hsa-miR-132-5p	ND	ND	ND	ND	ND
hsa-miR-133a	33.675445	34.9876102	ND	35.5472049	34.458966
hsa-miR-133b	35.923647	37.5966086	36.331082	ND	35.90247
hsa-miR-134	36.307539	36.6035955	37.220197	ND	36.995357
hsa-miR-135a-3p	ND	ND	ND	ND	ND
hsa-miR-135a-5p	37.995378	ND	ND	ND	34.59183
hsa-miR-135b-5p	37.451407	ND	ND	ND	ND
hsa-miR-136-3p	ND	39.5962865	35.811438	39.2359162	ND
hsa-miR-136-5p	36.451535	ND	ND	ND	35.862368
hsa-miR-139-3p	ND	ND	ND	ND	37.492172
hsa-miR-139-5p	36.547145	35.2443285	35.085554	35.0442716	33.031261
hsa-miR-140-3p	27.970774	27.9029775	27.304418	28.5609451	24.128304
hsa-miR-140-5p	33.519801	32.5513933	31.685357	33.275534	27.714482
hsa-miR-141-3p	35.424973	34.1584975	35.118387	36.924755	33.905775
hsa-miR-141-5p	ND	ND	36.865851	ND	ND

hsa-miR-142-3p	29.072225	30.3284569	29.822789	30.400981	28.299304
hsa-miR-142-5p	32.58823	32.4784791	31.939296	33.6180579	29.006411
hsa-miR-143-3p	33.204159	32.0541815	32.417696	33.8662787	31.400599
hsa-miR-143-5p	ND	ND	ND	ND	37.312985
hsa-miR-144-3p	26.287056	26.4343151	25.574325	27.9035405	22.132523
hsa-miR-144-5p	32.257962	32.9409305	31.701779	33.2948253	29.585413
hsa-miR-145-3p	35.082258	34.9732002	37.235086	34.9445733	34.728568
hsa-miR-145-5p	32.028699	31.5669305	31.207051	32.251656	30.819686
hsa-miR-1468	ND	ND	ND	ND	ND
hsa-miR-146a-5p	31.044579	30.5617288	30.921834	32.1690783	30.030009
hsa-miR-146b-3p	36.796634	ND	ND	ND	ND
hsa-miR-146b-5p	33.91572	34.1911853	33.923823	35.7639391	31.735813
hsa-miR-1471	ND	ND	ND	ND	38.350266
hsa-miR-147b	ND	ND	37.893392	ND	ND
hsa-miR-148a-3p	30.652568	30.2054047	29.656102	31.9950925	26.561382
hsa-miR-148b-3p	30.576073	30.1933609	29.634847	30.7426836	26.443385
hsa-miR-148b-5p	ND	ND	ND	ND	ND
hsa-miR-149-5p	ND	ND	ND	ND	ND
hsa-miR-150-5p	29.473633	30.2131768	29.901105	31.0251986	29.67504
hsa-miR-151a-3p	32.79021	32.1533665	32.273988	33.2527355	29.008588
hsa-miR-151a-5p	31.84701	31.7676114	31.63773	32.8228377	28.739339
hsa-miR-152	32.261687	32.1636227	31.89007	32.6078111	29.247185
hsa-miR-153	ND	ND	36.58396	ND	35.681979
hsa-miR-1537	35.684817	37.8027231	37.205583	38.201769	34.350796
hsa-miR-1538	ND	ND	ND	ND	ND
hsa-miR-154-5p	35.038813	35.9590442	ND	ND	ND
hsa-miR-155-5p	36.706942	ND	36.054123	38.9066776	35.481903
hsa-miR-15a-3p	36.148602	36.6125283	ND	ND	33.041971
hsa-miR-15a-5p	25.442038	25.1045351	24.820567	26.2241708	21.488793
hsa-miR-15b-3p	30.653156	31.0613087	30.452506	31.4977888	26.753612
hsa-miR-15b-5p	29.463601	29.0444059	28.917336	30.2138265	24.629438
hsa-miR-16-1-3p	33.210607	34.902252	33.516106	33.2768287	30.248783
hsa-miR-16-2-3p	30.801221	30.9819189	30.589265	31.6835287	27.317377
hsa-miR-16-5p	21.316453	21.2347229	20.469511	21.4978566	17.36427
hsa-miR-17-3p	35.916229	35.691137	35.428539	36.7780631	30.673142
hsa-miR-17-5p	33.439258	32.6502444	32.667135	33.764093	28.195132
hsa-miR-181a-2-3p	39.348493	ND	38.531006	ND	ND
hsa-miR-181a-3p	ND	ND	ND	ND	ND
hsa-miR-181a-5p	31.908731	32.2631562	32.456113	33.5340643	29.83501
hsa-miR-181b-5p	36.598053	37.1604726	38.201695	37.0200857	34.100377
hsa-miR-181c-3p	ND	ND	ND	ND	ND
hsa-miR-181c-5p	36.461742	ND	ND	ND	35.978552
hsa-miR-181d	ND	37.48952	ND	ND	ND
hsa-miR-182-3p	ND	ND	ND	ND	35.82198

hsa-miR-182-5p	36.941017	38.7918015	ND	36.9872678	35.338398
hsa-miR-183-3p	35.917845	36.5013155	ND	ND	33.086289
hsa-miR-183-5p	37.110555	35.5600006	34.966446	37.044617	30.239972
hsa-miR-184	ND	ND	ND	ND	ND
hsa-miR-185-3p	37.661421	ND	36.019966	ND	33.6783
hsa-miR-185-5p	28.01226	27.6019311	27.361575	28.4844454	23.022649
hsa-miR-186-5p	29.974733	29.4929732	28.860786	30.1065633	25.118901
hsa-miR-187-3p	ND	ND	ND	38.0064974	ND
hsa-miR-187-5p	ND	ND	ND	36.2539676	ND
hsa-miR-188-3p	35.550094	37.340582	35.226127	ND	35.518339
hsa-miR-188-5p	35.150699	ND	34.559919	35.8739966	31.914793
hsa-miR-18a-3p	34.498438	36.4491268	33.384987	34.3899537	28.945448
hsa-miR-18a-5p	31.605501	31.2694931	29.999104	30.9503004	25.697137
hsa-miR-18b-3p	ND	ND	ND	ND	36.072785
hsa-miR-18b-5p	30.674003	31.1571497	29.456307	30.2587194	25.248449
hsa-miR-1908	ND	ND	37.324329	ND	35.097624
hsa-miR-1909-3p	ND	ND	ND	ND	ND
hsa-miR-190a	38.024652	ND	38.986074	ND	36.97816
hsa-miR-190b	ND	ND	38.053895	ND	35.361838
hsa-miR-1912	ND	ND	ND	ND	35.769824
hsa-miR-1913	ND	37.65542	37.908614	ND	ND
hsa-miR-191-3p	ND	ND	ND	ND	ND
hsa-miR-1914-5p	ND	ND	ND	ND	ND
hsa-miR-191-5p	31.435775	31.2632884	30.591873	31.8422209	27.057558
hsa-miR-192-3p	ND	ND	ND	ND	ND
hsa-miR-192-5p	30.76064	30.582444	29.52761	30.8341594	26.508872
hsa-miR-193a-3p	ND	38.3814994	38.217708	ND	ND
hsa-miR-193a-5p	34.879399	35.103197	34.356113	36.3244908	33.043409
hsa-miR-193b-3p	35.997931	33.9422961	34.367965	35.8094145	33.285903
hsa-miR-193b-5p	ND	ND	38.987792	ND	37.740597
hsa-miR-194-3p	ND	ND	38.957228	ND	ND
hsa-miR-194-5p	31.537285	31.6384202	30.937978	32.7795922	28.411246
hsa-miR-195-5p	33.876378	33.7812132	33.282083	34.0373538	32.51828
hsa-miR-196a-5p	ND	37.9648211	ND	ND	ND
hsa-miR-196b-3p	ND	ND	ND	ND	ND
hsa-miR-196b-5p	35.575297	36.2015379	35.542297	35.7609536	32.120048
hsa-miR-1972	34.363163	33.9562267	34.619069	35.2011369	30.930473
hsa-miR-197-3p	34.166199	33.1557217	32.674991	34.1486742	32.337021
hsa-miR-199a-3p	31.88044	31.8737029	32.088443	32.9549727	31.304059
hsa-miR-199a-5p	33.862902	34.915155	35.060818	35.1586241	33.366029
hsa-miR-199b-5p	36.535931	35.3257861	ND	ND	36.540729
hsa-miR-19a-3p	25.601015	25.5030072	24.665643	26.0438608	21.323659
hsa-miR-19a-5p	37.874073	ND	37.643318	ND	35.008471
hsa-miR-19b-1-5p	ND	ND	ND	ND	38.220993

hsa-miR-19b-3p	24.822701	24.7201781	23.899716	25.0440148	20.592728
hsa-miR-200a-3p	36.724724	35.7555247	35.062026	35.907241	34.832847
hsa-miR-200b-3p	ND	37.4427085	35.762757	37.3549327	38.04115
hsa-miR-200b-5p	36.728053	ND	ND	ND	ND
hsa-miR-200c-3p	37.189563	35.8130841	35.893999	ND	34.94477
hsa-miR-200c-5p	38.065132	ND	ND	ND	ND
hsa-miR-202-3p	ND	ND	ND	ND	ND
hsa-miR-202-5p	ND	ND	ND	ND	37.613424
hsa-miR-203a	ND	ND	ND	ND	ND
hsa-miR-204-5p	ND	ND	ND	ND	36.064312
hsa-miR-205-5p	31.488778	31.1204508	32.479528	32.8846407	31.498258
hsa-miR-206	35.668954	34.2183947	33.68428	37.7647098	35.743452
hsa-miR-208b	37.622241	ND	38.075393	ND	37.472168
hsa-miR-20a-3p	ND	ND	35.327439	37.1244034	34.763506
hsa-miR-20a-5p	26.058691	25.983674	25.323547	26.3576454	21.676388
hsa-miR-20b-3p	ND	ND	ND	ND	35.292964
hsa-miR-20b-5p	34.589717	33.672805	34.001432	36.1249567	29.993429
hsa-miR-210	31.643031	31.6935649	30.956394	32.0846898	27.659525
hsa-miR-2110	35.440392	34.0015761	33.685234	34.960038	30.770603
hsa-miR-211-5p	ND	ND	ND	ND	ND
hsa-miR-212-3p	36.438873	ND	37.234988	36.6035777	36.030823
hsa-miR-212-5p	ND	ND	ND	ND	ND
hsa-miR-21-3p	37.874403	37.6771984	36.181581	38.9018116	35.45192
hsa-miR-214-3p	37.028769	37.465752	ND	ND	37.149718
hsa-miR-214-5p	ND	37.9950929	ND	35.9394768	ND
hsa-miR-215	31.621062	31.5983873	30.165482	32.0760647	27.183705
hsa-miR-21-5p	25.541775	25.0511376	24.646093	25.6719946	22.647941
hsa-miR-216a-5p	ND	ND	ND	ND	ND
hsa-miR-217	ND	ND	ND	36.8408646	36.674475
hsa-miR-218-2-3p	ND	ND	ND	ND	ND
hsa-miR-218-5p	ND	ND	ND	ND	37.476436
hsa-miR-219-1-3p	ND	ND	ND	ND	ND
hsa-miR-219-5p	ND	ND	ND	38.7626375	35.998593
hsa-miR-221-3p	30.851578	30.60664	31.530541	32.4993571	29.206365
hsa-miR-221-5p	ND	ND	ND	ND	ND
hsa-miR-222-3p	29.118876	28.6113146	28.251192	28.8126775	26.08556
hsa-miR-223-3p	25.765707	25.8140914	26.584904	26.9845658	24.972529
hsa-miR-223-5p	35.574561	34.1508887	34.958092	35.6852552	33.513596
hsa-miR-22-3p	28.925676	28.641235	28.085239	29.8974555	24.816704
hsa-miR-224-3p	ND	ND	ND	ND	ND
hsa-miR-224-5p	ND	ND	ND	ND	ND
hsa-miR-22-5p	32.645183	31.7675451	31.935069	33.5711094	29.575369
hsa-miR-23a-3p	27.702549	27.4393775	27.780687	28.1841295	26.730432
hsa-miR-23a-5p	ND	ND	ND	ND	ND

hsa-miR-23b-3p	30.441004	30.6621191	30.90998	31.0613697	29.475977
hsa-miR-23b-5p	ND	ND	ND	ND	35.985071
hsa-miR-24-1-5p	ND	ND	ND	37.2986103	ND
hsa-miR-24-2-5p	ND	ND	ND	38.0165633	36.906596
hsa-miR-24-3p	28.708645	28.4981861	28.358313	28.9065501	26.263909
hsa-miR-25-3p	27.222345	27.7909332	26.459786	27.0953865	22.861722
hsa-miR-25-5p	ND	36.2010506	35.797975	35.3052106	33.987021
hsa-miR-26a-1-3p	ND	ND	ND	ND	ND
hsa-miR-26a-2-3p	ND	ND	ND	ND	ND
hsa-miR-26a-5p	30.552542	31.0757676	30.567226	31.823176	28.013485
hsa-miR-26b-3p	39.017052	36.2849209	ND	ND	34.7305
hsa-miR-26b-5p	31.242824	30.6864334	29.932473	32.2720518	26.888846
hsa-miR-27a-3p	30.344316	30.6414468	31.127678	ND	29.470473
hsa-miR-27a-5p	36.189367	36.7454644	38.123754	38.2414256	35.90394
hsa-miR-27b-3p	29.983983	30.1681006	30.384419	31.1362561	28.795677
hsa-miR-27b-5p	ND	ND	ND	ND	ND
hsa-miR-28-3p	33.968272	34.7629947	34.91261	35.9601634	32.510313
hsa-miR-28-5p	35.735681	ND	39.143434	37.0408997	36.541343
hsa-miR-296-3p	ND	ND	ND	ND	37.282875
hsa-miR-296-5p	36.663701	33.5820673	ND	35.1098646	31.067566
hsa-miR-299-3p	ND	ND	ND	ND	ND
hsa-miR-299-5p	ND	ND	ND	ND	ND
hsa-miR-29a-3p	30.643075	30.6024027	30.13094	32.2171086	29.44541
hsa-miR-29a-5p	ND	35.7203418	36.680177	37.039082	36.607363
hsa-miR-29b-2-5p	34.965955	35.6578171	35.724994	35.4376635	32.238287
hsa-miR-29b-3p	31.261502	31.5483053	30.827881	32.5696255	27.493311
hsa-miR-29c-3p	29.178502	28.7358549	28.213168	29.9406367	25.586856
hsa-miR-29c-5p	38.727743	ND	35.48184	37.9118406	33.437418
hsa-miR-300	ND	ND	ND	ND	ND
hsa-miR-301a-3p	34.078902	33.6773079	32.364834	33.5444154	28.733142
hsa-miR-301b	ND	38.4665398	ND	ND	34.622326
hsa-miR-302c-5p	ND	ND	ND	ND	ND
hsa-miR-302d-5p	ND	ND	ND	37.1688306	ND
hsa-miR-30a-3p	36.257234	38.1462314	36.200811	35.725075	34.937943
hsa-miR-30a-5p	33.469829	32.7276926	32.228418	33.3454607	31.728539
hsa-miR-30b-5p	31.353911	32.1042833	31.649489	32.5808909	29.370903
hsa-miR-30c-2-3p	ND	ND	ND	ND	ND
hsa-miR-30c-5p	31.693054	32.1215398	31.599117	32.8026622	28.709417
hsa-miR-30d-3p	ND	ND	ND	36.6172348	36.549369
hsa-miR-30d-5p	33.738521	32.9017784	33.139307	34.8894211	29.321012
hsa-miR-30e-3p	36.612801	36.4991071	35.636087	36.7477276	34.493241
hsa-miR-30e-5p	28.99085	28.7507868	28.48249	29.4382868	24.719184
hsa-miR-31-3p	39.53866	ND	ND	ND	ND
hsa-miR-31-5p	ND	ND	36.52583	ND	ND

hsa-miR-320a	28.231623	27.560529	27.653991	29.2696825	24.35483
hsa-miR-320b	29.756923	29.8560668	29.22394	31.1811476	26.470155
hsa-miR-320c	30.587969	30.5955955	30.263211	32.9494097	27.613507
hsa-miR-320d	29.781696	30.2633004	29.952408	30.8775969	26.781328
hsa-miR-323a-3p	ND	36.6822719	ND	ND	35.527776
hsa-miR-32-3p	36.903228	ND	ND	ND	ND
hsa-miR-324-3p	31.328816	31.0899255	30.819063	31.702666	27.252218
hsa-miR-324-5p	34.255019	34.5045718	33.545764	33.9574376	28.735589
hsa-miR-32-5p	31.698013	31.1417369	30.843178	32.8821399	27.813368
hsa-miR-326	37.477357	ND	36.707734	ND	36.54778
hsa-miR-328	37.119042	35.8936487	35.654227	37.1088577	31.551884
hsa-miR-329	35.22773	35.1784243	ND	ND	36.022081
hsa-miR-330-3p	ND	ND	ND	ND	36.768358
hsa-miR-330-5p	ND	ND	ND	ND	ND
hsa-miR-331-3p	36.128131	37.0142362	35.73465	37.1656805	32.027159
hsa-miR-331-5p	ND	ND	ND	ND	ND
hsa-miR-335-3p	35.588234	37.4194964	34.583287	ND	34.146271
hsa-miR-335-5p	33.860988	33.7367468	33.82039	34.8684669	30.688971
hsa-miR-337-3p	ND	36.9949602	ND	37.7011662	35.587508
hsa-miR-337-5p	35.803438	ND	ND	ND	35.464718
hsa-miR-338-3p	33.790605	36.6975951	35.549441	35.1935939	35.309433
hsa-miR-338-5p	ND	ND	38.340225	ND	38.40542
hsa-miR-339-3p	34.171789	35.9696396	35.049956	35.506842	30.901592
hsa-miR-339-5p	ND	ND	ND	ND	35.820029
hsa-miR-33a-3p	ND	ND	ND	ND	36.230297
hsa-miR-33a-5p	36.043508	36.2357495	38.388846	ND	33.424513
hsa-miR-33b-3p	ND	ND	ND	ND	35.962228
hsa-miR-33b-5p	36.920951	36.2161195	34.883209	36.5109762	30.81539
hsa-miR-340-3p	ND	ND	ND	ND	37.738165
hsa-miR-340-5p	ND	ND	ND	ND	36.22462
hsa-miR-342-3p	31.156872	31.4265909	30.984139	32.5616053	29.647503
hsa-miR-342-5p	36.786558	ND	36.122862	35.8167262	36.255941
hsa-miR-345-5p	ND	ND	ND	38.1629063	35.721864
hsa-miR-346	38.415879	37.4074602	ND	ND	ND
hsa-miR-34a-3p	ND	36.8484921	ND	37.4904119	35.798838
hsa-miR-34a-5p	34.342613	32.1902825	31.56366	32.4596418	29.299944
hsa-miR-34b-3p	ND	ND	ND	ND	36.1947
hsa-miR-34c-5p	ND	36.9828752	ND	ND	36.898176
hsa-miR-361-3p	37.177907	34.8701273	ND	36.9466336	31.932026
hsa-miR-361-5p	32.319831	31.9274231	31.587227	32.6494453	29.526745
hsa-miR-362-3p	33.623136	33.1572094	31.908399	33.8518476	29.146553
hsa-miR-362-5p	36.911973	36.5356255	36.927569	38.7721546	36.329141
hsa-miR-363-3p	29.778528	29.6266938	28.77194	30.0404692	25.650868
hsa-miR-363-5p	ND	ND	ND	ND	ND

hsa-miR-365a-3p	36.213329	34.2655383	33.816632	34.5087195	32.87114
hsa-miR-369-3p	ND	ND	ND	ND	37.081004
hsa-miR-369-5p	ND	39.4157145	ND	ND	ND
hsa-miR-370	ND	36.8506503	ND	ND	ND
hsa-miR-373-3p	ND	ND	ND	ND	ND
hsa-miR-373-5p	39.226572	38.6783071	38.347288	ND	37.204456
hsa-miR-374a-5p	33.977478	35.2347299	34.128149	36.8858375	30.731892
hsa-miR-374b-3p	ND	ND	36.173993	ND	36.900601
hsa-miR-374b-5p	35.736613	35.0838977	35.123615	38.8745368	31.969727
hsa-miR-375	33.604754	34.0528971	32.323179	33.1477346	33.040312
hsa-miR-376a-3p	35.853108	36.6713065	36.173418	38.3986955	36.170032
hsa-miR-376a-5p	ND	ND	ND	ND	ND
hsa-miR-376b-3p	36.894269	ND	ND	ND	37.001819
hsa-miR-376c-3p	34.221961	34.5962013	37.062283	36.8948068	34.166225
hsa-miR-377-3p	ND	ND	ND	39.3320452	ND
hsa-miR-377-5p	ND	ND	ND	ND	ND
hsa-miR-378a-3p	30.560682	30.8569512	29.971882	31.1555133	26.927567
hsa-miR-378a-5p	37.284705	35.9535783	36.446158	35.5148833	32.021543
hsa-miR-379-3p	ND	ND	ND	ND	ND
hsa-miR-379-5p	35.903845	ND	ND	ND	36.014845
hsa-miR-380-3p	ND	ND	ND	ND	ND
hsa-miR-381-3p	ND	ND	ND	ND	ND
hsa-miR-382-3p	ND	ND	ND	ND	35.463316
hsa-miR-382-5p	36.8339	34.6866973	37.14442	ND	34.49103
hsa-miR-409-3p	35.09234	ND	ND	36.1074357	36.173384
hsa-miR-409-5p	ND	ND	ND	ND	ND
hsa-miR-410	38.078076	35.5670022	36.859725	ND	35.277878
hsa-miR-411-5p	ND	ND	ND	ND	ND
hsa-miR-412	ND	ND	ND	ND	ND
hsa-miR-421	34.688545	37.1386305	35.796633	35.5521327	31.316737
hsa-miR-423-3p	32.060627	33.3245509	32.171208	32.8958572	26.479322
hsa-miR-423-5p	30.236944	29.7724033	29.263805	30.3485239	25.824181
hsa-miR-424-3p	37.386204	35.1529749	37.668113	37.6168849	32.511209
hsa-miR-424-5p	31.671057	30.5850036	30.819017	33.4795736	28.012741
hsa-miR-425-3p	33.45091	33.5353861	32.847817	34.85699	29.694885
hsa-miR-425-5p	29.161487	29.2782277	28.337651	29.2324458	25.461584
hsa-miR-429	ND	35.155098	ND	39.3680476	37.580174
hsa-miR-431-3p	ND	ND	ND	ND	ND
hsa-miR-431-5p	36.077466	35.5205034	37.028226	ND	ND
hsa-miR-432-3p	ND	ND	ND	ND	ND
hsa-miR-432-5p	37.346879	ND	36.807984	ND	37.47625
hsa-miR-433	ND	ND	ND	ND	ND
hsa-miR-449a	ND	ND	ND	ND	35.464911
hsa-miR-449b-5p	ND	ND	ND	ND	ND

hsa-miR-450a-5p	ND	ND	ND	ND	37.372796
hsa-miR-450b-3p	ND	ND	ND	ND	ND
hsa-miR-450b-5p	ND	35.6315448	ND	ND	35.758197
hsa-miR-451a	20.479308	20.2531744	19.524904	20.6423956	16.786542
hsa-miR-452-5p	36.646427	37.0546408	ND	ND	37.149476
hsa-miR-454-3p	32.863947	33.9376089	33.480458	34.7979749	30.046047
hsa-miR-454-5p	38.288879	ND	ND	38.5446096	35.289922
hsa-miR-455-3p	ND	ND	35.941559	ND	ND
hsa-miR-455-5p	ND	38.1954053	ND	ND	36.934102
hsa-miR-483-3p	36.474362	36.4060961	ND	38.2587797	ND
hsa-miR-483-5p	34.866504	35.8602501	35.921691	36.730837	34.333213
hsa-miR-484	29.778613	29.5859949	28.955147	29.9363052	25.291957
hsa-miR-486-3p	33.986674	34.5093002	33.137967	34.959384	29.485271
hsa-miR-486-5p	25.647598	25.5889486	24.995504	26.0823044	21.12407
hsa-miR-487a	ND	ND	ND	ND	ND
hsa-miR-487b	ND	ND	ND	38.5295689	ND
hsa-miR-489	ND	ND	ND	ND	ND
hsa-miR-490-3p	32.77205	33.0063682	32.950817	34.6736172	32.759603
hsa-miR-490-5p	ND	ND	ND	ND	ND
hsa-miR-491-5p	ND	ND	35.804031	ND	33.110386
hsa-miR-493-3p	ND	ND	ND	ND	ND
hsa-miR-493-5p	ND	ND	ND	ND	36.867464
hsa-miR-494	ND	ND	ND	ND	ND
hsa-miR-495-3p	36.68455	34.2365586	36.591205	35.512672	34.81194
hsa-miR-496	ND	ND	ND	ND	36.586114
hsa-miR-497-5p	33.893224	33.430863	32.983017	34.434466	32.640302
hsa-miR-499a-5p	38.007242	36.0822035	ND	ND	ND
hsa-miR-500a-5p	35.058563	37.0013354	36.687252	ND	32.036053
hsa-miR-501-3p	34.940491	34.5479462	35.317422	36.2960062	31.187409
hsa-miR-501-5p	35.451826	39.1180123	36.089314	ND	32.054644
hsa-miR-502-3p	33.07399	33.2124331	33.033099	34.0895678	28.851638
hsa-miR-502-5p	ND	39.2392738	35.493214	ND	32.887743
hsa-miR-503-5p	ND	ND	ND	ND	35.553888
hsa-miR-505-3p	34.595796	34.2219624	33.149969	34.4733981	30.221114
hsa-miR-505-5p	36.668224	ND	34.619508	39.3138915	32.113221
hsa-miR-507	ND	ND	ND	ND	ND
hsa-miR-508-3p	ND	ND	ND	ND	ND
hsa-miR-509-3-5p	ND	ND	ND	ND	ND
hsa-miR-509-3p	ND	ND	37.119159	ND	ND
hsa-miR-511	ND	ND	36.622462	35.3423595	36.923275
hsa-miR-513a-5p	ND	ND	ND	ND	ND
hsa-miR-514a-3p	ND	ND	ND	ND	ND
hsa-miR-515-3p	ND	ND	ND	ND	ND
hsa-miR-517c-3p	ND	ND	ND	ND	ND

hsa-miR-518d-3p	ND	ND	38.739979	ND	39.045781
hsa-miR-518f-3p	ND	ND	ND	ND	ND
hsa-miR-518f-5p	ND	ND	ND	ND	38.462826
hsa-miR-519b-3p	ND	ND	ND	ND	ND
hsa-miR-520a-5p	ND	ND	ND	ND	39.312289
hsa-miR-520c-3p	ND	ND	ND	ND	ND
hsa-miR-520g	ND	ND	ND	ND	ND
hsa-miR-520h	ND	ND	ND	ND	ND
hsa-miR-524-3p	ND	ND	ND	ND	ND
hsa-miR-525-3p	ND	38.1432588	ND	ND	ND
hsa-miR-525-5p	ND	ND	ND	ND	ND
hsa-miR-532-3p	32.976253	34.7439905	31.984203	33.7697508	28.942405
hsa-miR-532-5p	31.867022	32.1876736	31.019111	32.7052088	27.501901
hsa-miR-539-5p	ND	ND	ND	ND	ND
hsa-miR-542-5p	ND	ND	ND	36.0303114	37.841652
hsa-miR-543	36.018789	ND	35.625996	36.0406541	35.687724
hsa-miR-544a	ND	ND	ND	ND	ND
hsa-miR-545-3p	37.723588	35.9825754	37.083988	ND	34.105899
hsa-miR-548a-3p	37.745525	35.0271757	ND	ND	34.343942
hsa-miR-548a-5p	ND	ND	ND	ND	ND
hsa-miR-548b-3p	ND	ND	ND	ND	ND
hsa-miR-548c-5p	ND	37.6788406	ND	ND	36.022169
hsa-miR-548d-3p	ND	ND	ND	ND	35.931388
hsa-miR-548d-5p	ND	ND	ND	ND	ND
hsa-miR-548e	ND	ND	ND	ND	ND
hsa-miR-548j	ND	ND	ND	ND	38.339469
hsa-miR-548k	ND	ND	ND	ND	35.731176
hsa-miR-548l	ND	ND	ND	ND	37.054876
hsa-miR-548n	ND	ND	ND	ND	ND
hsa-miR-549a	ND	ND	ND	ND	ND
hsa-miR-550a-3p	34.158047	34.5094298	32.997277	34.9539379	29.866405
hsa-miR-550a-5p	35.80666	35.6647216	ND	37.0503312	32.6159
hsa-miR-551a	35.979669	36.7467295	ND	ND	37.180234
hsa-miR-551b-3p	ND	ND	ND	ND	ND
hsa-miR-551b-5p	ND	ND	ND	ND	ND
hsa-miR-556-3p	ND	ND	ND	ND	ND
hsa-miR-564	ND	ND	ND	ND	ND
hsa-miR-570-3p	39.430029	ND	39.211312	36.6089805	34.034401
hsa-miR-571	ND	ND	ND	ND	ND
hsa-miR-573	39.330512	ND	39.3303	ND	ND
hsa-miR-574-3p	33.878877	33.2523433	ND	33.5546687	30.693363
hsa-miR-576-3p	37.186133	36.4956513	37.717765	ND	33.886723
hsa-miR-576-5p	ND	ND	ND	37.835183	ND
hsa-miR-579	37.578002	37.7168071	ND	ND	34.31181

hsa-miR-580	ND	ND	ND	ND	ND
hsa-miR-581	ND	ND	ND	ND	ND
hsa-miR-582-5p	36.713247	ND	36.428375	ND	35.835903
hsa-miR-584-5p	33.853582	34.6536724	34.228424	35.5730032	29.837199
hsa-miR-589-3p	ND	ND	ND	ND	37.143195
hsa-miR-589-5p	ND	37.9573242	37.220023	ND	35.936156
hsa-miR-590-3p	36.255731	ND	36.106165	ND	32.633229
hsa-miR-590-5p	32.451416	32.3375359	31.882115	32.0655431	28.240097
hsa-miR-596	ND	ND	ND	ND	ND
hsa-miR-597	ND	ND	ND	ND	ND
hsa-miR-598	38.435992	35.831321	35.770936	34.2942011	31.612243
hsa-miR-601	ND	ND	ND	ND	ND
hsa-miR-604	ND	ND	ND	ND	ND
hsa-miR-605	ND	ND	ND	ND	ND
hsa-miR-610	ND	ND	ND	ND	35.914447
hsa-miR-612	ND	ND	ND	ND	38.510942
hsa-miR-615-3p	ND	ND	ND	ND	ND
hsa-miR-616-5p	ND	ND	ND	ND	34.31783
hsa-miR-618	36.416439	ND	ND	ND	ND
hsa-miR-621	ND	ND	ND	ND	38.151178
hsa-miR-624-5p	32.043637	31.9949281	32.681318	33.5709828	29.853253
hsa-miR-625-3p	38.699418	36.7686611	ND	37.4536414	33.630751
hsa-miR-626	ND	ND	ND	ND	ND
hsa-miR-627	ND	ND	ND	ND	34.372552
hsa-miR-628-3p	36.773877	ND	36.530131	36.1082703	32.395237
hsa-miR-628-5p	37.926217	37.0980332	ND	ND	36.453716
hsa-miR-629-3p	37.967362	ND	ND	ND	34.4941
hsa-miR-629-5p	33.356167	32.4810272	32.364894	33.5274047	28.674821
hsa-miR-636	36.819476	36.5499539	35.180319	ND	33.12617
hsa-miR-641	ND	ND	ND	ND	ND
hsa-miR-642a-5p	ND	ND	ND	38.9098888	ND
hsa-miR-643	ND	ND	ND	37.6575101	35.74792
hsa-miR-650	ND	ND	ND	ND	ND
hsa-miR-651	36.526436	36.4708674	ND	36.6625205	32.618918
hsa-miR-652-3p	31.667576	30.8748481	30.44817	31.77203	27.232596
hsa-miR-654-3p	37.681423	ND	ND	ND	ND
hsa-miR-654-5p	ND	ND	ND	ND	ND
hsa-miR-655	38.12872	37.2890495	39.155795	37.1405583	38.160053
hsa-miR-659-3p	ND	ND	ND	ND	ND
hsa-miR-660-5p	30.113745	30.3201781	29.270973	30.1489086	26.28892
hsa-miR-662	ND	ND	ND	ND	ND
hsa-miR-663a	ND	36.6248774	36.11074	ND	35.807817
hsa-miR-664a-3p	37.19634	36.805931	38.827704	38.1635496	35.171283
hsa-miR-665	ND	ND	ND	ND	ND

hsa-miR-668	ND	ND	ND	36.7216797	ND
hsa-miR-671-3p	ND	ND	ND	ND	ND
hsa-miR-671-5p	ND	35.8005197	ND	ND	ND
hsa-miR-675-3p	ND	ND	ND	37.1576924	35.099566
hsa-miR-675-5p	ND	ND	ND	ND	ND
hsa-miR-708-3p	37.237868	ND	37.584346	37.6942459	38.407423
hsa-miR-7-1-3p	33.760873	34.696335	34.022076	35.4870703	30.889006
hsa-miR-744-3p	ND	ND	38.816806	ND	ND
hsa-miR-744-5p	36.522169	35.8324141	36.320403	ND	32.82256
hsa-miR-758-3p	ND	ND	ND	ND	ND
hsa-miR-7-5p	33.658871	32.5819559	32.727208	33.7946942	28.995708
hsa-miR-760	ND	ND	ND	ND	ND
hsa-miR-765	ND	ND	ND	ND	ND
hsa-miR-766-3p	35.117362	36.6860482	ND	ND	34.345604
hsa-miR-769-3p	ND	ND	ND	ND	38.728303
hsa-miR-769-5p	35.851707	36.130459	ND	36.787825	33.713088
hsa-miR-770-5p	ND	ND	ND	ND	ND
hsa-miR-873-5p	37.836827	ND	ND	ND	ND
hsa-miR-874	34.549865	34.5564178	ND	34.5004223	31.994025
hsa-miR-876-3p	ND	ND	ND	ND	ND
hsa-miR-877-3p	ND	ND	ND	ND	39.535773
hsa-miR-877-5p	35.13724	36.2863695	34.859498	35.0240157	30.787925
hsa-miR-885-5p	36.827402	37.4100622	33.673771	35.4859882	33.601952
hsa-miR-887	ND	ND	39.545577	37.0592511	39.201102
hsa-miR-888-5p	ND	ND	ND	ND	ND
hsa-miR-889	ND	ND	ND	ND	ND
hsa-miR-92a-1-5p	ND	ND	ND	ND	ND
hsa-miR-92a-3p	25.300832	24.9237326	24.477501	25.5786457	21.588599
hsa-miR-92b-3p	37.995162	ND	36.516378	37.1311122	33.058036
hsa-miR-92b-5p	37.146481	ND	ND	ND	35.879285
hsa-miR-93-3p	34.550474	32.7538145	32.555128	32.8788551	28.468654
hsa-miR-934	37.107013	36.8651582	38.20797	ND	36.87175
hsa-miR-93-5p	27.22075	27.2969072	26.072302	27.2947852	22.509168
hsa-miR-9-3p	ND	ND	37.195329	ND	ND
hsa-miR-940	ND	ND	39.337855	38.2061635	37.095547
hsa-miR-941	ND	ND	35.861323	36.9728219	32.955548
hsa-miR-942	ND	ND	35.071909	38.0682783	32.074166
hsa-miR-95	ND	35.8013268	36.812191	36.7748427	38.138227
hsa-miR-9-5p	ND	ND	ND	ND	ND
hsa-miR-96-5p	34.77288	35.7515523	33.205039	35.6061108	30.232006
hsa-miR-98-5p	35.536257	36.202038	35.645975	36.612563	31.810998
hsa-miR-99a-3p	ND	ND	ND	ND	ND
hsa-miR-99a-5p	32.582048	32.7721345	32.786471	33.7899838	31.874182
hsa-miR-99b-3p	36.561989	ND	ND	36.2597766	ND

hsa-miR-99b-5p	35.30015	33.4356136	34.765927	34.8757118	33.3592
SNORD38B	35.13866	ND	ND	ND	34.473495
SNORD49A	ND	ND	ND	ND	35.074289
UniSp2 CP	19.135186	19.1231893	18.933592	20.1993399	19.174294
UniSp3 IPC	20.681182	20.520274	20.61931	21.0723122	20.613526
UniSp3 IPC	19.908593	19.8274031	19.85471	20.4578827	19.828646
UniSp4 CP	25.554662	25.5058226	25.242512	26.4952997	25.622937
UniSp5 CP	31.214747	31.5617356	30.618685	31.8233333	31.043013
UniSp6 CP	19.292844	18.7783211	19.359054	21.152205	18.903364

Table S1, continued

**Patient ID**	**2561**				
**Dose**	**0 Gy**	**16 Gy**	**26 Gy**	**46 Gy**	**58 Gy**
**Date**	**4/16/2014**	**5/6/2014**	**5/13/2014**	**5/27/2014**	**6/10/2014**
cel-miR-39-3p CP	36.2326837	ND	36.1334668	36.913974	ND
hsa-let-7a-2-3p	ND	ND	ND	ND	ND
hsa-let-7a-3p	33.7585304	35.274353	34.6697935	ND	37.5924319
hsa-let-7a-5p	28.2121451	28.840705	29.0431575	31.9427421	30.1136504
hsa-let-7b-3p	31.8415456	31.710651	32.9115381	ND	35.0681456
hsa-let-7b-5p	24.543053	24.550192	24.9924468	29.5556646	29.2917121
hsa-let-7c	32.9368851	33.158958	32.7985861	36.2470458	33.7969867
hsa-let-7d-3p	27.0569723	27.737499	28.4736859	31.479917	30.6229213
hsa-let-7d-5p	30.0041585	30.733184	31.0760532	35.3825996	33.107184
hsa-let-7e-3p	ND	ND	ND	ND	ND
hsa-let-7e-5p	33.6578642	34.360087	34.4647445	36.5559604	35.7436931
hsa-let-7f-1-3p	34.6485503	34.842	35.6512986	ND	36.6363169
hsa-let-7f-2-3p	37.3918486	36.46986	37.1366584	ND	ND
hsa-let-7f-5p	32.1777974	32.147476	33.6388013	35.864624	34.2111694
hsa-let-7g-3p	33.2121639	32.689178	34.1652743	37.957819	ND
hsa-let-7g-5p	26.0448881	26.470634	26.6677982	30.6702139	30.5934681
hsa-let-7i-3p	34.0842226	35.760936	36.6848262	ND	ND
hsa-let-7i-5p	24.5454385	25.29867	26.0354157	30.2976783	29.8773764
hsa-miR-1	35.1929599	36.941484	ND	37.5957384	ND
hsa-miR-100-5p	30.1530664	31.057618	31.7904115	33.5178626	33.1538313
hsa-miR-101-3p	24.0135397	24.875889	25.4720976	30.1319031	29.1591793
hsa-miR-101-5p	33.2913883	33.713315	36.4484667	37.3364699	ND
hsa-miR-103a-3p	26.0882255	26.96188	26.9035742	31.2758182	30.1789204
hsa-miR-105-3p	ND	ND	ND	ND	ND
hsa-miR-106a-3p	ND	ND	ND	ND	ND
hsa-miR-106a-5p	23.4960264	23.862345	24.5449342	28.8737079	28.4452505
hsa-miR-106b-3p	34.3505444	33.013904	33.8423123	36.4801413	35.2808294
hsa-miR-106b-5p	25.5106197	25.686921	26.0014427	30.9659806	29.8257053
hsa-miR-107	26.3350969	27.027406	27.1672286	31.9918309	31.0376219
hsa-miR-10a-5p	ND	ND	ND	ND	36.604239
hsa-miR-10b-5p	32.2142662	33.319477	32.2200627	33.495641	32.9217781
hsa-miR-1181	ND	ND	ND	ND	ND
hsa-miR-1183	ND	ND	ND	ND	ND
hsa-miR-1185-5p	ND	ND	ND	ND	ND
hsa-miR-1205	ND	ND	ND	ND	ND
hsa-miR-1207-5p	ND	ND	ND	ND	ND
hsa-miR-122-3p	ND	36.493135	36.1496307	ND	ND
hsa-miR-1224-3p	ND	ND	ND	ND	ND
hsa-miR-122-5p	28.736809	26.902134	29.0866039	30.1532947	29.5512878

hsa-miR-1227-3p	36.1449017	ND	37.9400643	ND	ND
hsa-miR-1237-3p	ND	ND	ND	ND	ND
hsa-miR-1238-3p	ND	ND	ND	ND	ND
hsa-miR-1243	ND	ND	ND	ND	ND
hsa-miR-124-3p	36.1834159	35.66159	ND	35.7543549	ND
hsa-miR-1245a	ND	39.996245	38.2688695	ND	39.1671913
hsa-miR-1247-5p	ND	36.569031	36.9564084	ND	ND
hsa-miR-1248	ND	ND	ND	ND	ND
hsa-miR-1249	36.9441119	37.002952	38.3201354	37.0515987	ND
hsa-miR-1254	ND	ND	38.1216391	37.2996984	ND
hsa-miR-1255b-5p	33.992932	35.316795	34.4914835	ND	ND
hsa-miR-1256	ND	ND	ND	ND	ND
hsa-miR-125a-3p	ND	36.90296	ND	ND	ND
hsa-miR-125a-5p	32.6981809	32.510091	33.8537468	33.5239726	33.1616432
hsa-miR-125b-2-3p	ND	35.664718	ND	ND	ND
hsa-miR-125b-5p	30.0656378	30.570195	31.8787505	32.9207063	33.7349517
hsa-miR-1260a	33.7648398	33.435429	33.5187327	35.4363116	33.5870645
hsa-miR-126-3p	25.951508	26.601919	27.0775038	29.5873776	28.2397715
hsa-miR-1270	34.7710929	36.457201	35.8127534	ND	ND
hsa-miR-1271-5p	37.4436028	36.741207	ND	37.7240549	ND
hsa-miR-127-3p	ND	36.211674	ND	ND	ND
hsa-miR-127-5p	ND	ND	ND	ND	ND
hsa-miR-128	27.6041625	28.828651	29.299168	33.6885462	32.7285025
hsa-miR-129-5p	ND	ND	ND	ND	ND
hsa-miR-1296	ND	ND	ND	ND	ND
hsa-miR-130a-3p	27.7687442	28.764599	28.7932915	33.5234577	32.4624304
hsa-miR-130b-3p	30.0144107	31.037278	31.0529059	35.7906688	35.0501369
hsa-miR-130b-5p	36.0923677	35.077497	35.6314914	ND	ND
hsa-miR-132-3p	30.9693392	31.635365	32.9453746	34.5895441	34.801028
hsa-miR-132-5p	ND	ND	ND	ND	35.9641128
hsa-miR-133a	33.9502325	35.72442	35.878953	35.6038946	36.8462092
hsa-miR-133b	34.3562591	36.772204	35.0974516	36.950922	35.6812057
hsa-miR-134	36.8796125	ND	35.8255342	ND	36.5644415
hsa-miR-135a-3p	ND	ND	ND	ND	36.6932131
hsa-miR-135a-5p	37.3848743	35.885775	ND	ND	ND
hsa-miR-135b-5p	ND	ND	ND	ND	ND
hsa-miR-136-3p	ND	36.458845	35.8042242	ND	35.7830487
hsa-miR-136-5p	ND	37.722574	ND	36.7351573	36.2337844
hsa-miR-139-3p	ND	ND	ND	ND	ND
hsa-miR-139-5p	34.8448575	34.558773	36.8676693	36.2371164	35.2911203
hsa-miR-140-3p	23.9781494	24.732461	25.5106392	29.937998	29.3680228
hsa-miR-140-5p	28.4723633	29.695151	31.2614437	35.8743237	34.0010873
hsa-miR-141-3p	34.0252005	34.774466	34.2353062	ND	37.0573406
hsa-miR-141-5p	ND	ND	ND	36.7439754	ND

hsa-miR-142-3p	28.7152314	29.265821	29.5231042	32.0244543	30.2606941
hsa-miR-142-5p	29.9150162	30.364254	30.7240656	33.9742441	33.614292
hsa-miR-143-3p	29.1108086	33.0798	33.1280726	ND	35.5271187
hsa-miR-143-5p	37.1024258	ND	ND	38.2985091	ND
hsa-miR-144-3p	22.8949396	23.837536	23.9305343	29.4580052	28.3062101
hsa-miR-144-5p	29.9423579	30.869437	31.3466591	33.5431609	32.6181922
hsa-miR-145-3p	33.9264001	35.206438	ND	36.4400169	36.4613228
hsa-miR-145-5p	29.204566	31.88021	32.0981621	33.2237456	32.0087967
hsa-miR-1468	38.6572717	36.086937	36.0816752	ND	ND
hsa-miR-146a-5p	29.9584611	30.307274	30.186151	31.964662	31.1345272
hsa-miR-146b-3p	ND	ND	ND	ND	ND
hsa-miR-146b-5p	33.2189769	33.218631	32.8519158	36.6687093	34.7142849
hsa-miR-1471	ND	ND	ND	38.2447245	38.6906676
hsa-miR-147b	ND	38.228506	ND	ND	ND
hsa-miR-148a-3p	27.5458875	28.138582	28.4569759	32.6489157	31.0706675
hsa-miR-148b-3p	26.0923164	26.818318	27.7455131	32.5047204	31.3006425
hsa-miR-148b-5p	ND	39.27404	38.2104782	ND	ND
hsa-miR-149-5p	ND	ND	ND	ND	ND
hsa-miR-150-5p	29.3618489	30.590248	31.0714003	32.4993904	31.4482032
hsa-miR-151a-3p	29.0620971	29.767332	30.7270157	35.0588339	33.2481083
hsa-miR-151a-5p	28.6935959	29.233512	29.7469956	33.5860447	32.6800224
hsa-miR-152	28.9610565	30.025218	30.1827575	34.9633899	32.6452668
hsa-miR-153	36.9572728	ND	ND	ND	ND
hsa-miR-1537	34.0390642	34.99779	35.3752433	ND	36.5059311
hsa-miR-1538	ND	ND	ND	ND	ND
hsa-miR-154-5p	ND	36.835445	ND	36.1238681	ND
hsa-miR-155-5p	35.6222116	ND	37.0406002	35.2631847	ND
hsa-miR-15a-3p	35.0047219	34.614051	35.8557792	ND	ND
hsa-miR-15a-5p	21.1974749	22.093186	22.6558095	27.1669213	26.2922786
hsa-miR-15b-3p	26.8012549	27.472559	28.0784936	32.4980925	32.5889468
hsa-miR-15b-5p	25.4716414	26.174419	26.734478	30.9843884	30.1357027
hsa-miR-16-1-3p	30.4777652	31.007222	32.0394696	34.6244149	33.7675984
hsa-miR-16-2-3p	27.0828153	27.891969	28.5208127	32.8164232	33.1906594
hsa-miR-16-5p	17.1224526	17.912677	18.6211206	22.8509294	22.2522797
hsa-miR-17-3p	32.3201411	32.635171	33.1239996	39.0708217	38.0605429
hsa-miR-17-5p	29.9786008	30.583346	30.5165837	35.221933	34.3578332
hsa-miR-181a-2-3p	ND	38.463036	ND	ND	ND
hsa-miR-181a-3p	ND	ND	ND	ND	ND
hsa-miR-181a-5p	30.9154052	31.267157	31.381334	33.7587744	33.469758
hsa-miR-181b-5p	34.6392669	35.172446	35.5335095	39.39555	36.2108733
hsa-miR-181c-3p	ND	ND	35.9379683	ND	35.7304948
hsa-miR-181c-5p	36.0774739	35.338016	34.5213718	ND	ND
hsa-miR-181d	ND	ND	ND	ND	ND
hsa-miR-182-3p	35.3598178	ND	ND	ND	ND

hsa-miR-182-5p	35.9059502	35.476113	ND	ND	37.4915756
hsa-miR-183-3p	33.9417264	36.150519	35.0775758	ND	ND
hsa-miR-183-5p	32.4055098	32.624456	33.5614871	ND	36.9637661
hsa-miR-184	ND	37.413046	ND	ND	ND
hsa-miR-185-3p	34.9936508	36.89049	37.3018784	ND	ND
hsa-miR-185-5p	23.4214123	24.375214	25.18912	29.9275068	29.1028363
hsa-miR-186-5p	25.2957345	26.055572	26.8186614	32.0071355	30.1323361
hsa-miR-187-3p	ND	ND	ND	ND	ND
hsa-miR-187-5p	ND	ND	37.7588525	ND	ND
hsa-miR-188-3p	36.4618727	37.596618	36.227246	38.0921993	36.8971387
hsa-miR-188-5p	32.204112	33.016135	33.9756667	36.2299712	ND
hsa-miR-18a-3p	29.6519174	30.666002	31.686203	38.1653205	35.1232962
hsa-miR-18a-5p	27.2270628	27.286023	28.4378534	33.0259372	31.8926823
hsa-miR-18b-3p	ND	37.025471	38.2180563	ND	ND
hsa-miR-18b-5p	26.8546724	26.973467	27.7578305	31.9862622	31.1384542
hsa-miR-1908	35.3016945	35.969394	36.6395993	36.941853	38.2967073
hsa-miR-1909-3p	ND	ND	ND	ND	ND
hsa-miR-190a	36.573909	ND	ND	ND	ND
hsa-miR-190b	35.8727173	35.782449	37.7014105	ND	ND
hsa-miR-1912	ND	ND	ND	ND	ND
hsa-miR-1913	ND	ND	ND	ND	37.6962062
hsa-miR-191-3p	36.7395677	ND	ND	ND	ND
hsa-miR-1914-5p	ND	ND	ND	ND	ND
hsa-miR-191-5p	27.3048566	27.948319	28.137699	32.4404037	31.2446469
hsa-miR-192-3p	ND	ND	ND	ND	ND
hsa-miR-192-5p	26.6940561	27.035072	27.8926539	31.2760739	31.1110073
hsa-miR-193a-3p	ND	37.79779	39.1854609	ND	38.4151369
hsa-miR-193a-5p	33.489371	33.476598	34.1268227	37.228576	35.2287414
hsa-miR-193b-3p	35.3378428	33.780714	34.8689496	36.4539718	35.4228091
hsa-miR-193b-5p	36.1003547	ND	ND	36.0329969	35.9859402
hsa-miR-194-3p	ND	ND	ND	ND	ND
hsa-miR-194-5p	27.6501358	28.575278	29.6042508	33.0373238	32.3606677
hsa-miR-195-5p	33.3652918	33.972329	32.6012246	35.4527147	36.8151016
hsa-miR-196a-5p	37.7904393	ND	ND	ND	ND
hsa-miR-196b-3p	36.1604795	34.976846	34.6311651	ND	38.7049105
hsa-miR-196b-5p	32.0806795	33.073625	33.3579241	ND	37.3557791
hsa-miR-1972	34.1260001	34.897792	34.5857791	34.5437382	35.1292409
hsa-miR-197-3p	33.0353805	33.568514	32.8468254	34.2278258	35.8201
hsa-miR-199a-3p	31.7131189	32.602002	31.6250073	34.0261448	32.6541472
hsa-miR-199a-5p	34.8446663	36.571871	34.4449764	ND	ND
hsa-miR-199b-5p	36.8478241	ND	ND	36.4401452	ND
hsa-miR-19a-3p	21.6285129	22.202515	22.7492229	27.5577913	26.8057401
hsa-miR-19a-5p	37.0803341	ND	ND	ND	ND
hsa-miR-19b-1-5p	39.0859427	36.969384	ND	ND	ND

hsa-miR-19b-3p	20.544478	21.213081	22.0281613	26.8931119	26.0676829
hsa-miR-200a-3p	36.1330475	35.912058	34.8242242	ND	36.9031757
hsa-miR-200b-3p	ND	35.856359	37.0655031	39.0224441	37.8793499
hsa-miR-200b-5p	ND	ND	ND	ND	ND
hsa-miR-200c-3p	35.5142588	35.627766	36.1234097	36.7618508	ND
hsa-miR-200c-5p	ND	ND	ND	ND	ND
hsa-miR-202-3p	ND	37.067446	ND	ND	ND
hsa-miR-202-5p	35.9079335	35.549215	ND	ND	ND
hsa-miR-203a	ND	ND	ND	ND	ND
hsa-miR-204-5p	35.3505895	36.55417	ND	36.6105256	ND
hsa-miR-205-5p	35.6586444	33.056171	32.6839241	34.0301177	36.1524924
hsa-miR-206	33.6637591	35.437807	34.5035751	35.6011008	35.5616839
hsa-miR-208b	ND	ND	ND	ND	ND
hsa-miR-20a-3p	35.570111	34.944059	35.0051608	ND	ND
hsa-miR-20a-5p	22.2888326	22.980585	23.6530942	28.1064188	27.5673961
hsa-miR-20b-3p	36.6587513	36.799249	35.95251	ND	ND
hsa-miR-20b-5p	30.712948	31.544569	31.2613623	35.3152651	35.7869714
hsa-miR-210	27.3131322	28.465001	29.2574533	33.4581611	32.7686354
hsa-miR-2110	30.9328223	31.537406	32.6875674	35.6278347	35.9004224
hsa-miR-211-5p	ND	ND	ND	ND	ND
hsa-miR-212-3p	37.325161	ND	ND	ND	ND
hsa-miR-212-5p	ND	ND	ND	ND	ND
hsa-miR-21-3p	35.2550641	36.509132	37.0191897	ND	ND
hsa-miR-214-3p	35.6561655	37.574309	ND	36.2976871	ND
hsa-miR-214-5p	ND	ND	ND	ND	ND
hsa-miR-215	27.5509288	28.037684	28.6008761	32.9415487	32.1299585
hsa-miR-21-5p	22.2962792	23.054682	23.761539	26.9245624	26.0541998
hsa-miR-216a-5p	ND	36.725168	ND	ND	ND
hsa-miR-217	ND	36.260966	ND	ND	ND
hsa-miR-218-2-3p	35.6159331	ND	ND	ND	ND
hsa-miR-218-5p	31.9465366	32.866288	34.041985	37.0808832	37.679385
hsa-miR-219-1-3p	ND	ND	ND	ND	ND
hsa-miR-219-5p	ND	35.882468	37.3375256	ND	ND
hsa-miR-221-3p	30.3715221	31.083854	30.1008835	31.9182691	30.9431684
hsa-miR-221-5p	ND	ND	ND	ND	ND
hsa-miR-222-3p	25.8954323	26.810175	27.5552994	30.1444855	29.7048836
hsa-miR-223-3p	25.620977	25.847315	25.2382565	27.9869585	26.8099543
hsa-miR-223-5p	36.9888724	34.351794	35.5117188	35.4523574	ND
hsa-miR-22-3p	24.7892941	25.524979	26.113334	30.8684562	29.8113704
hsa-miR-224-3p	ND	ND	37.7254464	ND	ND
hsa-miR-224-5p	ND	ND	ND	ND	ND
hsa-miR-22-5p	29.2072343	30.005138	30.348072	34.5994804	33.2749529
hsa-miR-23a-3p	26.7387145	27.291706	27.4442579	28.7420084	28.1864996
hsa-miR-23a-5p	ND	ND	38.4783918	ND	ND

hsa-miR-23b-3p	29.9417792	30.457997	30.6593038	31.9012673	31.0727389
hsa-miR-23b-5p	35.997775	ND	ND	ND	ND
hsa-miR-24-1-5p	ND	ND	ND	ND	ND
hsa-miR-24-2-5p	38.1725992	36.45165	39.1702217	38.1542749	ND
hsa-miR-24-3p	25.889677	26.695683	27.1059906	29.8340432	28.9473046
hsa-miR-25-3p	22.4420534	23.231073	24.1955864	28.5824237	27.9834507
hsa-miR-25-5p	34.5389937	34.961688	35.9063824	ND	ND
hsa-miR-26a-1-3p	ND	36.102241	36.1053338	ND	ND
hsa-miR-26a-2-3p	ND	ND	ND	ND	ND
hsa-miR-26a-5p	29.5486806	29.19337	29.277236	31.9469835	31.0302151
hsa-miR-26b-3p	34.4171269	35.521027	36.0110058	ND	36.2134576
hsa-miR-26b-5p	28.5823781	28.515307	28.2456392	33.1857967	31.8975008
hsa-miR-27a-3p	30.4984857	30.824231	30.2210354	33.0642576	32.175031
hsa-miR-27a-5p	38.1350595	36.943713	36.0909246	36.5559431	36.7646402
hsa-miR-27b-3p	29.4550702	29.343667	29.8810159	31.3596365	30.9765322
hsa-miR-27b-5p	ND	ND	ND	ND	ND
hsa-miR-28-3p	33.8529672	34.025514	33.8746344	38.1346265	36.4257603
hsa-miR-28-5p	37.4923053	37.967376	37.2439142	ND	ND
hsa-miR-296-3p	ND	ND	ND	ND	ND
hsa-miR-296-5p	30.8464599	31.034628	31.4505541	36.1245832	36.5591356
hsa-miR-299-3p	ND	ND	ND	ND	ND
hsa-miR-299-5p	ND	ND	ND	ND	ND
hsa-miR-29a-3p	29.7911582	30.521017	30.7568529	33.2832202	32.4779642
hsa-miR-29a-5p	34.4468946	ND	36.5910375	ND	36.4868754
hsa-miR-29b-2-5p	32.7695819	32.952196	33.2633782	36.1291101	35.8184375
hsa-miR-29b-3p	27.2642445	27.954821	28.8242148	33.841049	33.0108595
hsa-miR-29c-3p	25.6198538	26.290946	27.1152681	31.6366045	30.6048372
hsa-miR-29c-5p	34.1693116	34.050777	35.3736407	35.8619976	37.0440141
hsa-miR-300	ND	ND	ND	ND	ND
hsa-miR-301a-3p	30.3804127	30.149843	30.2933056	34.4664906	34.3573837
hsa-miR-301b	37.7923977	35.360119	35.8804034	ND	36.324752
hsa-miR-302c-5p	ND	ND	37.9069716	ND	ND
hsa-miR-302d-5p	ND	ND	ND	ND	ND
hsa-miR-30a-3p	ND	ND	36.625877	ND	36.3307153
hsa-miR-30a-5p	32.7752322	33.187444	33.239308	35.2142165	35.1123099
hsa-miR-30b-5p	29.849074	30.108595	30.7004589	33.353919	32.1588467
hsa-miR-30c-2-3p	ND	ND	ND	ND	ND
hsa-miR-30c-5p	29.4760071	29.893137	30.2051354	34.0706704	32.6933797
hsa-miR-30d-3p	ND	37.296511	37.803679	ND	ND
hsa-miR-30d-5p	30.1122205	30.568394	31.0831234	35.6843701	34.0174661
hsa-miR-30e-3p	39.1790149	35.714063	36.3258364	36.5962756	36.9214555
hsa-miR-30e-5p	24.6284839	25.486206	26.2306011	30.3522863	29.9896367
hsa-miR-31-3p	ND	ND	ND	ND	ND
hsa-miR-31-5p	ND	ND	ND	38.747648	ND

hsa-miR-320a	24.179348	25.073534	25.3972544	29.4774553	28.6367564
hsa-miR-320b	26.1971565	26.915959	27.5528703	31.8134488	30.8756866
hsa-miR-320c	27.8650182	28.115155	27.8767841	33.4703805	31.959631
hsa-miR-320d	26.6172214	27.470561	27.689321	31.6641702	30.8090296
hsa-miR-323a-3p	39.5720986	ND	ND	ND	ND
hsa-miR-32-3p	38.2375962	ND	ND	ND	ND
hsa-miR-324-3p	26.9816487	27.59487	28.6712072	33.9532662	32.703107
hsa-miR-324-5p	29.2101136	30.308767	31.7677464	35.1363904	35.3800756
hsa-miR-32-5p	27.7888199	28.514246	29.1691967	33.9379658	33.064745
hsa-miR-326	36.0332619	36.555356	34.7313887	ND	36.6900359
hsa-miR-328	31.3012794	32.742756	33.1649778	37.6184795	ND
hsa-miR-329	ND	ND	35.772719	36.7862625	36.4953885
hsa-miR-330-3p	ND	35.935781	ND	ND	ND
hsa-miR-330-5p	ND	ND	ND	ND	ND
hsa-miR-331-3p	33.6924104	34.526997	34.8820887	ND	36.5761183
hsa-miR-331-5p	ND	ND	ND	ND	ND
hsa-miR-335-3p	ND	35.835057	35.6024498	36.4820145	ND
hsa-miR-335-5p	30.8150828	31.750678	31.8377563	35.5590942	34.0736776
hsa-miR-337-3p	37.8710926	ND	35.0691727	35.9886755	39.5438913
hsa-miR-337-5p	ND	37.947656	ND	36.7271359	ND
hsa-miR-338-3p	35.295727	34.726919	34.1337345	36.9297123	35.7123523
hsa-miR-338-5p	ND	ND	ND	ND	38.8941682
hsa-miR-339-3p	31.9414703	31.814939	32.916537	ND	36.1880739
hsa-miR-339-5p	37.2592934	ND	ND	ND	39.0615452
hsa-miR-33a-3p	39.2166017	ND	ND	36.7961648	ND
hsa-miR-33a-5p	35.1840181	35.942668	36.0937525	ND	36.573825
hsa-miR-33b-3p	ND	ND	ND	ND	ND
hsa-miR-33b-5p	32.710709	33.593634	33.8884889	36.1282795	36.4193294
hsa-miR-340-3p	37.8128898	37.148997	ND	ND	ND
hsa-miR-340-5p	38.0567875	37.437589	ND	ND	ND
hsa-miR-342-3p	30.096152	30.81975	31.0331049	33.581555	33.2728066
hsa-miR-342-5p	33.6399004	34.56995	36.7126456	ND	ND
hsa-miR-345-5p	34.2120721	35.790942	ND	ND	ND
hsa-miR-346	ND	ND	37.5825388	39.1890288	ND
hsa-miR-34a-3p	36.8005073	ND	ND	ND	ND
hsa-miR-34a-5p	32.7187803	31.882352	31.8640883	33.4556491	33.8180863
hsa-miR-34b-3p	ND	ND	ND	ND	ND
hsa-miR-34c-5p	ND	ND	ND	ND	ND
hsa-miR-361-3p	32.0604824	33.289621	33.6571781	ND	ND
hsa-miR-361-5p	29.8465185	30.738776	31.222315	33.8947177	32.3318572
hsa-miR-362-3p	29.7161168	30.171755	31.6120028	33.437422	34.9815542
hsa-miR-362-5p	36.2105517	36.765109	ND	38.2328379	37.0107809
hsa-miR-363-3p	25.3252396	26.186308	27.045971	31.4894161	31.086685
hsa-miR-363-5p	ND	ND	ND	ND	ND

hsa-miR-365a-3p	33.5113828	32.634061	33.5703244	37.2753869	34.6729877
hsa-miR-369-3p	ND	ND	ND	ND	ND
hsa-miR-369-5p	ND	ND	ND	ND	ND
hsa-miR-370	ND	36.702808	ND	ND	ND
hsa-miR-373-3p	ND	ND	ND	ND	ND
hsa-miR-373-5p	ND	ND	37.2892558	ND	ND
hsa-miR-374a-5p	33.1910649	32.436322	33.0121254	ND	35.2413104
hsa-miR-374b-3p	37.3661394	ND	37.6333096	ND	ND
hsa-miR-374b-5p	33.9806113	33.667395	33.6820406	36.8499173	35.9337855
hsa-miR-375	34.1684887	32.701463	33.646835	34.1346172	33.1313256
hsa-miR-376a-3p	37.2806842	39.189358	ND	ND	ND
hsa-miR-376a-5p	ND	ND	ND	ND	ND
hsa-miR-376b-3p	ND	ND	ND	ND	ND
hsa-miR-376c-3p	35.852655	35.951682	35.9237266	ND	37.0752325
hsa-miR-377-3p	ND	ND	ND	ND	ND
hsa-miR-377-5p	ND	ND	ND	ND	ND
hsa-miR-378a-3p	27.0706867	27.899915	28.9435915	32.8150514	32.3198349
hsa-miR-378a-5p	33.2470449	33.25516	33.9410855	36.8833747	36.0622563
hsa-miR-379-3p	ND	ND	ND	ND	37.3620959
hsa-miR-379-5p	ND	ND	ND	ND	ND
hsa-miR-380-3p	ND	ND	ND	ND	ND
hsa-miR-381-3p	ND	ND	ND	ND	ND
hsa-miR-382-3p	ND	ND	ND	ND	ND
hsa-miR-382-5p	36.0339554	36.505478	36.5721663	ND	ND
hsa-miR-409-3p	36.4915109	ND	ND	ND	ND
hsa-miR-409-5p	ND	ND	ND	ND	ND
hsa-miR-410	36.5236371	36.278645	ND	37.230254	ND
hsa-miR-411-5p	ND	ND	ND	ND	ND
hsa-miR-412	ND	ND	ND	ND	ND
hsa-miR-421	31.5343852	32.679367	33.0390338	36.5637503	36.5064888
hsa-miR-423-3p	27.3159279	29.350393	30.5811502	34.5086899	33.4690075
hsa-miR-423-5p	25.6454469	26.845426	27.1197002	31.4434287	30.5939588
hsa-miR-424-3p	32.6141478	32.22717	33.8553441	37.4043048	37.6334328
hsa-miR-424-5p	26.7104562	28.156089	28.4851647	32.9274995	31.6219932
hsa-miR-425-3p	29.5155205	30.069911	30.745724	37.0680562	34.2680343
hsa-miR-425-5p	24.7281906	25.517872	26.4332771	31.024659	29.8093659
hsa-miR-429	ND	35.343245	36.5631964	ND	ND
hsa-miR-431-3p	ND	ND	ND	ND	ND
hsa-miR-431-5p	ND	ND	ND	ND	ND
hsa-miR-432-3p	ND	35.984053	ND	ND	ND
hsa-miR-432-5p	ND	ND	ND	ND	37.2539549
hsa-miR-433	ND	ND	ND	ND	ND
hsa-miR-449a	ND	ND	ND	ND	ND
hsa-miR-449b-5p	ND	ND	39.2391863	ND	ND

hsa-miR-450a-5p	37.3891129	37.878235	ND	ND	ND
hsa-miR-450b-3p	ND	ND	ND	ND	ND
hsa-miR-450b-5p	34.4916446	34.180091	35.0870738	ND	ND
hsa-miR-451a	16.3577541	16.955391	17.9713678	22.7621987	22.172958
hsa-miR-452-5p	36.4009011	38.018027	36.1636897	36.8858102	36.66602
hsa-miR-454-3p	32.0720968	31.863934	31.4580041	35.2627968	34.3369531
hsa-miR-454-5p	36.4606455	35.463372	36.0343536	ND	ND
hsa-miR-455-3p	36.2821043	36.170662	ND	ND	ND
hsa-miR-455-5p	37.8602528	37.36443	ND	ND	ND
hsa-miR-483-3p	36.959337	36.429686	36.4256161	36.9564743	35.6343191
hsa-miR-483-5p	34.7334722	34.202091	35.8252864	ND	36.0270057
hsa-miR-484	25.4910733	26.451086	26.9853781	31.7550912	30.9611299
hsa-miR-486-3p	30.0440816	30.71285	31.6365471	36.9594409	34.9216902
hsa-miR-486-5p	21.5810626	22.305229	22.8506149	27.5674767	26.8755891
hsa-miR-487a	ND	ND	ND	ND	ND
hsa-miR-487b	ND	ND	ND	ND	ND
hsa-miR-489	ND	ND	37.1126941	ND	ND
hsa-miR-490-3p	33.7148283	32.869686	32.5205219	33.3151726	32.98027
hsa-miR-490-5p	ND	ND	ND	ND	38.0932906
hsa-miR-491-5p	34.5500079	34.724222	34.5823069	ND	37.1075959
hsa-miR-493-3p	ND	ND	ND	ND	ND
hsa-miR-493-5p	ND	ND	ND	ND	ND
hsa-miR-494	ND	ND	ND	ND	ND
hsa-miR-495-3p	35.5878933	37.279873	35.8559133	34.5308934	34.9590416
hsa-miR-496	ND	ND	ND	38.2689274	38.5508341
hsa-miR-497-5p	33.5092031	33.764914	34.1585494	36.0126394	33.942684
hsa-miR-499a-5p	ND	ND	ND	ND	ND
hsa-miR-500a-5p	34.0534858	33.599227	34.9899364	ND	35.9842224
hsa-miR-501-3p	30.8489648	32.145452	32.9716202	35.5653807	35.7395243
hsa-miR-501-5p	32.1002256	31.603693	33.278202	ND	36.0824164
hsa-miR-502-3p	29.0714374	30.206613	31.1344658	35.5509665	33.8845548
hsa-miR-502-5p	33.8048882	33.878927	35.0899176	ND	ND
hsa-miR-503-5p	ND	37.608175	ND	ND	ND
hsa-miR-505-3p	30.831207	31.513038	33.3315483	35.479413	33.9540696
hsa-miR-505-5p	31.9491518	33.114144	33.6185892	ND	35.4808278
hsa-miR-507	ND	36.255519	ND	ND	ND
hsa-miR-508-3p	ND	ND	ND	ND	ND
hsa-miR-509-3-5p	ND	39.499912	ND	ND	ND
hsa-miR-509-3p	ND	ND	ND	ND	ND
hsa-miR-511	ND	ND	36.7659039	35.5535701	ND
hsa-miR-513a-5p	ND	ND	ND	ND	ND
hsa-miR-514a-3p	ND	ND	ND	ND	ND
hsa-miR-515-3p	ND	ND	ND	ND	ND
hsa-miR-517c-3p	37.7041055	ND	ND	ND	ND

hsa-miR-518d-3p	ND	ND	37.7142042	ND	ND
hsa-miR-518f-3p	ND	ND	ND	ND	ND
hsa-miR-518f-5p	ND	ND	ND	ND	ND
hsa-miR-519b-3p	39.1247919	ND	ND	ND	ND
hsa-miR-520a-5p	ND	ND	ND	ND	ND
hsa-miR-520c-3p	ND	ND	ND	ND	ND
hsa-miR-520g	ND	36.646135	ND	38.4120834	ND
hsa-miR-520h	38.9618227	35.958747	35.067647	ND	35.8280596
hsa-miR-524-3p	ND	ND	ND	ND	ND
hsa-miR-525-3p	ND	ND	ND	ND	ND
hsa-miR-525-5p	ND	ND	ND	ND	ND
hsa-miR-532-3p	29.4504698	30.791689	31.5703756	34.7914184	33.9653179
hsa-miR-532-5p	27.8090737	28.676941	29.2022218	34.1617567	32.7947782
hsa-miR-539-5p	ND	ND	ND	ND	ND
hsa-miR-542-5p	34.9917012	ND	35.9370811	ND	ND
hsa-miR-543	ND	ND	ND	ND	ND
hsa-miR-544a	ND	ND	ND	ND	ND
hsa-miR-545-3p	33.5193624	34.789111	34.2884923	ND	ND
hsa-miR-548a-3p	35.6081672	37.798781	35.4322855	35.8595449	ND
hsa-miR-548a-5p	ND	ND	ND	ND	ND
hsa-miR-548b-3p	ND	ND	ND	ND	ND
hsa-miR-548c-5p	39.18617	ND	37.6601709	39.3546186	35.5447576
hsa-miR-548d-3p	ND	39.507534	ND	ND	ND
hsa-miR-548d-5p	ND	ND	ND	ND	ND
hsa-miR-548e	ND	ND	ND	ND	ND
hsa-miR-548j	34.7267138	ND	ND	ND	ND
hsa-miR-548k	34.9483979	37.775406	ND	ND	ND
hsa-miR-548l	37.4023185	39.089667	38.0644344	37.6701142	ND
hsa-miR-548n	37.104772	36.935404	ND	ND	ND
hsa-miR-549a	ND	ND	ND	ND	ND
hsa-miR-550a-3p	31.2052871	30.687888	30.97619	36.3904607	36.4921893
hsa-miR-550a-5p	33.5695103	33.561825	34.1008115	ND	38.1838786
hsa-miR-551a	36.195374	ND	37.0191568	ND	36.4967383
hsa-miR-551b-3p	ND	ND	36.5722752	ND	ND
hsa-miR-551b-5p	ND	38.265168	ND	ND	ND
hsa-miR-556-3p	ND	ND	ND	ND	ND
hsa-miR-564	ND	ND	ND	ND	38.8732084
hsa-miR-570-3p	34.2124546	34.630718	35.1864662	ND	37.0282761
hsa-miR-571	ND	ND	ND	ND	ND
hsa-miR-573	ND	ND	ND	ND	38.6942164
hsa-miR-574-3p	31.4891788	31.918932	33.5621242	34.0172286	33.8648459
hsa-miR-576-3p	36.4054872	35.500812	36.6971181	ND	37.3417139
hsa-miR-576-5p	33.6409875	ND	ND	ND	ND
hsa-miR-579	34.0632625	34.68471	36.4175475	ND	ND

hsa-miR-580	38.3746403	38.32961	ND	ND	ND
hsa-miR-581	ND	ND	ND	ND	ND
hsa-miR-582-5p	34.7506429	36.576272	34.8338763	36.2884563	36.6150011
hsa-miR-584-5p	31.2301415	32.208403	32.2903641	36.4403438	35.2512081
hsa-miR-589-3p	37.8496919	ND	ND	ND	ND
hsa-miR-589-5p	35.6135114	36.472499	36.6980733	ND	ND
hsa-miR-590-3p	33.928619	33.94176	33.6083565	ND	ND
hsa-miR-590-5p	27.7328511	28.717022	29.7333267	33.4413587	32.680409
hsa-miR-596	ND	ND	ND	ND	ND
hsa-miR-597	ND	38.697115	ND	ND	ND
hsa-miR-598	31.4567962	31.915257	33.3138031	36.1219791	35.5320661
hsa-miR-601	ND	ND	ND	ND	ND
hsa-miR-604	ND	ND	ND	ND	ND
hsa-miR-605	ND	ND	ND	ND	ND
hsa-miR-610	35.2961601	35.778525	ND	ND	ND
hsa-miR-612	ND	ND	ND	ND	ND
hsa-miR-615-3p	ND	ND	ND	ND	ND
hsa-miR-616-5p	35.2406434	36.082119	36.8265215	ND	ND
hsa-miR-618	ND	ND	ND	ND	ND
hsa-miR-621	ND	ND	ND	ND	ND
hsa-miR-624-5p	30.7919933	31.100982	31.8126602	32.8912245	32.617581
hsa-miR-625-3p	34.8599079	36.440846	35.0107651	ND	36.8790383
hsa-miR-626	ND	ND	ND	ND	ND
hsa-miR-627	33.6468778	34.14133	34.0300434	38.2138518	ND
hsa-miR-628-3p	32.9808322	33.59625	33.7856541	ND	35.8695715
hsa-miR-628-5p	ND	37.243941	36.7356695	ND	ND
hsa-miR-629-3p	36.7052537	ND	36.8307448	ND	36.8435591
hsa-miR-629-5p	29.0222651	30.03944	30.7187387	35.3273495	33.2274754
hsa-miR-636	33.698618	33.930476	33.509876	ND	ND
hsa-miR-641	ND	ND	ND	ND	ND
hsa-miR-642a-5p	ND	ND	38.2800769	ND	ND
hsa-miR-643	34.7871326	34.794714	35.1083491	ND	ND
hsa-miR-650	ND	ND	ND	ND	ND
hsa-miR-651	34.8091967	35.129616	36.5452534	ND	36.8852648
hsa-miR-652-3p	27.9155821	28.437664	29.3483143	33.9739435	32.7371805
hsa-miR-654-3p	ND	ND	ND	ND	ND
hsa-miR-654-5p	ND	ND	ND	ND	ND
hsa-miR-655	36.5994874	36.745739	37.428825	37.3295641	37.0990197
hsa-miR-659-3p	ND	ND	ND	ND	ND
hsa-miR-660-5p	26.2393234	26.939881	27.949781	32.2731087	31.5777472
hsa-miR-662	ND	ND	ND	ND	ND
hsa-miR-663a	38.7165222	36.677847	35.2972835	36.5899312	36.9903045
hsa-miR-664a-3p	35.5662913	35.29943	36.4920203	ND	37.7239385
hsa-miR-665	ND	38.715777	ND	ND	ND

hsa-miR-668	ND	ND	ND	ND	ND
hsa-miR-671-3p	ND	ND	ND	ND	36.1661966
hsa-miR-671-5p	38.2761442	38.75137	ND	ND	ND
hsa-miR-675-3p	ND	36.567663	ND	ND	ND
hsa-miR-675-5p	ND	ND	ND	ND	ND
hsa-miR-708-3p	38.4099879	39.094108	35.4525792	36.5074799	36.4978694
hsa-miR-7-1-3p	32.1233642	32.508911	33.583938	35.7601268	34.294712
hsa-miR-744-3p	36.9448911	ND	36.4242998	ND	38.4583116
hsa-miR-744-5p	35.0221401	35.142225	35.779753	39.2299532	37.1840919
hsa-miR-758-3p	ND	ND	ND	ND	ND
hsa-miR-7-5p	28.8249817	29.503841	29.9346933	36.2160482	32.9734841
hsa-miR-760	ND	ND	ND	ND	ND
hsa-miR-765	ND	ND	37.7981722	ND	ND
hsa-miR-766-3p	36.6564539	39.025091	34.5713388	ND	ND
hsa-miR-769-3p	38.379265	38.218219	ND	ND	ND
hsa-miR-769-5p	33.8853647	34.479013	ND	36.2307118	ND
hsa-miR-770-5p	ND	ND	ND	ND	ND
hsa-miR-873-5p	ND	ND	ND	ND	ND
hsa-miR-874	31.5338875	32.607373	33.3149457	35.5177725	34.3511722
hsa-miR-876-3p	ND	ND	ND	ND	ND
hsa-miR-877-3p	ND	38.84483	ND	ND	ND
hsa-miR-877-5p	30.9974426	32.630937	32.6646089	ND	36.5488161
hsa-miR-885-5p	34.8040088	33.922963	34.6950833	37.0817094	34.5555373
hsa-miR-887	ND	ND	ND	ND	ND
hsa-miR-888-5p	ND	ND	ND	ND	ND
hsa-miR-889	ND	ND	ND	ND	ND
hsa-miR-92a-1-5p	ND	ND	ND	ND	ND
hsa-miR-92a-3p	21.2558873	21.991273	22.3592139	26.9131394	26.0872899
hsa-miR-92b-3p	33.5576002	34.287368	33.6923452	ND	39.0968646
hsa-miR-92b-5p	37.6552466	ND	ND	ND	ND
hsa-miR-93-3p	28.8872575	29.551462	30.8651602	34.1485966	33.3391274
hsa-miR-934	37.4181483	37.221357	ND	36.6432012	37.8447796
hsa-miR-93-5p	22.860115	23.527742	24.1092677	28.6398118	27.9957347
hsa-miR-9-3p	36.9102282	ND	ND	ND	ND
hsa-miR-940	ND	ND	37.6462415	ND	38.6969002
hsa-miR-941	34.2243062	34.927129	36.0169621	ND	38.3366881
hsa-miR-942	31.9320479	32.830889	33.1540541	ND	35.8539569
hsa-miR-95	37.2017879	ND	ND	35.3443957	ND
hsa-miR-9-5p	ND	ND	ND	ND	ND
hsa-miR-96-5p	31.8423626	33.011396	32.9708807	36.7196743	36.791944
hsa-miR-98-5p	32.2383147	32.922826	33.6698992	37.4024066	36.7139583
hsa-miR-99a-3p	37.6022921	ND	ND	ND	ND
hsa-miR-99a-5p	31.7697781	32.610641	33.1159893	35.0929563	33.6693326
hsa-miR-99b-3p	35.2486913	ND	ND	ND	ND

hsa-miR-99b-5p	33.6932427	33.761501	33.9961146	35.1350313	33.9430013
SNORD38B	36.4107363	ND	ND	35.8195066	ND
SNORD49A	35.8745761	ND	ND	ND	ND
UniSp2 CP	19.9733407	19.738228	19.6271066	20.123852	19.6158122
UniSp3 IPC	20.9298919	20.745686	20.697461	20.8002446	20.4595701
UniSp3 IPC	20.1202552	19.919577	19.9446152	19.9512006	19.8346994
UniSp4 CP	26.2325482	26.078913	25.7361688	26.198435	25.9463387
UniSp5 CP	31.6814895	31.588513	31.8063914	31.2234293	31.2878806
UniSp6 CP	19.6700496	20.777858	19.0112808	19.7999252	19.071134

Table S1, continued

Patient ID<br>Dose (Gy)<br>Date	Blank
cel-miR-39-3p CP	ND
hsa-let-7a-2-3p	ND
hsa-let-7a-3p	ND
hsa-let-7a-5p	ND
hsa-let-7b-3p	39.308608
hsa-let-7b-5p	ND
hsa-let-7c	ND
hsa-let-7d-3p	ND
hsa-let-7d-5p	ND
hsa-let-7e-3p	ND
hsa-let-7e-5p	ND
hsa-let-7f-1-3p	ND
hsa-let-7f-2-3p	ND
hsa-let-7f-5p	ND
hsa-let-7g-3p	ND
hsa-let-7g-5p	ND
hsa-let-7i-3p	ND
hsa-let-7i-5p	ND
hsa-miR-1	39.166457
hsa-miR-100-5p	ND
hsa-miR-101-3p	ND
hsa-miR-101-5p	ND
hsa-miR-103a-3p	ND
hsa-miR-105-3p	ND
hsa-miR-106a-3p	ND
hsa-miR-106a-5p	ND
hsa-miR-106b-3p	ND
hsa-miR-106b-5p	ND
hsa-miR-107	ND
hsa-miR-10a-5p	ND
hsa-miR-10b-5p	ND
hsa-miR-1181	ND
hsa-miR-1183	ND
hsa-miR-1185-5p	ND
hsa-miR-1205	ND
hsa-miR-1207-5p	ND
hsa-miR-122-3p	ND
hsa-miR-1224-3p	ND
hsa-miR-122-5p	ND

hsa-miR-1227-3p	ND
hsa-miR-1237-3p	ND
hsa-miR-1238-3p	ND
hsa-miR-1243	ND
hsa-miR-124-3p	36.751043
hsa-miR-1245a	39.352928
hsa-miR-1247-5p	ND
hsa-miR-1248	ND
hsa-miR-1249	ND
hsa-miR-1254	ND
hsa-miR-1255b-5p	ND
hsa-miR-1256	ND
hsa-miR-125a-3p	ND
hsa-miR-125a-5p	ND
hsa-miR-125b-2-3p	ND
hsa-miR-125b-5p	ND
hsa-miR-1260a	ND
hsa-miR-126-3p	ND
hsa-miR-1270	ND
hsa-miR-1271-5p	ND
hsa-miR-127-3p	ND
hsa-miR-127-5p	ND
hsa-miR-128	36.11121
hsa-miR-129-5p	ND
hsa-miR-1296	ND
hsa-miR-130a-3p	ND
hsa-miR-130b-3p	ND
hsa-miR-130b-5p	ND
hsa-miR-132-3p	ND
hsa-miR-132-5p	ND
hsa-miR-133a	ND
hsa-miR-133b	ND
hsa-miR-134	37.820339
hsa-miR-135a-3p	ND
hsa-miR-135a-5p	ND
hsa-miR-135b-5p	ND
hsa-miR-136-3p	ND
hsa-miR-136-5p	ND
hsa-miR-139-3p	ND
hsa-miR-139-5p	ND
hsa-miR-140-3p	ND
hsa-miR-140-5p	ND
hsa-miR-141-3p	ND
hsa-miR-141-5p	ND

hsa-miR-142-3p	ND
hsa-miR-142-5p	ND
hsa-miR-143-3p	ND
hsa-miR-143-5p	ND
hsa-miR-144-3p	ND
hsa-miR-144-5p	ND
hsa-miR-145-3p	ND
hsa-miR-145-5p	ND
hsa-miR-1468	ND
hsa-miR-146a-5p	ND
hsa-miR-146b-3p	ND
hsa-miR-146b-5p	ND
hsa-miR-1471	ND
hsa-miR-147b	ND
hsa-miR-148a-3p	ND
hsa-miR-148b-3p	ND
hsa-miR-148b-5p	ND
hsa-miR-149-5p	ND
hsa-miR-150-5p	ND
hsa-miR-151a-3p	ND
hsa-miR-151a-5p	ND
hsa-miR-152	ND
hsa-miR-153	ND
hsa-miR-1537	ND
hsa-miR-1538	ND
hsa-miR-154-5p	ND
hsa-miR-155-5p	ND
hsa-miR-15a-3p	ND
hsa-miR-15a-5p	ND
hsa-miR-15b-3p	ND
hsa-miR-15b-5p	ND
hsa-miR-16-1-3p	ND
hsa-miR-16-2-3p	ND
hsa-miR-16-5p	35.717353
hsa-miR-17-3p	ND
hsa-miR-17-5p	ND
hsa-miR-181a-2-3p	ND
hsa-miR-181a-3p	ND
hsa-miR-181a-5p	ND
hsa-miR-181b-5p	ND
hsa-miR-181c-3p	ND
hsa-miR-181c-5p	ND
hsa-miR-181d	ND
hsa-miR-182-3p	ND

hsa-miR-182-5p	ND
hsa-miR-183-3p	ND
hsa-miR-183-5p	ND
hsa-miR-184	ND
hsa-miR-185-3p	ND
hsa-miR-185-5p	ND
hsa-miR-186-5p	ND
hsa-miR-187-3p	ND
hsa-miR-187-5p	ND
hsa-miR-188-3p	ND
hsa-miR-188-5p	ND
hsa-miR-18a-3p	ND
hsa-miR-18a-5p	ND
hsa-miR-18b-3p	ND
hsa-miR-18b-5p	ND
hsa-miR-1908	ND
hsa-miR-1909-3p	ND
hsa-miR-190a	ND
hsa-miR-190b	ND
hsa-miR-1912	ND
hsa-miR-1913	ND
hsa-miR-191-3p	ND
hsa-miR-1914-5p	ND
hsa-miR-191-5p	ND
hsa-miR-192-3p	ND
hsa-miR-192-5p	ND
hsa-miR-193a-3p	ND
hsa-miR-193a-5p	ND
hsa-miR-193b-3p	ND
hsa-miR-193b-5p	ND
hsa-miR-194-3p	39.312087
hsa-miR-194-5p	ND
hsa-miR-195-5p	ND
hsa-miR-196a-5p	ND
hsa-miR-196b-3p	ND
hsa-miR-196b-5p	ND
hsa-miR-1972	ND
hsa-miR-197-3p	ND
hsa-miR-199a-3p	ND
hsa-miR-199a-5p	ND
hsa-miR-199b-5p	ND
hsa-miR-19a-3p	ND
hsa-miR-19a-5p	ND
hsa-miR-19b-1-5p	ND

hsa-miR-19b-3p	ND
hsa-miR-200a-3p	ND
hsa-miR-200b-3p	ND
hsa-miR-200b-5p	ND
hsa-miR-200c-3p	ND
hsa-miR-200c-5p	ND
hsa-miR-202-3p	ND
hsa-miR-202-5p	ND
hsa-miR-203a	ND
hsa-miR-204-5p	ND
hsa-miR-205-5p	ND
hsa-miR-206	36.816888
hsa-miR-208b	ND
hsa-miR-20a-3p	ND
hsa-miR-20a-5p	ND
hsa-miR-20b-3p	ND
hsa-miR-20b-5p	ND
hsa-miR-210	ND
hsa-miR-2110	37.402998
hsa-miR-211-5p	ND
hsa-miR-212-3p	ND
hsa-miR-212-5p	ND
hsa-miR-21-3p	ND
hsa-miR-214-3p	ND
hsa-miR-214-5p	ND
hsa-miR-215	ND
hsa-miR-21-5p	37.138882
hsa-miR-216a-5p	ND
hsa-miR-217	ND
hsa-miR-218-2-3p	ND
hsa-miR-218-5p	ND
hsa-miR-219-1-3p	ND
hsa-miR-219-5p	ND
hsa-miR-221-3p	ND
hsa-miR-221-5p	ND
hsa-miR-222-3p	ND
hsa-miR-223-3p	ND
hsa-miR-223-5p	ND
hsa-miR-22-3p	ND
hsa-miR-224-3p	ND
hsa-miR-224-5p	ND
hsa-miR-22-5p	ND
hsa-miR-23a-3p	ND
hsa-miR-23a-5p	ND

hsa-miR-23b-3p	ND
hsa-miR-23b-5p	ND
hsa-miR-24-1-5p	ND
hsa-miR-24-2-5p	ND
hsa-miR-24-3p	36.312814
hsa-miR-25-3p	ND
hsa-miR-25-5p	ND
hsa-miR-26a-1-3p	ND
hsa-miR-26a-2-3p	ND
hsa-miR-26a-5p	35.137993
hsa-miR-26b-3p	ND
hsa-miR-26b-5p	ND
hsa-miR-27a-3p	ND
hsa-miR-27a-5p	37.006221
hsa-miR-27b-3p	ND
hsa-miR-27b-5p	ND
hsa-miR-28-3p	ND
hsa-miR-28-5p	ND
hsa-miR-296-3p	ND
hsa-miR-296-5p	ND
hsa-miR-299-3p	ND
hsa-miR-299-5p	ND
hsa-miR-29a-3p	ND
hsa-miR-29a-5p	ND
hsa-miR-29b-2-5p	ND
hsa-miR-29b-3p	ND
hsa-miR-29c-3p	ND
hsa-miR-29c-5p	ND
hsa-miR-300	ND
hsa-miR-301a-3p	ND
hsa-miR-301b	ND
hsa-miR-302c-5p	ND
hsa-miR-302d-5p	ND
hsa-miR-30a-3p	ND
hsa-miR-30a-5p	ND
hsa-miR-30b-5p	ND
hsa-miR-30c-2-3p	ND
hsa-miR-30c-5p	ND
hsa-miR-30d-3p	ND
hsa-miR-30d-5p	ND
hsa-miR-30e-3p	ND
hsa-miR-30e-5p	ND
hsa-miR-31-3p	ND
hsa-miR-31-5p	ND

hsa-miR-320a	ND
hsa-miR-320b	ND
hsa-miR-320c	ND
hsa-miR-320d	ND
hsa-miR-323a-3p	ND
hsa-miR-32-3p	ND
hsa-miR-324-3p	37.870118
hsa-miR-324-5p	ND
hsa-miR-32-5p	ND
hsa-miR-326	ND
hsa-miR-328	ND
hsa-miR-329	ND
hsa-miR-330-3p	ND
hsa-miR-330-5p	ND
hsa-miR-331-3p	ND
hsa-miR-331-5p	ND
hsa-miR-335-3p	ND
hsa-miR-335-5p	ND
hsa-miR-337-3p	ND
hsa-miR-337-5p	ND
hsa-miR-338-3p	ND
hsa-miR-338-5p	ND
hsa-miR-339-3p	ND
hsa-miR-339-5p	ND
hsa-miR-33a-3p	ND
hsa-miR-33a-5p	ND
hsa-miR-33b-3p	ND
hsa-miR-33b-5p	ND
hsa-miR-340-3p	ND
hsa-miR-340-5p	ND
hsa-miR-342-3p	ND
hsa-miR-342-5p	ND
hsa-miR-345-5p	ND
hsa-miR-346	ND
hsa-miR-34a-3p	ND
hsa-miR-34a-5p	ND
hsa-miR-34b-3p	ND
hsa-miR-34c-5p	ND
hsa-miR-361-3p	ND
hsa-miR-361-5p	ND
hsa-miR-362-3p	ND
hsa-miR-362-5p	38.337503
hsa-miR-363-3p	ND
hsa-miR-363-5p	ND

hsa-miR-365a-3p	ND
hsa-miR-369-3p	ND
hsa-miR-369-5p	ND
hsa-miR-370	ND
hsa-miR-373-3p	ND
hsa-miR-373-5p	ND
hsa-miR-374a-5p	ND
hsa-miR-374b-3p	ND
hsa-miR-374b-5p	ND
hsa-miR-375	ND
hsa-miR-376a-3p	ND
hsa-miR-376a-5p	ND
hsa-miR-376b-3p	ND
hsa-miR-376c-3p	37.684538
hsa-miR-377-3p	ND
hsa-miR-377-5p	ND
hsa-miR-378a-3p	ND
hsa-miR-378a-5p	ND
hsa-miR-379-3p	ND
hsa-miR-379-5p	ND
hsa-miR-380-3p	ND
hsa-miR-381-3p	ND
hsa-miR-382-3p	ND
hsa-miR-382-5p	ND
hsa-miR-409-3p	ND
hsa-miR-409-5p	ND
hsa-miR-410	ND
hsa-miR-411-5p	ND
hsa-miR-412	ND
hsa-miR-421	ND
hsa-miR-423-3p	ND
hsa-miR-423-5p	ND
hsa-miR-424-3p	ND
hsa-miR-424-5p	ND
hsa-miR-425-3p	ND
hsa-miR-425-5p	ND
hsa-miR-429	ND
hsa-miR-431-3p	ND
hsa-miR-431-5p	ND
hsa-miR-432-3p	ND
hsa-miR-432-5p	ND
hsa-miR-433	ND
hsa-miR-449a	ND
hsa-miR-449b-5p	ND

hsa-miR-450a-5p	ND
hsa-miR-450b-3p	ND
hsa-miR-450b-5p	ND
hsa-miR-451a	ND
hsa-miR-452-5p	ND
hsa-miR-454-3p	ND
hsa-miR-454-5p	39.606464
hsa-miR-455-3p	ND
hsa-miR-455-5p	ND
hsa-miR-483-3p	ND
hsa-miR-483-5p	ND
hsa-miR-484	ND
hsa-miR-486-3p	ND
hsa-miR-486-5p	ND
hsa-miR-487a	ND
hsa-miR-487b	ND
hsa-miR-489	ND
hsa-miR-490-3p	33.170087
hsa-miR-490-5p	ND
hsa-miR-491-5p	37.441805
hsa-miR-493-3p	ND
hsa-miR-493-5p	ND
hsa-miR-494	ND
hsa-miR-495-3p	36.468938
hsa-miR-496	ND
hsa-miR-497-5p	ND
hsa-miR-499a-5p	ND
hsa-miR-500a-5p	ND
hsa-miR-501-3p	ND
hsa-miR-501-5p	ND
hsa-miR-502-3p	ND
hsa-miR-502-5p	ND
hsa-miR-503-5p	37.440491
hsa-miR-505-3p	ND
hsa-miR-505-5p	36.709847
hsa-miR-507	ND
hsa-miR-508-3p	ND
hsa-miR-509-3-5p	ND
hsa-miR-509-3p	ND
hsa-miR-511	ND
hsa-miR-513a-5p	ND
hsa-miR-514a-3p	ND
hsa-miR-515-3p	ND
hsa-miR-517c-3p	ND

hsa-miR-518d-3p	ND
hsa-miR-518f-3p	ND
hsa-miR-518f-5p	ND
hsa-miR-519b-3p	ND
hsa-miR-520a-5p	ND
hsa-miR-520c-3p	ND
hsa-miR-520g	ND
hsa-miR-520h	ND
hsa-miR-524-3p	ND
hsa-miR-525-3p	ND
hsa-miR-525-5p	ND
hsa-miR-532-3p	ND
hsa-miR-532-5p	ND
hsa-miR-539-5p	ND
hsa-miR-542-5p	ND
hsa-miR-543	ND
hsa-miR-544a	ND
hsa-miR-545-3p	ND
hsa-miR-548a-3p	ND
hsa-miR-548a-5p	ND
hsa-miR-548b-3p	ND
hsa-miR-548c-5p	ND
hsa-miR-548d-3p	ND
hsa-miR-548d-5p	39.348751
hsa-miR-548e	ND
hsa-miR-548j	ND
hsa-miR-548k	ND
hsa-miR-548l	ND
hsa-miR-548n	ND
hsa-miR-549a	ND
hsa-miR-550a-3p	ND
hsa-miR-550a-5p	ND
hsa-miR-551a	ND
hsa-miR-551b-3p	ND
hsa-miR-551b-5p	ND
hsa-miR-556-3p	ND
hsa-miR-564	ND
hsa-miR-570-3p	ND
hsa-miR-571	ND
hsa-miR-573	ND
hsa-miR-574-3p	ND
hsa-miR-576-3p	ND
hsa-miR-576-5p	ND
hsa-miR-579	ND

hsa-miR-580	ND
hsa-miR-581	ND
hsa-miR-582-5p	ND
hsa-miR-584-5p	ND
hsa-miR-589-3p	ND
hsa-miR-589-5p	ND
hsa-miR-590-3p	ND
hsa-miR-590-5p	ND
hsa-miR-596	ND
hsa-miR-597	ND
hsa-miR-598	ND
hsa-miR-601	ND
hsa-miR-604	37.690787
hsa-miR-605	ND
hsa-miR-610	ND
hsa-miR-612	ND
hsa-miR-615-3p	ND
hsa-miR-616-5p	ND
hsa-miR-618	ND
hsa-miR-621	ND
hsa-miR-624-5p	33.103223
hsa-miR-625-3p	ND
hsa-miR-626	ND
hsa-miR-627	ND
hsa-miR-628-3p	ND
hsa-miR-628-5p	ND
hsa-miR-629-3p	38.370635
hsa-miR-629-5p	ND
hsa-miR-636	ND
hsa-miR-641	ND
hsa-miR-642a-5p	ND
hsa-miR-643	ND
hsa-miR-650	ND
hsa-miR-651	ND
hsa-miR-652-3p	ND
hsa-miR-654-3p	ND
hsa-miR-654-5p	ND
hsa-miR-655	37.129818
hsa-miR-659-3p	ND
hsa-miR-660-5p	ND
hsa-miR-662	ND
hsa-miR-663a	ND
hsa-miR-664a-3p	ND
hsa-miR-665	ND

hsa-miR-668	ND
hsa-miR-671-3p	ND
hsa-miR-671-5p	ND
hsa-miR-675-3p	ND
hsa-miR-675-5p	ND
hsa-miR-708-3p	ND
hsa-miR-7-1-3p	ND
hsa-miR-744-3p	37.006375
hsa-miR-744-5p	ND
hsa-miR-758-3p	ND
hsa-miR-7-5p	ND
hsa-miR-760	ND
hsa-miR-765	ND
hsa-miR-766-3p	ND
hsa-miR-769-3p	ND
hsa-miR-769-5p	ND
hsa-miR-770-5p	ND
hsa-miR-873-5p	ND
hsa-miR-874	ND
hsa-miR-876-3p	ND
hsa-miR-877-3p	ND
hsa-miR-877-5p	ND
hsa-miR-885-5p	ND
hsa-miR-887	ND
hsa-miR-888-5p	ND
hsa-miR-889	ND
hsa-miR-92a-1-5p	ND
hsa-miR-92a-3p	ND
hsa-miR-92b-3p	ND
hsa-miR-92b-5p	ND
hsa-miR-93-3p	ND
hsa-miR-934	ND
hsa-miR-93-5p	ND
hsa-miR-9-3p	ND
hsa-miR-940	ND
hsa-miR-941	ND
hsa-miR-942	ND
hsa-miR-95	ND
hsa-miR-9-5p	ND
hsa-miR-96-5p	ND
hsa-miR-98-5p	ND
hsa-miR-99a-3p	ND
hsa-miR-99a-5p	ND
hsa-miR-99b-3p	ND

hsa-miR-99b-5p	ND
SNORD38B	34.979
SNORD49A	ND
UniSp2 CP	21.030131
UniSp3 IPC	20.882032
UniSp3 IPC	20.133902
UniSp4 CP	27.562075
UniSp5 CP	32.167869
UniSp6 CP	20.102563

Table S2. RNA samples measurements.

Profiling cohort

Patient	Dose	A_{260}/A_{280}	ng/µl
2468	0 Gy	1.69	10.5
	2 Gy	1.47	12.0
	22 Gy	2.23	8.6
	60 Gy	2.08	9.0
2510	0 Gy	1.78	12.0
	28 Gy	1.81	9.6
	42 Gy	1.8	13.2
	54 Gy	1.57	16.4
2526	0 Gy	1.84	12.4
	10 Gy	1.57	14.3
	30 Gy	1.63	13.8
	46 Gy	1.62	11.4
2534	0 Gy	1.67	10.9
	10 Gy	1.61	11.3
	28 Gy	1.73	12.5
	38 Gy	1.69	11.3
	62 Gy	2.03	13.1
2561	26 Gy	1.79	12.3
	46 Gy	1.61	12.0
	58 Gy	1.61	14.8

Validation Cohort

Patient	Dose	ng/µl
AE1	0 Gy	17.5
	20 Gy	13.5
	40 Gy	9.5
CG1	0 Gy	12
	20 Gy	7.8
	40 Gy	10
GH1	0 Gy	10
	20 Gy	10
	40 Gy	16
GW1	0 Gy	8
	20 Gy	16
	40 Gy	10
KA1	0 Gy	9
	20 Gy	12
	40 Gy	12.5

Patient	Dose	ng/µl
KA2	0 Gy	8
	20 Gy	12
	40 Gy	11
MS1	0 Gy	10
	20 Gy	12
	40 Gy	8
PB1	0 Gy	10
	20 Gy	16
	40 Gy	16
PJ1	0 Gy	20
	20 Gy	15
	40 Gy	10
WJ1	0 Gy	10
	20 Gy	11
	40 Gy	24.5

Validation Cohort, continued

Patient	Dose	A_{260}/A_{280}	ng/µl
BE1	0 Gy	1.31	14.7
	20 Gy	1.17	13.7
	40 Gy	1.33	14.3
DW1	0 Gy	1.39	15.0
	20 Gy	1.26	18.6
	40 Gy	1.23	9.8
FL1	0 Gy	1.47	11.2
	20 Gy	1.37	11.2
	40 Gy	1.28	9.4
GK1	0 Gy	1.52	9.9
	20 Gy	1.37	12.3
	40 Gy	1.40	12.7
KI1	0 Gy	1.48	10.1
	20 Gy	1.61	8.0
	40 Gy	1.25	10.3
KM1	0 Gy	1.32	11.0
	20 Gy	1.57	11.4
	40 Gy	1.35	10.1

Patient	Dose	A_{260}/A_{280}	ng/µl
LJ1	0 Gy	1.57	10.2
	20 Gy	1.48	11.8
	40 Gy	1.51	10.9
SB1	0 Gy	1.06	10.9
	20 Gy	1.35	16.5
	40 Gy	1.18	17.3
SJ1	0 Gy	1.61	12.9
	20 Gy	1.46	8.1
	40 Gy	1.62	8.5
SW1	0 Gy	1.59	10.3
	20 Gy	1.67	10.0
	40 Gy	1.68	7.7
ZA1	0 Gy	1.47	11.9
	20 Gy	1.33	12.1
	40 Gy	1.35	14.8

Cell Lines

Line	Day	#	A_{260}/A_{280}	ng/µl
A549	Day 1	1	1.84	24
		2	1.86	26.5
		3	1.89	23.2
		4	2.13	23.7
	Day 2	1	1.93	50.9
		2	1.96	51.3
		3	1.93	54.0
		4	1.95	41.3
	Day 3	1	1.98	93.5
		2	1.98	105.2
		3	1.98	114.5
		4	2.10	99.1

Line	Dose	#	A_{260}/A_{280}	ng/µl
A549	2 Gy x 1	1	1.89	24.7
		2	1.92	22.6
		3	1.83	22.9
		4	1.88	21.3
	2 Gy x 2	1	1.95	46.2
		2	1.87	45.0
		3	1.89	29.4
		4	1.90	46.2
	2 Gy x 3	1	1.99	77.1
		2	1.99	77.1
		3	1.99	65.1
		4	1.98	92.0

Cell Lines, continued

Line	Day	#	A_{260}/A_{280}	ng/µl
NCI-H460	Day 1	1	1.85	103.5
		2	1.99	89.2
		3	1.98	103.8
	Day 3	1	2.00	87.9
		2	1.98	79.4
		3	1.97	68.7
	Day 6	1	1.97	85.2
		2	2.04	83.9
		3	1.99	93.9

Line	Dose	#	A_{260}/A_{280}	ng/µl
NCI-H460	2 Gy x 2	1	1.97	88.6
		2	1.89	95.6
		3	2.0	98.0
	2 Gy x 5	1	1.94	84.9
		2	1.99	88.9
		3	1.96	70.2

Line	Day	#	A_{260}/A_{280}	ng/µl
NCI-H 1299	Day 1	1	1.97	37.7
		2	1.97	36.6
		3	2.04	43.8
		4	2.02	36.6
	Day 2	1	2.01	69.2
		2	2.02	98.8
		3	2.02	93.0
		4	2.00	89.4
	Day 3	1	2.04	183.3
		2	2.04	250.5
		3	2.03	139.8
		4	2.02	96.5

Line	Dose	#	A_{260}/A_{280}	ng/µl
A549	2 Gy x 1	1	2.04	47.3
		2	2.00	42.3
		3	2.00	42.8
		4	2.01	43.2
	2 Gy x 2	1	2.05	71.2
		2	2.02	113.4
		3	2.00	70.3
		4	2.02	68.0
	2 Gy x 3	1	2.03	85.2
		2	2.05	75.4
		3	2.04	161.2
		4	2.04	78.6

Line	Day	#	A_{260}/A_{280}	ng/µl
IMR-90	Day 1	1	1.96	19.8
		2	1.98	22.7
		3	1.91	18.5
		4	1.95	23.8
	Day 3	1	1.98	31.7
		2	1.92	35.5
		3	1.94	29.7
		4	1.92	31.7

Line	Dose	#	A_{260}/A_{280}	ng/µl
IMR-90	2 Gy x 1	1	1.94	24.1
		2	1.94	21.2
		3	1.89	26.1
		4	1.80	22.6
	2 Gy x 3	1	1.88	29.2
		2	2.04	23.7
		3	1.89	24.4
		4	1.94	24.1

Cell Lines, continued

Line	Day	#	A_{260}/A_{280}	ng/µl
MRC5	Day 1	1	1.97	22.0
		2	1.89	24.2
		3	2.02	24.0
		4	1.93	24.6
	Day 3	1	1.98	42.9
		2	1.96	44.3
		3	1.98	39.1
		4	2.03	52.9

Line	Dose	#	A_{260}/A_{280}	ng/µl
MRC5	2 Gy x 1	1	2.01	23.8
		2	1.98	23.7
		3	2.02	20.4
		4	1.87	24.7
	2 Gy x 3	1	2.00	32.6
		2	1.96	38.4
		3	2.05	32.8
		4	2.03	36.1

Table S3. Validation cohort Q-PCR data. dCp values are provided.

Dose 0 Gy

Patient ID	29a-3p	150-5p	101-5p	342-3p	30d-5p	320a	142-3p	191-5p	125b-5p	15b-5p
AE1	1.3566	2.3198	-1.1845	1.6911	2.9983	4.6315	4.7946	2.8429	1.3550	2.0694
BE1	0.1940	4.0895		3.1424	4.4393	5.7702	4.4499	2.0597	1.6511	3.7291
CG1	1.0422	0.5213	-0.1859	1.6896	1.5509	5.3810	2.6789	1.6400		1.1540
DW1	-0.2043	1.2334	-2.8507	0.3049	1.8342	4.0968	2.4214	1.4259	-1.7831	1.5811
FL1	1.8458	4.3498	0.2903	1.7656	3.4612	5.2786	5.4355	3.2215	1.9018	3.8248
GH1	0.3586	2.1021	-1.1871	1.1845	0.7506	3.6755	4.1122	1.9736	-0.6112	1.8976
GK1	2.0040	4.9059	-1.5191	3.3807	4.2163	5.8128	5.7310	3.8147	2.2965	2.9092
GW1	1.0399	2.1855		0.8381	1.8543	3.5213	5.0791	2.4615	0.8257	2.0778
KA1	0.8924	3.3252	1.3666	1.9713	1.7591	3.3626	5.4453	2.7854	2.9060	3.3920
KA2	1.9668	4.7617	-0.2480	3.4382	2.7980	4.3828	6.5583	3.3432	2.1531	3.7775
KI1	-0.1856	4.2458		3.4129	3.5184	5.5703	4.8812	2.7323	1.6853	3.8912
KM1	-1.1860	1.9558		0.6906	2.1394	3.3388	3.6699	1.3822	0.3845	0.5839
LJ1	2.6564	2.0793	-0.4766	1.8171	3.5908	4.5418	5.7142	4.0735	2.1899	3.7099
MS1	2.0878	2.9821	-1.9403	2.1870	3.9484	3.6437	6.2386	3.9710	1.5795	5.6705
PB1	1.5276	1.3093	-0.3386	0.5002	0.7851	3.3792	3.0916	1.6418	0.8607	0.6243
PJ1	2.7579	4.2149	-3.3252	1.6742	2.4587	2.5957	5.1129	2.7855	1.1460	3.8614
SB1	1.3149	2.2492	-2.4078	0.4116	2.3897	4.8789	4.6979	1.8981	0.9606	2.3981
SJ1	-0.0608	0.9654		1.3613	3.5326	3.2269	3.9981	2.5862	1.2496	3.3230
SW1	0.5280	4.2147		2.7481	3.2703	4.1385	5.0694	3.3559	0.5460	3.1847
WJ1	2.9026	4.0377	-0.8774	2.1990	3.3777	3.7583	6.3636	3.7822	1.7377	4.0027
ZA1	1.5170	3.0294	-1.9390	1.9178	3.3347	4.0103	6.1316	3.6374	1.2030	4.1343

Dose 20 Gy

Patient ID	29a-3p	150-5p	101-5p	342-3p	30d-5p	320a	142-3p	191-5p	125b-5p	15b-5p
AE1	0.7885	0.9333	-2.6989	0.4350	2.3704	3.7191	4.4036	1.8382	1.2456	0.4728
BE1	-0.1755	1.5990	-2.0923	1.2409	3.9442	4.7140	4.3722	2.9093	2.0324	3.5771
CG1	-0.1210	1.8387	-1.0649	1.0083	2.7382	3.0522	4.1611	2.2107	-0.0399	2.8351
DW1	0.0642	2.7315	-3.0736	2.0982	3.8435	4.3587	6.1530	4.0307	0.4326	4.0349
FL1	-0.2905	3.2314		2.1877	4.1067	5.0200	6.1275	3.7068	1.9833	4.3627
GH1	0.9244	3.2915		1.0215	2.3278	2.9604	5.0689	2.3186	1.5169	3.6018
GK1	1.6190	5.0292	1.4705	3.2530	5.7541	7.3249	5.8399	5.6885	1.4067	4.9893
GW1	0.6677	0.7233		0.1667	1.9421	4.5589	3.1367	1.9900	1.1631	1.8560
KA1	-0.1004	1.4467	0.6139	0.9017	1.5098	4.5207	4.8143	1.3418	0.5963	1.4250
KA2	0.6985	2.6688		2.2927	2.1051	3.6814	4.8165	2.4770	1.7460	2.8143
KI1										
KM1	-0.3386	1.7005	-2.5413	0.4864	1.3208	3.9671	4.4570	2.1188	0.1011	2.4853
LJ1	-0.7196	1.3829		1.8276	2.8228	4.5139	4.8880	3.5609	1.8818	2.4814
MS1	0.8301	-0.3799	-2.5228	0.3506	2.8114	2.6941	4.0474	2.5017	1.4752	3.9327
PB1	0.0239	1.0583	-2.4954	-1.2865	-2.5279	3.2954	2.8020	0.6825	-0.1292	0.3201
PJ1	0.6160	3.1028		1.2156	2.4683	3.8632	5.8833	2.7693	0.6119	3.6933
SB1	0.1083	1.1876	-4.5234	0.5775	2.4638	5.3869	4.6565	2.0317	-0.1113	2.2819
SJ1	0.4226	1.7759	-2.9887	0.7248	2.6400	3.2493	4.0769	1.8399	0.1462	2.2178
SW1	0.7832	1.9142		2.3704	2.6392	3.7403	4.6524	2.8354		3.1385
WJ1	1.4155	2.6871	-1.8764	1.2462	3.2972	3.7079	5.2983	2.3378	1.5448	3.1703
ZA1	1.4084	1.7448	-2.4281	0.8224	2.8823	3.5389	3.8866	1.8023	0.9739	3.3940

Dose 40 Gy

Patient ID	29a-3p	150-5p	101-5p	342-3p	30d-5p	320a	142-3p	191-5p	125b-5p	15b-5p
AE1	-1.2006	-1.0649	-2.4096	-0.2512	2.2808	3.5499	2.9883	1.2456	-0.9395	0.4029
BE1	0.8289	1.8921	-2.9897	2.0643	4.4735	3.9506	5.5964	4.2017	1.3789	4.5802
CG1	0.9233	1.3526	-0.3888	1.5324	3.5814	4.1442	4.8630	2.9181	1.5344	4.1289
DW1	1.6635	2.6943	-2.1896	1.9804	3.8243	4.1886	5.3411	3.3104	0.1327	4.0601
FL1	-1.3691	-0.5442		-1.5330	-0.1318	0.7614	1.5023	-0.7823	-0.3657	0.1751
GH1	0.5598	2.2225		1.0297	1.2775	2.9417	4.1233	1.7291	0.0619	1.2312
GK1	0.7928	3.0538	-0.9514	0.2246	2.3775	5.0423	5.6661	3.8426	1.7924	3.8074
GW1	1.5744	2.5946			2.5463	3.8631	5.4236	2.8814	1.2389	0.8006
KA1	0.0298	0.3420		-0.4539	-0.7314	5.4319	4.3270	-0.6803	0.9629	-0.3132
KA2	-0.2549	2.6567		1.3981	-0.0726	3.2633	3.8555	1.4144	1.2928	0.9947
KI1	1.8106	3.0716	0.9358	2.9842	4.1128	5.8530	5.4212	3.7455	2.7594	4.5653
KM1	-1.2507	0.4685		0.6330	2.2562	1.4527	3.6212	2.0275	0.4526	1.0884
LJ1	1.8994	1.3604	0.2582	1.7875	3.6417	4.7196	5.8821	4.7426	1.8671	4.8180
MS1	1.9417	0.7511	-1.1399	1.5390	3.5350	3.8498	5.4822	2.8457	0.7093	5.2128
PB1	1.5498	2.5023	-0.2059	1.2213	0.9905	3.0534	4.8351	2.0847	2.1302	2.0168
PJ1	2.4669	3.1305	-1.4141	1.2287	2.5748	3.2867	5.7315	2.5626	1.5945	3.8190
SB1	-0.4768	1.8665	-2.5694	1.3070	3.9869	4.6047	5.4585	3.2158	0.6345	3.4843
SJ1	1.9797	4.6130	0.9470	3.4878	4.9684	5.5795	5.8026	3.9828	3.8349	3.5041
SW1	-1.3800	0.2408	-1.3235	-0.4777	2.1496	2.6031	3.6104	2.3955	-0.1564	2.9497
WJ1	1.5600	2.7327	-0.7886	0.9900	2.9910	4.6041	6.1329	3.5054	1.2077	3.5048
ZA1	0.3235	4.3612	-0.6140	3.9532	5.9539	6.0625	4.7308	5.2805	3.3667	5.4751

Table S4. NormFinder results.

Validation cohort

Gene name	Stability value			
324-3p	0.006		Best gene	16-2-3p
16-2-3p	0.006		Stability value	0.006
Let 7d	0.006			
126	0.007		Best combination of two genes	16-2-3p and Let 7d
			Stability value for best combination of two genes	0.004

A549 (intracellular)

Gene name	Stability value			
Let-7d	0.248		Best gene	miR-342
miR-16-2-3p	0.255		Stability value	0.215
miR-29a	0.252			
miR-342	0.215		Best combination of two genes	Let-7d and miR-342
miR-103a	0.351		Stability value for best combination of two genes	0.129
miR-150	0.481			
U6	0.402			

NCI-H460 (intracellular)

Gene name	Stability value			
Let-7d	0.468		Best gene	miR-16-2-3p
miR-16-2-3p	0.459		Stability value	0.459
miR-29a	0.708			
miR-342	0.673		Best combination of two genes	Let-7d and miR-103a
miR-103a	0.467		Stability value for best combination of two genes	0.244
miR-150	0.660			
U6	1.373			

NCI-H1299 (intracellular)

Gene name	Stability value		Best gene	Let-7d
Let-7d	0.306		Stability value	0.306
miR-16-2-3p	1.001			
miR-29a	0.579		Best combination of two genes	Let-7d and miR-342
miR-342	0.352		Stability value for best combination of two genes	0.257
miR-103a	0.373			
miR-150	0.751			
U6	1.572			

IMR90 (intracellular)

Gene name	Stability value		Best gene	Let-7d
Let-7d	0.227		Stability value	0.227
miR-16-2-3p	0.658			
miR-29a	0.953		Best combination of two genes	Let-7d and miR-103a
miR-342	0.947		Stability value for best combination of two genes	0.198
miR-103a	0.297			
miR-150	0.901			
U6	1.046			

MRC5 (intracellular)

Gene name	Stability value		Best gene	Let-7d
Let-7d	0.355		Stability value	0.355
miR-16-2-3p	0.550			
miR-29a	0.883		Best combination of two genes	miR-29a and miR-342
miR-342	0.990		Stability value for best combination of two genes	0.234
miR-103a	0.382			
miR-150	1.038			
U6	1.389			

Table S5. Validation of miRNAs candidates. Only miR-29a and miR-150 showed statistically significant increases or decreases with RT dose in the validation cohort of 21 patients. Mean and standard deviation of normalized miRNA expression are reported. P-values were generated as described in the methods section.

	**0 Gy**	**20 Gy**	**40 Gy**	**P value**
**29a-3p**	1.16±1.09	0.43±0.65	0.67±1.25	0.032
**150-5p**	2.91±1.34	1.98±1.17	1.92±1.49	0.036
**101-5p**	-1.12±1.25	-2.02±1.57	-0.99±1.21	0.13
**342-3p**	1.83±0.99	1.15±1.00	1.23±1.35	0.14
**30d-5p**	2.76±1.06	2.57±1.56	2.69±1.72	0.92
**320a**	4.24±0.93	4.09±1.04	3.94±1.36	0.57
**142-3p**	4.84±1.18	4.68±0.90	4.78±1.16	0.90
**191-5p**	2.73±0.87	2.55±1.08	2.69±1.54	0.87
**125b-5p**	1.21±1.05	0.98±0.75	1.21±1.19	0.82
**15b-5p**	2.94±1.30	2.85±1.21	2.87±1.81	0.96

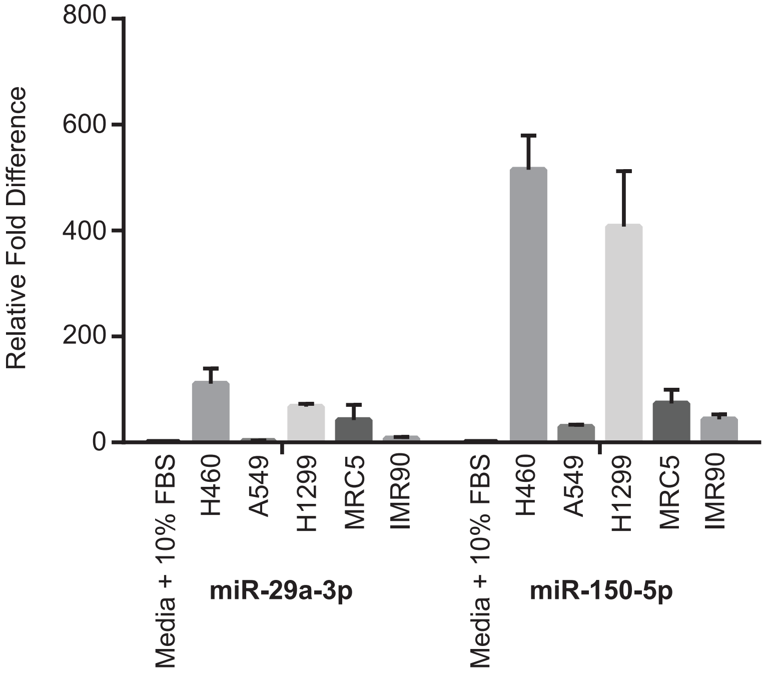

Figure S1. Background expression of miRNAs. Expression of miR-29a and miR-150 is substantially higher in exosomes isolated from cell-conditioned media compared to exosomes isolated from cell-free, FBS-containing media. Levels are reported as relative fold difference and normalized to complete media. Notably, miR-150 was undetectable in cell-free media and Ct values of 40 (upper limit) were assigned for comparison, leading to the >400 fold difference in some cultured media samples.

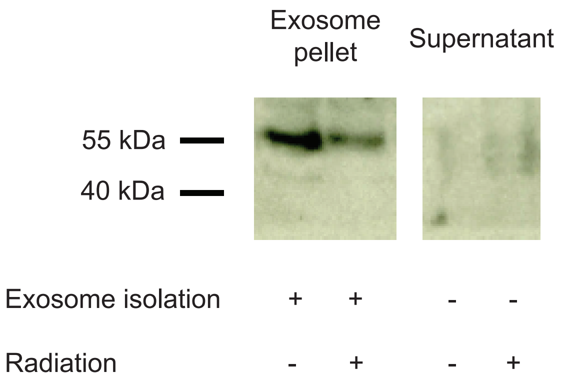

Figure S2. Exosome isolation. Western blot of conditioned media from MRC5 cells after 24 hours of cell culture, with and without radiation. Dark bands show Tsg101 (56 kDa) protein levels in the isolated exosome pellet (left) compared to the supernatant (right).

A

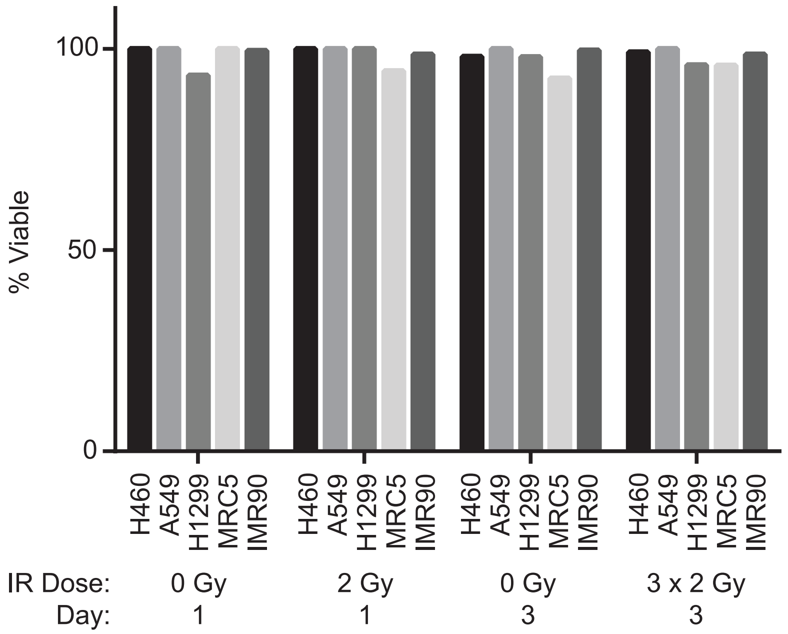

B

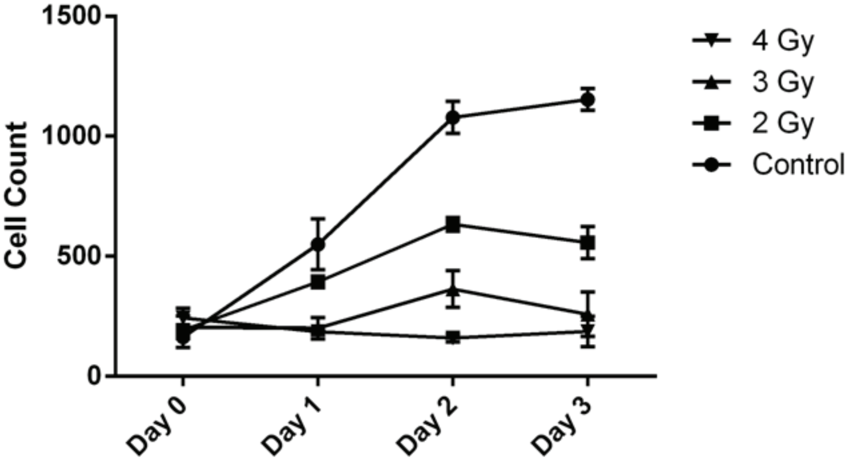

Figure S3. Viability vs. IR status. (A) Irradiation at 2 Gy fractional doses does not significantly decrease cell viability assessed by trypan blue exclusion (nonadherent cells were included in counting). There was no statistical difference in cell viability in any comparisons between groups. (B) Radiation decreases cell proliferation compared to control after a single fraction of 2, 3 or 4 Gy.

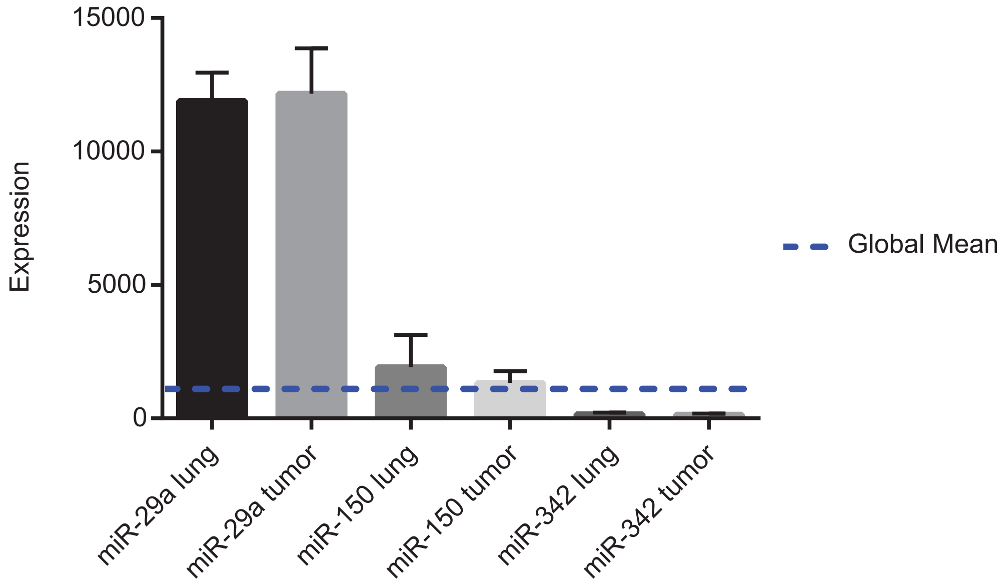

Figure S4. miRNA expression in NSCLC and lung samples. miRNA SEQ data from TCGA database of 504 patient samples. miRNA expression profiles of 456 lung tumor specimens and 48 normal lung specimens were examined. On average, miR-29a and miR-150 are highly expressed (compared to the global average expression) in both normal and tumor tissues. Other miRNAs including miR-342 were not highly expressed. Error bars indicate 95% confidence interval of all data.
